# Antithrombotic Regimens in Patients With Percutaneous Coronary Intervention Whom an Anticoagulant Is Indicated: A Systematic Review and Network Meta-Analysis

**DOI:** 10.3389/fphar.2018.01322

**Published:** 2018-11-19

**Authors:** Wipharak Bunmark, Peerawat Jinatongthai, Prin Vathesatogkit, Ammarin Thakkinstian, Christopher M. Reid, Wanwarang Wongcharoen, Nathorn Chaiyakunapruk, Surakit Nathisuwan

**Affiliations:** ^1^Department of Pharmacy, Faculty of Pharmacy, Mahidol University, Bangkok, Thailand; ^2^Pharmacy Practice Division, Faculty of Pharmaceutical Sciences, Ubon Ratchathani University, Ubon Ratchathani, Thailand; ^3^School of Pharmacy, Monash University Malaysia, Selangor, Malaysia; ^4^Department of Medicine, Faculty of Medicine, Ramathibodi Hospital, Mahidol University, Bangkok, Thailand; ^5^Section for Clinical Epidemiology and Biostatistics, Faculty of Medicine, Ramathibodi Hospital, Mahidol University, Bangkok, Thailand; ^6^School of Epidemiology and Preventive Medicine, Monash University, Melbourne, VIC, Australia; ^7^School of Public Health, Curtin University, Perth, WA, Australia; ^8^Department of Internal Medicine, Faculty of Medicine, Chiang Mai University, Chiang Mai, Thailand; ^9^Center of Pharmaceutical Outcomes Research (CPOR), Naresuan University, Phitsanulok, Thailand; ^10^School of Pharmacy, Monash University Malaysia, Selangor, Malaysia; ^11^School of Pharmacy, University of Wisconsin, Madison, WI, United States; ^12^Asian Centre for Evidence Synthesis in Population, Implementation and Clinical Outcomes (PICO), Health and Well-being Cluster, Global Asia in the 21st Century Platform, Monash University Malaysia, Bandar Sunway, Selangor, Malaysia

**Keywords:** anticoagulants, antithrombosis, myocardial infarction, network meta-analysis, percutaneous coronary intervention

## Abstract

**Background:** Patients undergoing percutaneous coronary intervention (PCI) who require anticoagulant therapy are at increased risk of bleeding. The optimal regimen for these patients is uncertain. This study aimed to compare safety and efficacy of antithrombotic regimens used in patients undergoing PCI with concomitant anticoagulant therapy.

**Methods:** A systematic review and network meta-analysis was performed among studies comparing antithrombotic regimens for anticoagulated patients undergoing PCI. The primary outcome of interest was major bleeding. The secondary outcomes were coronary events. The reference intervention was classic triple therapy (aspirin plus clopidogrel plus VKA). Cluster rank incorporating risk (major bleeding) and benefit (all-cause death) was performed to identify the most appropriate regimen(s).

**Results:** There were 3 RCTs (6 interventions) and 29 non-RCTs (8 interventions) that met the inclusion criteria with 22,179 patients. Network meta-analysis of RCTs indicated that dual therapy (DT), either with vitamin K antagonist (VKA) or direct anticoagulant (DOAC) plus an antiplatelet, significantly reduced the risk of major bleeding compared to triple therapy (TT) [pooled RR of 0.51 (0.30–0.87) and 0.68 (0.49–0.94), respectively]. In addition, VKA-DT significantly reduced the risk of all-cause death compared to TT [pooled RR of 0.40 (0.17–0.93)]. Results from network meta-analysis of non-RCT paralleled that of RCTs. No significant differences of coronary events were found.

**Conclusions:** In conclusion, for anticoagulated patients undergoing PCI, dual therapy, either with warfarin or DOAC plus an antiplatelet, should be considered due to its optimal balance on efficacy and safety.

## Introduction

Among patients who have an indication for anticoagulant therapy, approximately one third concurrently suffer from coronary artery disease where percutaneous coronary intervention (PCI) may be indicated (Dewilde et al., 2014). This situation therefore leads to a need for the concomitant use of both antiplatelet(s) and anticoagulant therapy which poses heighten risk of major bleeding (Rubboli et al., 2014a). A recent national registry suggested that the rates of fatal or nonfatal bleeding among atrial fibrillation patients admitted with myocardial infarction or for PCI significantly increased with increasing intensity of anti-thrombotic regimens. Patients receiving triple therapy [(TT): dual antiplatelet therapy (DAPT) plus an oral anticoagulant] experienced the highest bleeding rate at 14.2 events per 100 person-years with adjusted hazard ratio (HR) of 1.41 compared to dual therapy [(DT): vitamin K antagonist (VKA) plus single anti-platelet] (Lamberts et al., 2012). Major bleeding has been shown to increase 1-year mortality by several folds among PCI patients (Rao et al., 2005; Manoukian et al., 2007), most likely due to significant blood loss, hemodynamic compromise, or ischemic events secondary to the interruption or cessation of anti-thrombotic therapy.

The current situation is even more complex due to increasing usage of more potent P2Y_12_ inhibitors, namely prasugrel, and ticagrelor, and direct oral anticoagulants (DOAC), namely dabigatran, rivaroxaban, apixaban, and edoxaban. Unfortunately, direct head-to-head trials comparing efficacy and safety of these combinations are very limited despite the magnitude of the problem. Current practice guidelines are therefore based on limited data and expert consensus, recommending the use of a TT with aspirin, clopidogrel and an oral anticoagulant as the standard therapy. DT with clopidogrel and an oral anticoagulant is also recommended as an alternative for patients in whom the bleeding risk outweighs the ischemic risk (Kirchhof et al., 2016; Levine et al., 2016; Roffi et al., 2016; Valgimigli et al., 2018). However, recommendations regarding the use of newer antiplatelets and anticoagulants as a part of these regimens are still limited. We therefore performed a systematic review and network meta-analysis, where possible, to evaluate the relative efficacy and safety among various antithrombotic regimens.

## Methods

### Study design

This study was conducted following the registered protocol with PROSPERO (CRD 42017052655) and was reported according to the Preferred Reporting Items for Systematic Reviews and Meta-Analyses (PRISMA) extension statement for network meta-analysis (Hutton et al., 2015). The study protocol was approved by the Institutional Review Board of Mahidol University (COE.No. MU-DT/PY-IRB 2017/022.2906).

### Search strategy and study selection

Relevant studies were identified from MEDLINE (via PubMed), Embase, Cochrane Central Register of Control Trials (CENTRAL) and ClinicalTrials.gov since inception to October 1, 2017 the following search terms were used: “PCI,” stent, “acute coronary syndrome,” “myocardial infarction,” revascularization, anticoagulant, antithrombotic, “dual antithrombotic,” “P2Y_12_ receptor antagonist^*^,” generic and trade names of antithrombotic agents (coumarins, warfarin, dabigatran, rivaroxaban, apixaban, edoxaban, aspirin, clopidogrel, prasugrel, ticagrelor), and synonymous words. Search strategies were described in Supplementary Appendix 1. Two investigators (W.B. and P.J.) independently performed the study selection. The reviewers independently screened titles and abstracts. Discrepancies were resolved by discussion. Reference lists of selected articles were also reviewed, and efforts to contact authors were made to obtain further study details. Both randomized controlled trials (RCTs) and non-RCTs were considered without language restrictions using the following criteria: (1) studied in patients who underwent PCI and received anticoagulants for prevention or treatment of thromboembolic complications (2) compared efficacy and safety among any pair of antithrombotic regimens (DAPT, DT (aspirin or a P2Y_12_ receptor antagonist plus an anticoagulant), and TT (aspirin plus a P2Y_12_ receptor antagonist plus an anticoagulant). Studies were excluded if the period of outcome measurement was < 1 month.

### Data extraction and quality assessment

Data were extracted including study design, baseline characteristics (e.g., age, underlying diseases, details of PCI procedure, and indications of anticoagulant therapy), antithrombotic regimens both in terms of composition and drug utilization, and outcomes of interest. Authors were contacted in case of incomplete or unclear data. Quality of studies was assessed depending on type of studies. For RCTs, the Cochrane Collaboration's tool for assessing risk of bias (ROB) was used (Higgins et al., 2016). This tool is comprised of 5 domains addressing biases in the randomization process, deviations from intended interventions, missing outcome data, measurement of the outcome, and selection of the reported result. Meanwhile, the ROB in Non-randomized Studies tool (ROBINS-I) was used for non-RCTs. ROBINS-I is comprised of 7 domains addressing biases due to confounding, selection of participants, classification of interventions, deviations from intended interventions, missing data, measurement of outcomes and selection of the reported result (Sterne et al., 2016).

### Type of interventions and reclassification of regimens

Treatment regimens were combinations within/between drug classes including antiplatelet agents [aspirin (A), clopidogrel (C), ticagrelor (T), prasugrel (P)], and anticoagulants [VKA (e.g., warfarin, acenocoumarol, phenprocoumon), low molecular weight heparin (LMWH), dabigatran 150 mg BID (D), dabigatran 110 mg BID (d), rivaroxaban 15 mg OD (R), and rivaroxaban 2.5 mg BID (r)]. In addition, we reclassified these intervention into groups based on composition of regimens (Table 1). For RCT, we were able to classify regimens into 3 groups including TT (aspirin plus clopidogrel plus a VKA), VKA-DT (aspirin or a P2Y_12_ receptor antagonist plus a VKA) and DOAC-DT (aspirin or a P2Y_12_ receptor antagonist plus rivaroxaban or dabigatran). The reclassification is based on differences in the pharmacological profiles of anticoagulants (between VKAs vs. direct acting oral anticoagulants) and the intensity of antithrombotic therapy (TT vs. DT). We excluded two regimens of RCT from analysis. First was the regimen of aspirin plus clopidogrel plus 2.5 mg BID dose of rivaroxaban from PIONEER-AF PCI trial since this dose was an unapproved dose for stroke prevention (Gibson et al., 2016). Second, we extracted data from clopidogrel plus dabigatran 150 mg BID and its corresponding control arm but not from the 110 mg BID arms of the REDUAL-PCI trial. This was due to the fact that the control arm of each dabigatran dose was from the same pool of patient population with some adjustment in number and characteristics of the patients. If we included the data from both doses, it may create duplication of control arms (Cannon et al., 2017). We therefore chose to extract data from dabigatran 150 mg BID which is the most commonly approved dose worldwide. For non-RCT, based on the available data, we were able to reclassify interventions into 4 regimens including TT (aspirin plus clopidogrel plus an anticoagulant), newP2Y_12_TT (aspirin plus either prasugrel or ticagrelor plus an anticoagulant), VKA-DT (aspirin or a P2Y_12_ receptor antagonist plus a VKA), and DAPT (aspirin plus a P2Y_12_ receptor antagonist). With these reclassified regimens, we were able to evaluate the effects of newer antithrombotic therapy compared to the conventional regimens, which may potentially extend our knowledge beyond current clinical practice guidelines (Kirchhof et al., 2016; Levine et al., 2016; Roffi et al., 2016; Valgimigli et al., 2018).

**Table 1:** Detail of re-classified regimens.

**Regimens**	**Principle**	**Expected combinations**
NewP2Y12-based TT	Aspirin *plus* prasugrel/ticagrelor *plus* anticoagulant	A+P+VKA, A+T+VKA
VKA-based TT (reference therapy)	aspirin *plus* P2Y_12_ receptor antagonist *plus* anticoagulant	A+C+VKA
DOAC-based TT	Aspirin plus any P2Y_12_ receptor antagonist *plus* dabigatran/rivaroxaban/apixaban/edoxaban	A+C+D, A+C+R, A+C+apixaban, A+C+edoxaban
Dual therapy	aspirin *or* P2Y_12_ receptor antagonist *plus* any anticoagulant (VKA/DOACs)	C+VKA, P+VKA, T+VKA, C+D/R/apixaban/edpxaban, P+D/R/apixaban/edpxaban, T+D/R/apixaban/edpxaban
Dual antiplatelet	Aspirin *plus* any P2Y_12_ receptor antagonist	A+C, A+P, A+T

*A, Aspirin; C, Clopidogrel; D, Dabigatran; DOACs, Direct-acting oral anticoagulants; P, Prasugrel; T, Ticagrelor; TT, Triple therapy; R, Rivaroxaban; VKA, Vitamin K antagonist*.

### Outcomes of interest

The primary endpoint was major bleeding which was defined according to Bleeding Academic Research Consortium (BARC) type 3–5 (Mehran et al., 2011), and “compatible definition” if those could be standardized based on BARC type 3–5 criteria (see details of compatibility criteria in Supplementary Appendix 2). The secondary endpoints were stroke and/or systemic embolism, myocardial infarction, repeated revascularization, any stent thrombosis and all-cause death. In addition, we investigated the risk-benefit balance of various interventions by incorporating safety (major bleeding) and efficacy (all-cause death) using two-dimensional plots and clustering methods to rank these interventions. All-cause death was used as the efficacy outcome due to the lack of uniformity in the report of major cardiovascular events.

### Data synthesis and statistical methods

A pairwise meta-analysis and network meta-analysis were performed as follows. A pairwise meta-analysis, risk ratio (RR) along with 95% confidence interval (CI) was estimated and pooled using a random-effects model (DerSimonian and Laird, 1986). Heterogeneity was assessed using Cochrane Q test and I^2^ statistics (Maldonado et al., 2009). A network meta-analysis was performed to compare relative efficacy and safety among regimens. Relative treatment effects (RR) were estimated for each comparison vs. a common comparator of A+C+VKA or TT for re-classified. Subsequently, these RRs were pooled across studies using a meta-analysis with a consistency model (Jansen et al., 2011). We used the global inconsistency test to evaluate inconsistency in a network as a whole. If inconsistency was detected, we then used the loop-specific and node-splitting methods to identify which piece of evidence was responsible for inconsistency (Dias et al., 2010). Adjusted funnel plots were produced in order to determine small study effects (Mavridis and Salanti, 2014). The surface under the cumulative ranking curve (SUCRA) was performed to rank various antithrombotic regimens for each outcome. Finally, the cluster rank, a technique used to combine multidimensional aspects (usually risk and benefit) of an intervention, was performed to incorporate safety (major bleeding) and efficacy (all-cause death) simultaneously (Jinatongthai et al., 2017). The same approaches were used to compare 5 re-classified regimens including TT, newP2Y_12_TT, VKA-DT, DOAC-DT, and DAPT.

Pre-specified subgroup analyses were performed by patient characteristics (atrial fibrillation and atrial fibrillation predominant group, follow-up period (< 1, 1, >1 year), PCI-related predominant characteristics [i.e., ACS, elective PCI, bare-metal stent (BMS), drug-eluting stent (DES)], and study characteristics (study design and setting). Predominant groups were classified if characteristic prevalence was ≥50%. A pre-specified sensitivity analysis was performed on different major bleeding definitions and certain characteristics of studies (i.e., adjusted analysis, multicenter studies, omitting small sample size studies or serious-to-critical ROB). All analyses were stratified by non-RCTs and RCTs using STATA 14.0 (Stata Corp, College Station, TX). A *p* < 0.05 was considered statistically significant.

## Results

### Study selection

**Figure 1:**
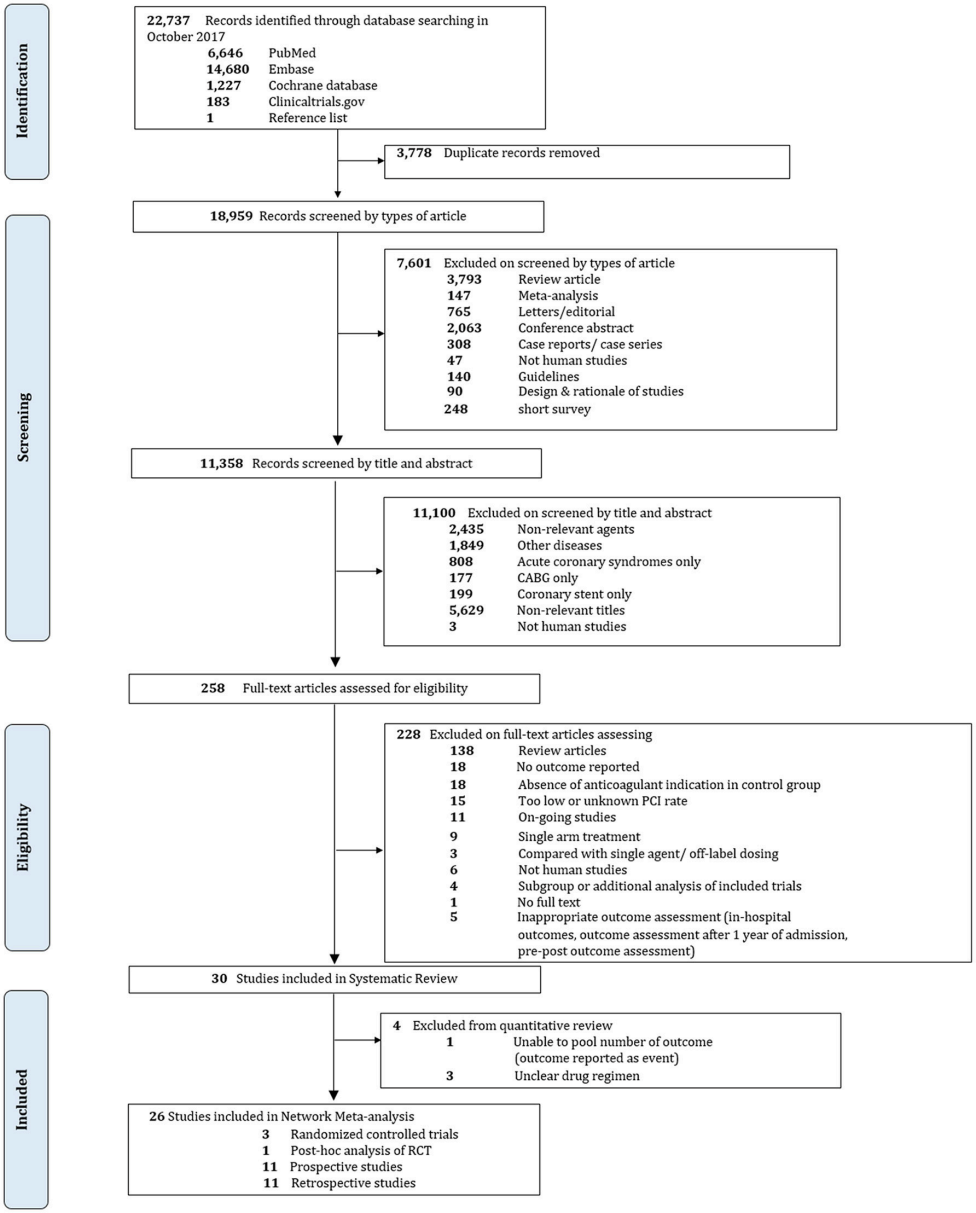
Flow diagram and references of included studies.

Overall, 22,737 records were identified, 258 potentially eligible articles were retrieved in full text. 200 and 28 articles were excluded, mostly due to absence of anticoagulant indication in the control group, contamination of patients without PCI or unknown rate of PCI, and no outcome of interest. Finally, 30 studies were included in our systematic review including 3 RCTs and 27 non-RCTs. Among 27 non-RCTs, only 23 studies were included in the quantitative analysis since 3 studies did not sufficiently specify composition of drug regimens while one study did not provide adequate outcome data. The PRISMA flow diagram is shown in Figure 1.

### Characteristics and quality of included studies

A total of 12 antithrombotic regimens were identified including 1 DAPT (A+C), 6 DTs (A+VKA, C+VKA, T+VKA, C+R, C+D, C+d), and 5 TTs (A+C+VKA, A+P+VKA, A+T+VKA, A+C+r, A+C+LMWH). Detail of each regimen was summarized in Supplementary Appendix 3. Characteristics of all included studies are shown in Table 2.

**Table 2:** Main characteristics of included studies.

**Study name/First author**	**Arms**	**Brief results (major bleeding outcome only)**	**Study design**	**No. of patients (N)**	**Procedural Characteristics**		**AF (%)**	**Follow-up time (year)**	**Adjustment method in case of non-RCTs**	**Overall risk of bias**
					**PCI due to ACS (%)**	**DES stent implanted (%)**				
**RANDOMIZED-CONTROLLED TRIALS**										
WOEST (Dewilde et al., 2013)	A+C+VKA vs. C+VKA	C+VKA significantly reduced risk of BARC type 3 bleedings by 51%	RCT	563	27.5	67.1	69.4	1	-	Some concerns
PIONEER-AF PCI (Gibson et al., 2016)	A+C+VKA vs. A+C+r vs. C+R	C+R and A+C+r significantly reduced clinically significant bleeding by 41% and 37%, respectively	RCT	2124	52.3	66.24	100	1	-	Some concerns
REDUAL-PCI (Cannon et al., 2017)	A+C+VKA vs. C+d vs. C+D	C+d and C+D significantly reduced risk of ISTH major bleedings by 48% and 38%, respectively	RCT	2725	50.5	82.73	100	1.17	-	Some concerns
**OBSERVATIONAL STUDIES**										
MUSICA (Sambola et al., 2009)	A+C+VKA vs. A/C+VKA vs. A+C	no differences in the incidence of major bleeding among the treatment groups (4.3% vs. 6.5% vs. 1.2%, *P* = 0.290)	P	405	70.9	46.2	67.6	0.5	-	Serious
Gao F (Gao et al., 2010)	A+C+VKA vs. A/C+VKA vs. A+C	no differences in the incidence of major bleeding among the treatment groups (2.9 vs. 2.5% vs. 1.8%, *P* = 0.725)	P	622	14.3	100	100	1	-	Serious
WAR-STENT (Rubboli et al., 2014a)	A+C+VKA vs. A/C+VKA vs. A+C	no differences in the incidence of major bleeding among the treatment groups (4 vs. 5% vs. 2%, *P* = 0.840)	P	401	64	33	78	1	Multivariate analysis	Serious
AFCAS (Rubboli et al., 2014b)	A+C+VKA vs. C+VKA vs. A+C	no differences in the incidence of major bleeding among the treatment groups (10 vs. 7 vs.12%, *P* = 0.430)	P	914	57	25	100	1	Multinomial logistic regression	Serious
De Vecchis R (De Vecchis et al., 2016)	A+C+VKA vs. A/C+VKA vs. A+C	no differences in the incidence of major bleeding among the treatment groups (8.3 vs. 6.45% vs. 5.3%, *P* = 0.893)	R	98	69.3	NA	75.5	1	-	Serious
Saraffoff N (Sarafoff et al., 2013)	A+C+VKA vs. A+P+VKA	A+P+VKA significantly increased risk of TIMI major and minor bleeding by 3.2 times	P	377	36.9	100	77.4	0.5	Multivariate analysis	Serious
Braun OO (Braun et al., 2015)	A+C+VKA vs. T+VKA	no differences in the incidence of major bleeding between the treatment groups (7.0 vs. 7.5%, respectively)	R	266	100	42.9	55	0.25	-	Serious
Fu A (Fu et al., 2016)	A+C+VKA vs. A+T+VKA	no differences in the incidence of major bleeding between the treatment groups (12 vs. 11.1%, respectively)	R	152	78.3	55.3	42.1	1	Multivariate analysis	Serious
GRACE (Nguyen et al., 2007)	A+C+VKA vs. A+VKA vs. C+VKA	Major bleeding was not reported as an outcome in this study	R	800	100	27	40	0.5	-	Critical
Suh SY (Suh et al., 2014)	A+C+VKA vs. A+C	This study was not included in the quantitative analysis	R	203	40.3	82.8	100	3.5	-	Serious
STENTICO (Gilard et al., 2009)	A+C+VKA vs. A+C	A+C significantly reduced risk of moderate-to-severe GUSTO bleeding (6.4 vs. 2.1%, *P* = 0.040)	P	359	75.5	30.4	69.1	1	-	Serious
REAL (Rubboli et al., 2012)	A+C+VKA vs. A+VKA vs. A+C	No significant differences of major bleeding between the treatment groups (5 vs. 2.6 vs. 2%, *P* = 0.320)	P	622	63	25	58	1	Multivariate analysis	Moderate
Ho KW (Ho et al., 2013)	A+C+VKA vs. A+C	No significant differences of major bleeding between the treatment groups (10.6 vs. 9.6%, *P* = 0.720)	R	602	69.6	NA	100	0.5	Multivariate analysis	Serious
Dabrowska M (Dabrowska et al., 2013)	A+C+VKA vs. A+C	No significant differences of major bleeding between the treatment groups (11.1 vs. 6.9%, repectively)	P	47	NA	24	100	1	-	Serious
Hess CN (Hess et al., 2015)	A+C+VKA vs. A+C	A+C significantly reduced risk of bleeding requiring hospitalization and risk of intracranial hemorrhage by 62 and 49%, respectively	R	4959	100	51.1	100	2	Inverse probability weighted propensity score	Serious
Kang DO (Kang et al., 2015)	A+C+VKA vs. A+C	A+C significantly reduced risk of major bleeding (16.7 vs. 4.6%, *P* < 0.001)	R	367	77.7	100	100	1.72	Propensity score matching	Serious
Caballero L (Caballero et al., 2013)	A+C+VKA vs. A+C	No significant differences of major bleeding between the treatment groups (20.9 vs. 21.2%, *P* = 1.00)	R	81	94.1	37.2	100	1.42	Multivariable analysis	Serious
Sambola A (Sambola et al., 2016)	A+C+VKA vs. A+C	A+C reduced risk of major bleeding (7.5 vs. 2.2%, respectively)	P	585	73.2	39.8	100	1	Multivariate analysis	Moderate
Maegdefessel L (Maegdefessel et al., 2008)	A+C+VKA vs. A+C+LMWH vs. A+C	two severe bleeding events in A+C group (0% vs 0% vs 2.1%, respectively)	R	159	86.1	NA	100	1.4	-	Serious
Saraffoff N (Sarafoff et al., 2008)	A+C+VKA vs. A+C	No significant differences of major bleeding between the treatment groups (1.4% vs 3.1%, P = 0.340)	P	515	NA	100	77.86	2	-	Low
Manzano-Fernandez S (Manzano-Fernández et al., 2008)	A+C+VKA vs. A+C	A+C significantly reduced risk of late-major bleeding (21.6% vs 3.8%; p = 0.006	R	104	90.4	66	100	1.0*	Multivariate analysis	Serious
Ruiz-Nodar JM (Ruiz-Nodar et al., 2008)	A+C+VKA vs. A+C	No significant differences of major bleeding between the treatment groups (14.9% vs 9.0%, P = 0.190)	R	426	83.9	40.1	100	595 days*	Multivariate analysis	Serious
Goto K (Goto et al., 2014)	A+C+VKA vs. A+C	This study was not included in the quantitative analysis	R	1007	37.1	47.9	100	5.1*	Multivariate analysis	Moderate
Jang SW (Jang et al., 2011)	A+C+VKA vs. A+C	This study was not included in the quantitative analysis	R	362	57.2	90.9	100	NA	Multivariate analysis	Serious
Valencia J (Valencia et al., 2008)	A+C+VKA vs. A+C	This study was not included in the quantitative analysis	P	70	74.3	60	68.6	1	-	Moderate
ISAR-TRIPLE (Fiedler et al., 2015)	A+C+VKA vs. A+VKA	A+VKA reduced risk of major bleeding (4.8% vs. 2.8%, respectively)	*Post-hoc* of RCT	614	32.1	99.8	83.9	0.75	-	Low
Choi H (Choi et al., 2017)	A+C+VKA vs. A+C	A+C significantly reduced risk of major bleedings by 78%	P	704	55.1	100	100	6.2	Inverse probability of treatment weighting	Moderate

A, Aspirin; C, Clopidogrel; D, Dabigatran (high-dose); d, dabigatran (low-dose); DOACs, Direct-acting oral anticoagulants;P, Prasugrel; T, Ticagrelor; TT, Triple therapy; R, Rivaroxaban (low-dose); r, Rivaroxaban (very low-dose); VKA, Vitamin K antagonist. RCT, randomized-controlled trial; P, prospective cohort study; R, retrospective cohort study; NA, not available; No, number; PCI, percutaneous coronary intervention; ACS, acute coronary syndrome; DES, drug-eluting stent; AF, atrial fibrillation. *Median while other values are means

For 3 RCTs involving 5,412 patients, 1 trial was 2-arm RCT comparing A+C+VKA vs. C+VKA (Dewilde et al., 2013) while the others were 3-arm RCT comparing A+C+VKA vs. C+R vs. A+C+r (Gibson et al., 2016) and A+C+VKA vs. C+D vs. C+d (Cannon et al., 2017). Study settings, applied treatment regimens and patient baseline characteristics of these trials are summarized in Supplementary Appendix 4: eTables 4.1–4.5. Quality of included RCTs based on Cochrane ROB tool was assessed, which suggested some concerns with all trials. Cause of bias in these open-labeled trials was mainly due to lack of data about protocol deviations (Supplementary Appendix 4: eTable 4.6).

Among 27 non-RCTs, there was 1 *post-hoc* analysis of RCT (Fiedler et al., 2015), 12 prospective cohorts (Sarafoff et al., 2008, 2013; Valencia et al., 2008; Gilard et al., 2009; Sambola et al., 2009, 2016; Gao et al., 2010; Rubboli et al., 2012, 2014a,b; Dabrowska et al., 2013; Choi et al., 2017), and 14 retrospective cohorts (Nguyen et al., 2007; Maegdefessel et al., 2008; Manzano-Fernández et al., 2008; Ruiz-Nodar et al., 2008; Jang et al., 2011; Caballero et al., 2013; Ho et al., 2013; Goto et al., 2014; Suh et al., 2014; Braun et al., 2015; Hess et al., 2015; Kang et al., 2015; De Vecchis et al., 2016; Fu et al., 2016). Nineteen studies were 2-arm (Manzano-Fernández et al., 2008; Ruiz-Nodar et al., 2008; Sarafoff et al., 2008, 2013; Valencia et al., 2008; Gilard et al., 2009; Jang et al., 2011; Caballero et al., 2013; Dabrowska et al., 2013; Ho et al., 2013; Goto et al., 2014; Suh et al., 2014; Braun et al., 2015; Fiedler et al., 2015; Hess et al., 2015; Kang et al., 2015; Fu et al., 2016; Sambola et al., 2016; Choi et al., 2017) and 8 studies were 3-arm comparisons (Nguyen et al., 2007; Maegdefessel et al., 2008; Sambola et al., 2009; Gao et al., 2010; Rubboli et al., 2012, 2014a,b; De Vecchis et al., 2016). All studies used A+C+VKA as the reference. A total of 8 interventions were considered including A+C, A+VKA, C+VKA, T+VKA, A+C+VKA, A+P+VKA, A+T+VKA, and A+C+LMWH. Study settings and patient baseline characteristics of these studies are summarized in Supplementary Appendix 5: eTables 5.1–5.4. Various patterns of regimens used were found, especially duration of treatment (Supplementary Appendix 5: eTable 5.5). Among non-RCTs, 7, 19, 70, and 4% of studies were with low, moderate, serious, and critical risk, respectively (Supplementary Appendix 5: eTable 5.6).

### Effect on the primary and secondary outcomes

#### RCTs

Since there were only 3 RCTs including WOEST, PIONEER AF-PCI and REDUAL-PCI, meta-analysis on RCTs was not performed since there were too few trials. However, data from these trials were extracted and used to compare 3 re-classified regimens. Results of which are reported in the re-classified regimen section (Dewilde et al., 2013; Gibson et al., 2016; Cannon et al., 2017).

#### Non-RCTs

##### Results from pairwise meta-analysis

For major bleeding, A+C significantly reduced risk of bleeding while A+P+VKA increased such risk compared to A+C+VKA with pooled RR 0.58 (0.40, 0.83) and pooled RR 5.00 (1.52, 16.67), respectively. For stroke, A+C increased risk of any stroke compared to A+C+VKA, with pooled RR 1.60 (1.04, 2.45). Overall, there was no statistically significant difference among these regimens in the risk of myocardial infarction, repeated revascularization, and stent thrombosis. For all-cause death, A+C+LMWH significantly increased the risk relative to A+C with pooled RR 4.17 (1.02, 16.67) (Supplementary Appendix 6).

##### Results from network meta-analysis

**Figure 2:**
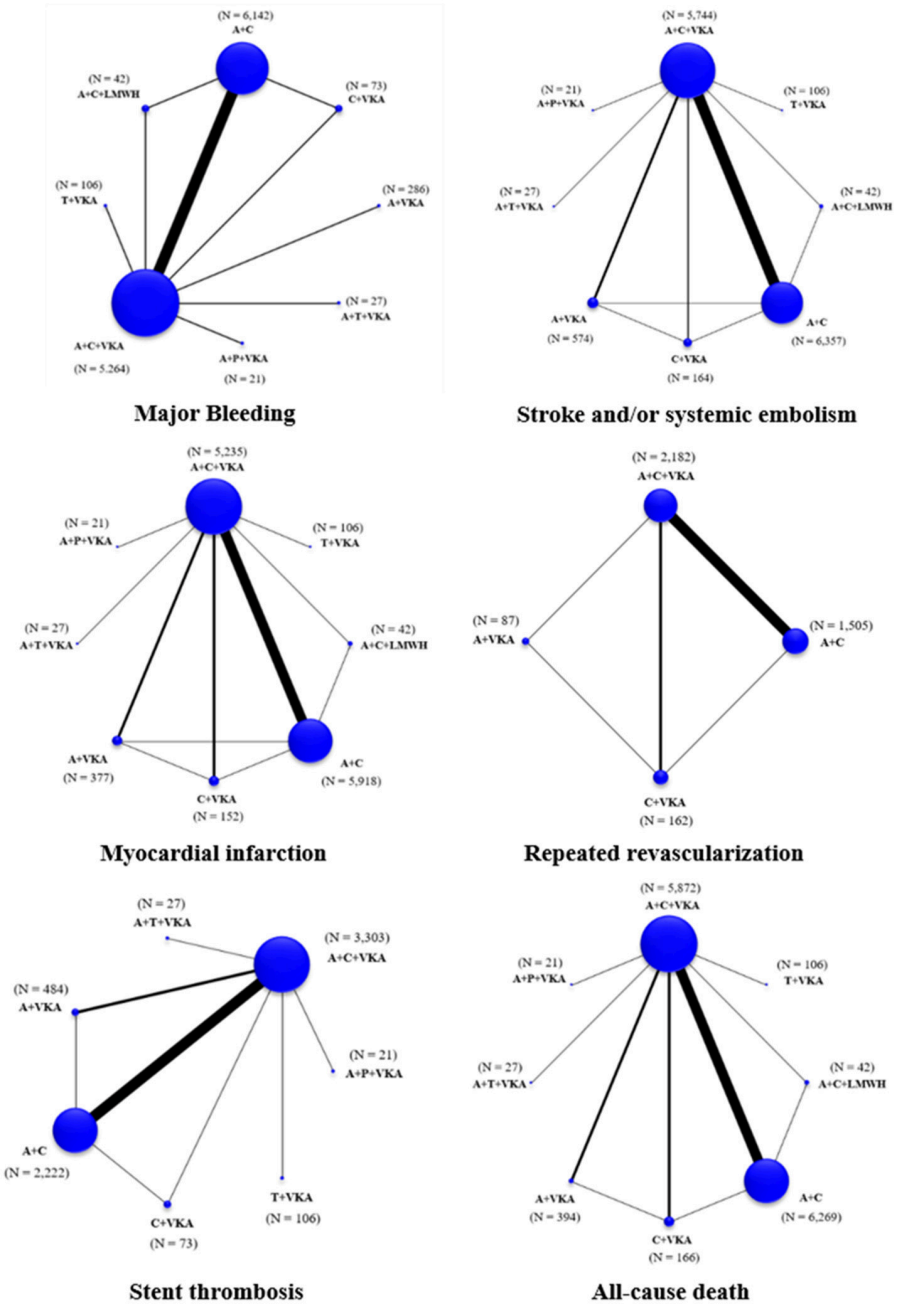
Network maps of treatment options for all outcomes. A+C, aspirin+clopidogrel; A+C+LMWH, aspirin+clopidogrel+low-molecular weight heparin; A+C+VKA, aspirin+clopidogrel+vitamin K antagonist; A+P+VKA, aspirin+prasugrel+vitamin K antagonist; A+T+VKA, aspirin+ticagrelor+vitamin K antagonist; A+VKA, aspirin+vitamin K antagonist; C+VKA, clopidogrel+vitamin K antagonist; T+VKA, ticagrelor+vitamin K antagonist.

The network of eligible comparisons for the primary outcome and secondary outcomes are shown in Figure 2. Global inconsistency was not found in each outcome (Supplementary Appendix 7). The pooled estimates of all outcomes were then based on consistency model.

**Figure 3:**
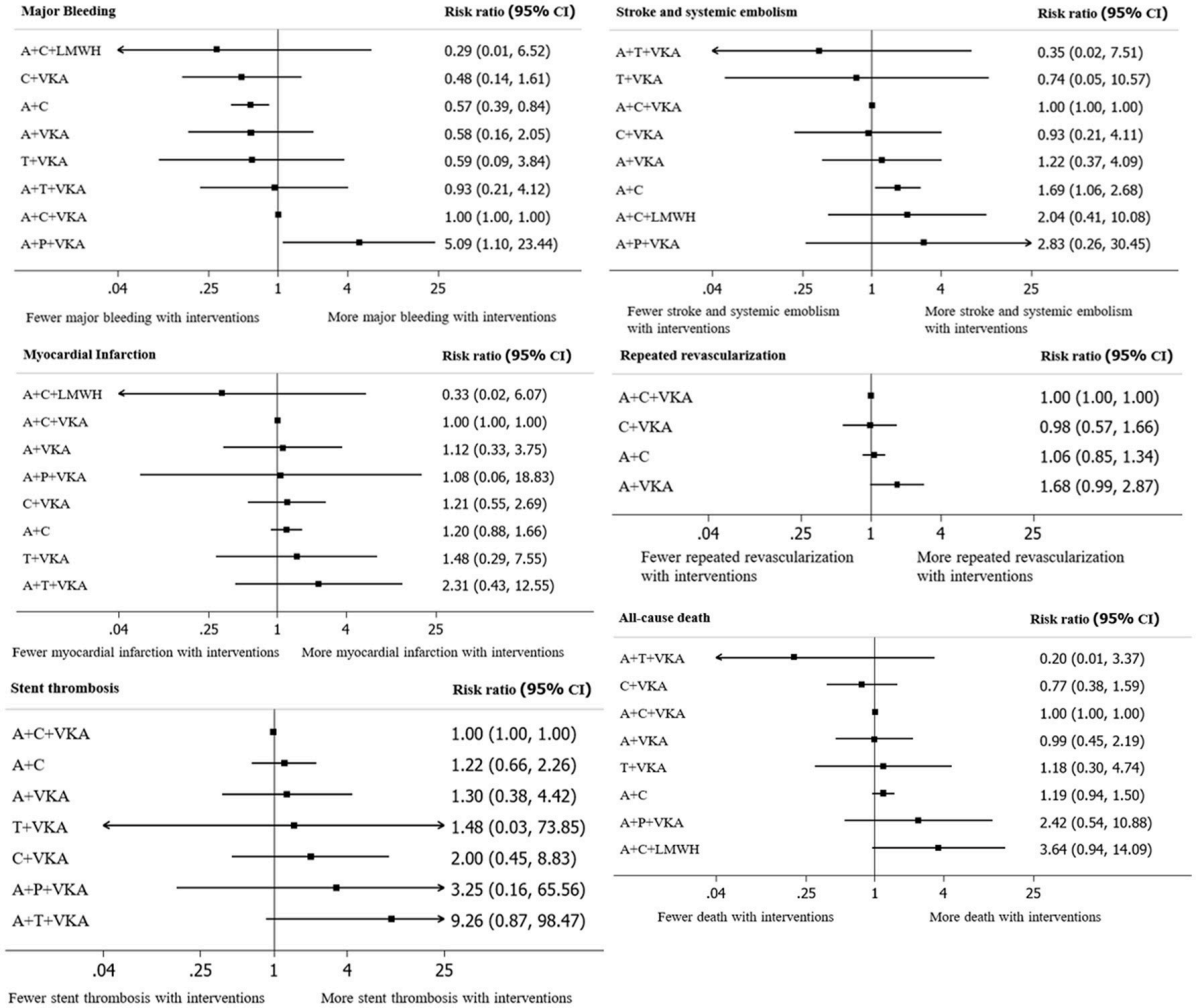
A forest plot of network meta-analysis of interventions compared with classic triple therapy (A+C+VKA). A+C, aspirin+clopidogrel; A+C+LMWH, aspirin+clopidogrel+low-molecular weight heparin; A+C+VKA, aspirin+clopidogrel+vitamin K antagonist; A+P+VKA, aspirin+prasugrel+vitamin K antagonist.

**Figure 4:**
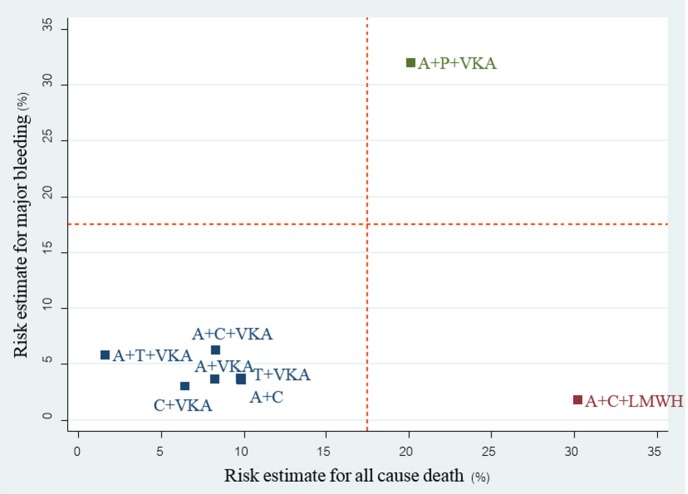
cluster rank incorporating risk estimate of major bleeding vs. all-cause death outcomes: main analysis (non-RCTs). A+C, aspirin + clopidogrel; A+C+LMWH, aspirin + clopidogrel + low-molecular weight heparin; A+C+VKA, aspirin + clopidogrel + vitamin K antagonist; A+P+VKA, aspirin + prasugrel + vitamin K antagonist; A+T+VKA, aspirin + ticagrelor + vitamin K antagonist; A+VKA, aspirin + vitamin K antagonist; C+VKA, clopidogrel + vitamin K antagonist; T+VKA, ticagrelor + vitamin K antagonist.

A total of 17 studies (*n* = 11,961) consisting of 8 interventions reported major bleeding as BARC type 3–5 or compatible definitions (Supplementary Appendix 2). Results from network meta-analysis showed that A+C significantly reduced risk of major bleeding with a pooled RR of 0.57 (0.39–0.84) while A+P+VKA significantly increased such risk with a pooled RR of 5.09 (1.10–23.44) when compared to A+C+VKA. For stroke, network meta-analyses indicated that A+C significantly increased stroke risk compared to A+C+VKA with the pooled RR of 1.69 (1.06–2.68), respectively. For myocardial infarction, repeated revascularization, and stent thrombosis, there was no statistically significant difference among all regimens in these outcomes. For all-cause death, network meta-analysis showed that A+C+LMWH significantly increased risk of all-cause death compared to C+VKA, pooled RR of 4.55 (1.08,20). The forest plot for all outcomes compared to the reference therapy (A+C+VKA) is shown in Figure 3. Further information and ranking can be found in Supplementary Appendix 8–9. For a combination of risk-benefit outcomes, the cluster rank incorporating major bleeding and all-cause death showed that A+T+VKA and C+VKA were the best regimens. A+T+VKA had the lowest mortality risk while C+VKA had the lowest risk of major bleeding. A+C+LMWH and A+P+VKA were the worst regimens since A+C+LMWH was with the highest mortality risk while A+P+VKA was with the highest risk of major bleeding (Figure 4).

### Re-classified regimen analysis

**Figure 5:**
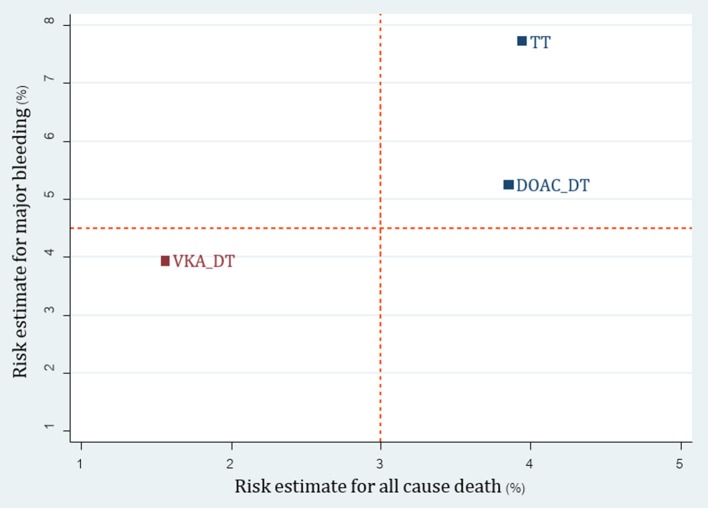
Cluster rank incorporating risk estimate of major bleeding vs. all-cause death outcomes: analysis of reclassified regimens among RCTs. DOAC, Direct-acting oral anticoagulant; DT, Dual therapy; TT, Triple therapy; VKA, Vitamin K antagonist.

For the 3 available RCTs including WOEST, PIONEER AF-PCI and REDUAL-PCI (Dewilde et al., 2013; Gibson et al., 2016; Cannon et al., 2017), we were able to compare 3 re-classified regimens including TT (A+C+VKA) vs. VKA-DT (C+VKA) vs. DOAC-DT (C+R, C+D). Results of network meta-analysis indicated that both VKA-DT and DOAC-DT significantly reduced the risk of major bleeding compared to TT [pooled RR of 0.51 (0.30–0.87); *p* = 0.014 and 0.68 (0.49–0.94); *p* = 0.02, respectively]. For stroke, myocardial infarction and stent thrombosis, there were no significant differences among these 3 regimens. However, VKA-DT significantly reduced the risk of all-cause death compared to TT [pooled RR of 0.40 (0.17–0.93); *p* = 0.034] (Supplementary Appendix 10). The cluster rank incorporating major bleeding and all-cause death showed that VKA-DT was the best regimens (Figure 5).

**Figure 6:**
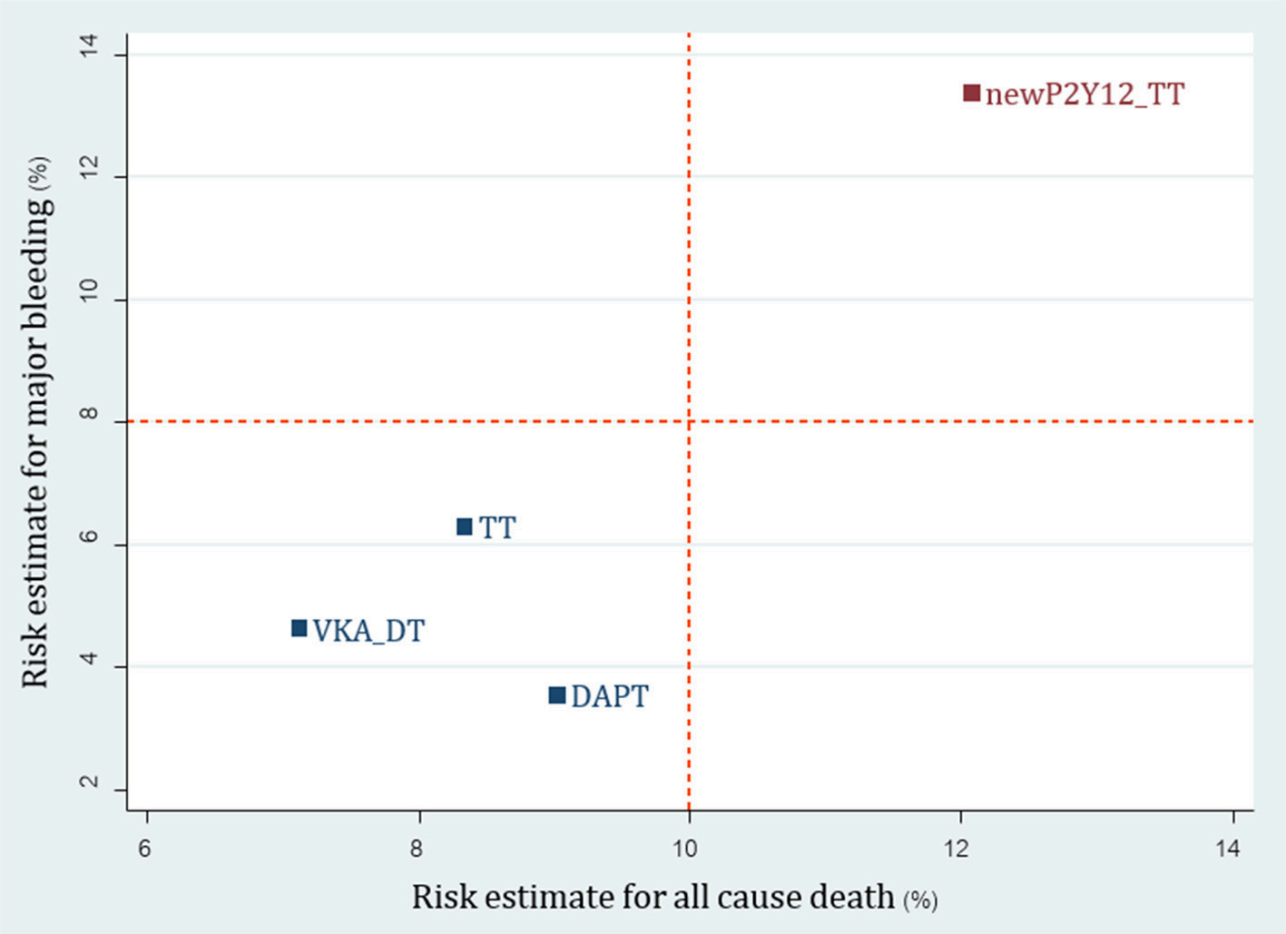
Cluster rank incorporating risk estimate of major bleeding vs. all-cause death outcomes: analysis of reclassified regimens among non-RCTs. DAPT, Dual antiplatelet; DT, Dual therapy; TT, Triple therapy; newP2Y12TT, New P2Y_12_ inhibitor-based triple therapy; VKA, Vitamin K antagonist.

For 23 non-RCTs, we reclassified 8 interventions into 4 groups including TT (A+C+VKA, A+C+LMWH), newP2Y_12_-based TT (A+P+VKA, A+T+VKA), VKA-DT (A+VKA, C+VKA, T+VKA), and DAPT (A+C). For major bleeding, SUCRA ranking showed that DAPT was the best regimen followed by DT, TT, and newP2Y_12_-based TT (Supplementary Appendix 11: eTable 11.1 and eFigure 11.1). Among efficacy outcomes, no statistical differences were found except increased risk of stroke from DAPT compared to TT with pooled RR 1.65 (1.08, 2.51) (Supplementary Appendix 11: eTable 11.2, eFigure 11.2). Based on the cluster rank of risk-benefit outcome, the best regimen was still VKA-DT in non-RCTs group (Figure 6).

### Subgroup analyses

For primary outcomes, all results from subgroup analyses (AF predominant group, ACS predominant group, stented with DES predominant group, and variety of period of follow-up) were consistent with the results in main analysis (Supplementary Appendix 12).

### Sensitivity analyses and publication bias

Sensitivity analyses with different types of major bleeding definitions were consistent with the main results (Supplementary Appendix 13: eTable 13.1). According to stroke subtypes, analysis showed that A+C reduced risk of hemorrhagic stroke compared to A+C+VKA and A+P+VKA with pooled RR 0.38 (0.16, 0.92) and pooled RR 0.02 (0.00, 0.48), respectively. We also performed sensitivity analysis among studies with adjusted RR, the results was consistent with main results. Further information from sensitivity analyses can be found in Supplementary Appendix 13. There was no clear evidence of small study effect, based on a lack of asymmetry shown in adjusted funnel plot analysis (Supplementary Appendix 14).

## Discussion

This systematic review and network meta-analysis attempts to address one of the most controversial issues in the PCI era. Accessibility of PCI is increasing worldwide, however, appropriate drug combinations for patients whom anticoagulant therapy is indicated are still unknown. The current treatment guidelines recommend the TT of aspirin, clopidogrel and an oral anticoagulant as the standard therapy (Kirchhof et al., 2016; Levine et al., 2016; Roffi et al., 2016; Valgimigli et al., 2018). However, this regimen has been shown to increase the risk of major bleeding by 40%, and among those who suffered major bleeding, there were several fold increased in mortality (Lamberts et al., 2012). The magnitude of this problem is clearly evident since approximately one third of patients requiring anticoagulant therapy may require PCI (Dewilde et al., 2014). Attempts therefore have been made to find an alternative regimen that can prevent both stroke and coronary events while minimize the risk of bleeding. Based on several RCTs and observational studies, DT with clopidogrel and an oral anticoagulant has been shown to reduce bleeding. However, its ability to reduce both stroke and coronary events is less than certain since all trials did not have sufficient power to detect differences in stroke and coronary events. Despite such limitation, the current practice guidelines still recommend DT as a viable option in patients with high bleeding risk. In addition, data regarding the newer antiplatelets and anticoagulants were quite limited at the time when the guidelines were written. Therefore, a more comprehensive and updated analysis is needed to answer some of these issues.

To the best of our knowledge, our study is the first that compared regimens containing DOACs and new P2Y_12_ inhibitors using network meta-analysis. In addition, we tried to overcome the issue of varied bleeding definitions across studies by matching the definition of bleeding events reported in each trial with the BARC definition before including those studies into the primary outcome analysis. We also performed sensitivity analyses to assess the robustness of our conclusions based on different major bleeding definitions (Mehran et al., 2011). This is a key strength of this analysis compared to previous works.

Based on analysis of non-RCTs, A+P+VKA increased risk of major bleeding compared to most regimens. Furthermore, A+P+VKA tended to increase risk of hemorrhagic stroke in sensitivity analysis based on type of stroke. This finding paralleled the result of the TRITON-TIMI-38 (Wiviott et al., 2007). Although population and drug regimens in our analysis and TRITON-TIMI 38 are not identical, caution must be raised regarding the employment of prasugrel-based regimen.

Our results for both pairwise and network meta-analyses indicated that A+C showed the lowest risk of major and any bleedings but it increased risk of stroke (RR = 1.69, 1.06–2.68). In ACTIVE-W trials, which investigated efficacy of this regimen vs. warfarin in patients with atrial fibrillation, A+C showed higher risk of stroke compared to warfarin (RR = 1.44, 1.18–1.76) (Connolly et al., 2006). Therefore, our result confirms the beneficial effect of anticoagulant therapy in patients with high thromboembolic risk.

Although we found no differences among all regimens in coronary outcomes, A+C+VKA was the most efficacious regimen in coronary outcomes based on SUCRA ranking. Risk-benefit outcome incorporating major bleeding and all-cause death showed that A+T+VKA and C+VKA were the most appropriate regimens. However, we caution readers to consider interpreting this finding carefully. The data of A+T+VKA was based entirely on a small observational study with only 27 patients using this intervention (Fu et al., 2016). In addition, beneficial effects of C+VKA among non-RCTs parallel the result of C+VKA in WOEST trial in terms of all-cause death reduction (Dewilde et al., 2013). As a result, C+VKA may be the best regimen based on our analysis.

With network meta-analysis and reclassification of antithrombotic regimens, we were able to perform analysis on the safety and efficacy of new P2Y_12_-based TT and DOACs-DT compared to conventional regimens. Based on RCTs, we were able to show that DOAC-DT significantly reduce major bleeding compared to TT and ranked favorably compared to TT when considering both major bleeding and all-cause death. Although VKA-DT was ranked best in risk-benefit outcome, we cautioned readers that this may be due to different patient characteristics along with types of antiplatelet used in WOEST compared to other trials. While all patients in both PIONEER AF-PCI and REDUAL-PCI were on anticoagulant therapy for at least 1 year, only 90% of patients in WOEST were on anticoagulant for 1 year. This may be due to the fact that WOEST trial included patients who were on anticoagulation for shorter term such as venous thromboembolism and apical thrombus. With shorter duration of treatment, bleeding rates may be lower compared to patients requiring life-long therapy in both PIONEER AF-PCI and REDUAL-PCI trials. In addition, newer and more potent antiplatelets were used in both PIONEER AF-PCI and REDUAL-PCI while clopidogrel was exclusively used in WOEST trial. Both issues may partly explain higher bleeding rates in PIONEER AF-PCI and REDUAL-PCI. For non-RCTs, cluster rank indicated that a new P2Y_12_-based TT may not be an appropriate option due to higher risk of both bleeding and all-cause death compared to all other regimens.

Prior to our study, there were a number of meta-analyses and one network meta-analysis evaluating the same issue (Bavishi et al., 2016; Briasoulis et al., 2016; Palla et al., 2016). Results from pairwise meta-analyses were with conflicting results. This is most likely due to difference of included studies and definition of major bleeding in each meta-analysis. For network meta-analysis, Liu et al. previously compared efficacy and safety of DAPT, A+VKA, C+VKA, and A+C+VKA (Liu et al., 2016). Our analysis was different in many aspects. First, we considered and included newer agents that have become increasingly used in clinical practice such as new P2Y_12_ inhibitors and DOACs. Second, we only included trials which all patients received PCI, while the previous study included trials which contained some populations who did not undergo PCI. Finally, previous analysis accepted major bleeding definition according to the original articles while we standardized bleeding based on the BARC definition.

## Study limitations

Our study has several key limitations. First, the majority of the data included in our analysis came from observational studies. Therefore, relative treatment effects were susceptible to the influence of confounding factors. Second, analysis of baseline characteristics and subgroup analyses were based on data of study level, not individual patient data level. Therefore, we could assess the data as “predominant characteristics,” which mean some contamination existed in some subgroup analyses. The reclassification of BARC bleeding was also done at a study level, not patient data level. Thirdly, due to a sparse number of studies of each combination, except A+C and A+C+VKA, our results depended mainly upon indirect comparisons from the network meta-analysis. Some findings were statistically significant with wide confidence intervals due to small sample size in each individual study. Therefore, results of our study are for hypothesis generation only. Lastly, we were unable to make any adjustment on the variation of treatment duration of each regimen. This may introduce heterogeneity on treatment duration of each regimen since regimen switching cannot be ruled out. Therefore, we could not avoid contaminating treatment effect at the point of outcome measurement in many studies. In addition, lack of information on time in therapeutic range for VKA therapy in each study may potentially affect the outcome. These limitations highlight the need for more high quality evidence for this controversial issue. Currently, there are several RCT being conducted to assess the efficacy and safety of DOACs and new P2Y_12_ inhibitor-based regimen including AUGUSTUS with apixaban, ENTRUST-AF PCI with edoxaban and MANJUSRI with ticagrelor (Lu et al., 2015; Vranckx et al., 2018). Results from these upcoming RCTs will add more information in the future. Until those high quality data become available, our systematic review may offer the most comprehensive data set and provide some guidance to tackle this issue.

## Conclusion

In summary, our analysis shows that dual therapy, either with VKA or DOAC plus a single antiplatelet, may be an attractive option for patients with PCI whom anticoagulant are indicated. DT may offer an optimal balance on safety and efficacy by lowering risk of bleeding while maintaining antithrombotic effects both from stroke/systemic embolism and coronary events post PCI, compared to TT. However, more trials are warranted to clarify this issue.

## Author contributions

WB was responsible for concept and design, analysis, interpretation of data, critical writing, and final approval of the manuscript. PJ was responsible for concept and design, analysis, and final approval of the manuscript. PV was responsible for concept and design and final approval of the manuscript. AT was responsible for concept and design, revising the intellectual content, and final approval of the manuscript. CR was responsible for revising the intellectual content, and final approval of the manuscript. WW was responsible for concept and design, revising the intellectual content, and final approval of the manuscript. NC was responsible for concept and design, interpretation of data, revising the intellectual content, and final approval of the manuscript. SN was responsible for concept and design, interpretation of data, critical writing, revising the intellectual content, and final approval of the manuscript.

### Conflict of interest statement

The authors declare that the research was conducted in the absence of any commercial or financial relationships that could be construed as a potential conflict of interest.

## Abbreviations

A+C: Aspirin + Clopidogrel
A+C+LMWH: Aspirin + Clopidogrel + Low-molecular weight heparin
A+C+r: Aspirin + Clopidogrel + Rivaroxaban 5 mg twice daily
A+C+VKA: Aspirin + Clopidogrel + Vitamin K antagonist
A+P+VKA: Aspirin + Prasugrel + Vitamin K antagonist
A+T+VKA: Aspirin + Ticagrelor + Vitamin K antagonist
A+VKA: Aspirin + Vitamin K antagonist
BARC: Bleeding Academic Research Consortium
C+D: Clopidogrel + Dabigatran 150 mg twice daily
C+d: Clopidogrel + Dabigatran 110 mg twice daily
C+R: Clopidogrel + Rivaroxaban 15 mg once daily
C+VKA: Clopidogrel + Vitamin K antagonist
DT: Dual therapy (single antiplatelet + single anticoagulant)
SUCRA: The Surface Under the Cumulative Ranking curve
T+VKA: Ticagrelor + Viatamin K antagonist
TT: Triple therapy (dual antiplatelet + single anticoagulant).
