# Supplementary material for: Antithrombotic Regimens in Patients With Percutaneous Coronary Intervention Whom an Anticoagulant Is Indicated: A Systematic Review and Network Meta-Analysis
Source: Front Pharmacol. 2018 Nov 19;9:1322. doi: 10.3389/fphar.2018.01322 (PMC6252311; doi:10.3389/fphar.2018.01322)
Supplement: Supplementary file 1 [file Data_Sheet_1.docx]

**Electronic Supplementary Material**

**Antithrombotic Regimens in Patients Undergoing Percutaneous Coronary Intervention Whom An Anticoagulant Is Indicated- A Systematic Review and Network Meta-analysis**

Bunmark W, Jinatongthai P, Vathesatogkit P, Wongcharoen W, Thakkinstian A, Reid CM, Chaiyakunapruk N, Nathisuwan S

March 16^th^, 2018

**Online Supplementary Content**

[Appendix 1 5](#_Toc529258388)

[eTable 1 Search algorithms 5](#_Toc529258389)

[Appendix 2 8](#_Toc529258390)

[eTable 2 Major bleeding definition of each study and compatibility with BARC 3-5, TIMI major, GUSTO severe, and ISTH major definitions 8](#_Toc529258391)

[Appendix 3 12](#_Toc529258392)

[eTable 3 General characteristics of treatment options 12](#_Toc529258393)

[Appendix 4 13](#_Toc529258394)

[eTable 4.1 Description of included RCTs 13](#_Toc529258395)

[eTable 4.2 Description of participants of included RCTs 14](#_Toc529258396)

[eTable 4.3 Procedural Characteristics (RCTs) 15](#_Toc529258397)

[eTable 4.4 Indication of anticoagulant therapy & Risk scores (CHADS_2_ score & HASBLED score) among RCTs 16](#_Toc529258398)

[eTable 4.5 Detail of regimens used in each study of RCTs 17](#_Toc529258399)

[eTable 4.6 Summarized risk of bias of included RCTs 18](#_Toc529258400)

[Appendix 5 19](#_Toc529258401)

[eTable 5.1 Description of included non-RCTs 19](#_Toc529258402)

[eTable 5.2 Description of participants of included non-RCTs 22](#_Toc529258403)

[eTable 5.3 Procedural Characteristics (non-RCTs) 25](#_Toc529258404)

[eTable 5.4 Indication of anticoagulant therapy & Risk scores (CHADS_2_ score & HASBLED score) among non-RCTs 29](#_Toc529258405)

[eTable 5.5 Detail of regimens used in each study of non-RCTs 32](#_Toc529258406)

[eTable 5.6 Summarized risk of bias of included non-RCTs using ROBINS-I (Risk Of Bias In Non-randomized Studies - of Interventions) tool 36](#_Toc529258407)

[Appendix 6 37](#_Toc529258408)

[eTable 6 Pairwise meta-analysis risk ratio (and 95% CI) for all dichotomous outcomes 37](#_Toc529258409)

[Appendix 7 39](#_Toc529258410)

[eTable 7 Assessment of global inconsistency in networks using the ‘design-by-treatment’ interaction model 39](#_Toc529258411)

[Appendix 8 40](#_Toc529258412)

[eTable 8.1 Results of network meta-analysis of treatment options on major bleeding 41](#_Toc529258413)

[eFigure 8.1 Network estimated risk ratios (95% confidence intervals) of treatment options on major bleeding 42](#_Toc529258414)

[eTable 8.2 Results of network meta-analysis of treatment options on stroke and/ or systemic embolism 43](#_Toc529258415)

[eFigure 8.2 Network estimated risk ratios (95% confidence intervals) of treatment options on stroke and/ or systemic embolism 44](#_Toc529258416)

[eTable 8.3 Results of network meta-analysis of treatment options on myocardial infarction 45](#_Toc529258417)

[eFigure 8.3 Network estimated risk ratios (95% confidence intervals) of treatment options on myocardial infarction 46](#_Toc529258418)

[eTable 8.4 Results of network meta-analysis of treatment options on repeated revascularization 47](#_Toc529258419)

[eFigure 8.4 Network estimated risk ratios (95% confidence intervals) of treatment options on repeated revascularization 47](#_Toc529258420)

[eTable 8.5 Results of network meta-analysis of treatment options on stent thrombosis 48](#_Toc529258421)

[eFigure 8.5 Network estimated risk ratios (95% confidence intervals) of treatment options on stent thrombosis 49](#_Toc529258422)

[eTable 8.6 Results of network meta-analysis of treatment options on all-cause death 50](#_Toc529258423)

[eFigure 8.6 Network estimated risk ratios (95% confidence intervals) of treatment options on all-cause death 51](#_Toc529258424)

[Appendix 9 52](#_Toc529258425)

[eFigure 9.1 SUCRA ranking curve for major bleeding 52](#_Toc529258426)

[eFigure 9.2 SUCRA ranking curve for stroke and/or systemic embolism 53](#_Toc529258427)

[eFigure 9.3 SUCRA ranking curve for myocardial infarction 54](#_Toc529258428)

[eFigure 9.4 SUCRA ranking curve for repeated revascularization 55](#_Toc529258429)

[eFigure 9.5 SUCRA ranking curve for stent thrombosis 56](#_Toc529258430)

[eFigure 9.6 SUCRA ranking curve for all-cause death 57](#_Toc529258431)

[Appendix 10 58](#_Toc529258432)

[eTable 10.1 Analysis of re-classified regimens on major bleeding 58](#_Toc529258433)

[eFigure 10.1 Network estimated risk ratios (95% confidence intervals) of re-classified regimens on major bleeding 58](#_Toc529258434)

[eTable 10.2 Analysis of re-classified regimens on stroke and/or systemic embolism 59](#_Toc529258435)

[eFigure 10.2 Network estimated risk ratios (95% confidence intervals) of re-classified regimens on stroke and/or systemic embolism 59](#_Toc529258436)

[eTable 10.3 Analysis of re-classified regimens on myocardial infarction 60](#_Toc529258437)

[eFigure 10.3 Network estimated risk ratios (95% confidence intervals) of re-classified regimens on myocardial infarction 60](#_Toc529258438)

[eTable 10.4 Analysis of re-classified regimens on stent thrombosis 61](#_Toc529258439)

[eFigure 10.4 Network estimated risk ratios (95% confidence intervals) of re-classified regimens on stent thrombosis 61](#_Toc529258440)

[eTable 10.5 Analysis of re-classified regimens on all-cause death 62](#_Toc529258441)

[eFigure 10.5 Network estimated risk ratios (95% confidence intervals) of re-classified regimens on all-cause death 62](#_Toc529258442)

[Appendix 11 63](#_Toc529258443)

[eTable 11.1 Analysis of re-classified regimens on major bleeding outcome 63](#_Toc529258444)

[eFigure 11.1 Network estimated risk ratios (95% confidence intervals) of re-classified regimens on major bleeding outcome 63](#_Toc529258445)

[eTable 11.2 Analysis of re-classified regimens on stroke and/ or systemic embolism 64](#_Toc529258446)

[eFigure 11.2 Network estimated risk ratios (95% confidence intervals) of re-classified regimens on stroke and/ or systemic embolism 64](#_Toc529258447)

[eTable 11.3 Analysis of re-classified regimens on myocardial infarction 65](#_Toc529258448)

[eFigure 11.3 Network estimated risk ratios (95% confidence intervals) of re-classified regimens on myocardial infarction 65](#_Toc529258449)

[eTable 11.4 Analysis of re-classified regimens on repeated revascularization 66](#_Toc529258450)

[eFigure 11.4 Network estimated risk ratios (95% confidence intervals) of re-classified regimens on repeated revascularization 66](#_Toc529258451)

[eTable 11.5 Analysis of re-classified regimens on stent thrombosis 67](#_Toc529258452)

[eFigure 11.5 Network estimated risk ratios (95% confidence intervals) of re-classified regimens on stent thrombosis 67](#_Toc529258453)

[eTable 11.6 Analysis of re-classified regimens on all-cause death 68](#_Toc529258454)

[eFigure 11.6 Network estimated risk ratios (95% confidence intervals) of re-classified regimens on all-cause death 68](#_Toc529258455)

[Appendix 12 69](#_Toc529258456)

[eTable 12.1 Subgroup analyses for the risk of major bleeding with treatment options in different groups of population 69](#_Toc529258457)

[eTable 12.2 Subgroup analyses for the risk of major bleeding with treatment options based on study characteristics 70](#_Toc529258458)

[Appendix 13 71](#_Toc529258459)

[eTable 13.1 Sensitivity analyses for the risk of major bleeding with treatment options in the variety of major bleeding definitions 71](#_Toc529258460)

[eFigure 13.1 Network estimated risk ratios (95% confidence intervals) of treatment options of major bleeding according to TIMI (major) definition 72](#_Toc529258461)

[eFigure 13.2 Network estimated risk ratios (95% confidence intervals) of treatment options of major bleeding according to ISTH (major) definition 73](#_Toc529258462)

[eTable 13.2 Sensitivity analyses for the risk of major bleeding with treatment options of different study characteristics 74](#_Toc529258463)

[eFigure 13.3 Network estimated risk ratios (95% confidence intervals) of treatment options on major bleeding by omitting studies with small sample size (less than 25^th^ percentile of populations) 75](#_Toc529258464)

[eFigure 13.4 Network estimated risk ratios (95% confidence intervals) of treatment options on major bleeding by omitting studies with serious-to-critical risk of ROBs 76](#_Toc529258465)

[eFigure 13.5 Network estimated risk ratios (95% confidence intervals) of treatment options on major bleeding by inclusion of multicenter studies 77](#_Toc529258466)

[eTable 13.3 Sensitivity analysis of type of stroke: ischemic stroke 78](#_Toc529258467)

[eFigure 13.6 Network estimated risk ratios (95% confidence intervals) of ischemic stroke 78](#_Toc529258468)

[eTable 13.4 Sensitivity analysis of type of stroke: hemorrhagic stroke 79](#_Toc529258469)

[eFigure 13.7 Network estimated risk ratios (95% confidence intervals) of hemorrhagic stroke 79](#_Toc529258470)

[Appendix 14 80](#_Toc529258471)

[eFigure 14.1 Comparison-adjusted funnel plot for the network of major bleeding in all comparisons 80](#_Toc529258472)

[eFigure 14.2 Comparison-adjusted funnel plot for the network of stroke and/or systemic embolism in all comparisons 81](#_Toc529258473)

[eFigure 14.3 Comparison-adjusted funnel plot for the network of myocardial infarction in all comparisons 82](#_Toc529258474)

[eFigure 14.4 Comparison-adjusted funnel plot for the network of repeated revascularization in all comparisons 83](#_Toc529258475)

[eFigure 14.5 Comparison-adjusted funnel plot for the network of stent thrombosis in all comparisons 84](#_Toc529258476)

[eFigure 14.6 Comparison-adjusted funnel plot for the network of all-cause death in all comparisons 85](#_Toc529258477)

[Appendix 15 86](#_Toc529258478)

[References of included studies 86](#_Toc529258479)

# Appendix 1

**Search strategies**

## eTable 1 Search algorithms

| **Database** | **Step** | **Search algorithm** | **Items found** |
| --- | --- | --- | --- |
| **PubMed** | #1 | anticoagulant | 219060 |
|  | #2 | coumarins | 45541 |
|  | #3 | warfarin | 24887 |
|  | #4 | dabigatran | 3309 |
|  | #5 | rivaroxaban | 2878 |
|  | #6 | apixaban | 1785 |
|  | #7 | edoxaban | 676 |
|  | #8 | #1 OR #2 OR #3 OR #4 OR #5 OR #6 OR #7 | 246843 |
|  | #9 | myocardial ischemia | 429867 |
|  | #10 | “percutaneous coronary intervention” | 51380 |
|  | #11 | stent | 84091 |
|  | #12 | acute coronary syndrome | 23851 |
|  | #13 | coronary artery disease | 14092 |
|  | #14 | myocardial infarction | 21631 |
|  | #15 | angioplasty | 70964 |
|  | #16 | revascularization | 47816 |
|  | #17 | #9 OR #10 OR #11 OR #12 OR #13 OR #14 OR #15 OR #16 | 559051 |
|  | #18 | aspirin | 128722 |
|  | #19 | platelet aggregation inhibitors | 128722 |
|  | #20 | antithrombotic | 14560 |
|  | #21 | dual antiplatelet | 3102 |
|  | #22 | dual antithrombotic | 456 |
|  | #23 | double antiplatelet | 930 |
|  | #24 | double antithrombotic | 527 |
|  | #25 | clopidogrel | 11367 |
|  | #26 | prasugrel | 1688 |
|  | #27 | ticagrelor | 1390 |
|  | #28 | P2Y12 receptor antagonist* | 518 |
|  | #29 | #18 OR #19 OR #20 OR #21 OR #22 OR #23 OR #24 OR #25 OR #26 OR #27 OR #28 OR #29 | 156053 |
|  | #30 | #8 AND #17 AND #29 | 7020 |
|  | #31 | Filters: Human  Update search since Oct 11, 2016 to Oct 1, 2017 | **6553**  **93**  **6646** |
| **Embase** | #1 | anticoagulant | 145417 |
|  | #2 | coumarins | 4567 |
|  | #3 | vitamin K antagonist | 2366 |
|  | #4 | warfarin | 79392 |
|  | #5 | dabigatran | 9753 |
|  | #6 | rivaroxaban | 9029 |
|  | #7 | apixaban | 5774 |
|  | #8 | edoxaban | 1912 |
|  | #9 | #1 OR #2 OR #3 OR #4 OR #5 OR #6 OR #7 OR #8 | 199425 |
|  | #10 | myocardial ischemia | 33607 |
|  | #11 | percutaneous coronary intervention | 59624 |
|  | #12 | stent | 151432 |
|  | #13 | acute coronary syndrome | 49526 |
|  | #14 | coronary artery disease | 206830 |
|  | #15 | myocardial infarction | 230660 |
|  | #16 | angioplasty | 85594 |
|  | #17 | revascularization | 86235 |
| **Embase** | #18 | #10 OR #11 OR #12 OR #13 OR #14 OR #15 OR #16 OR #17 | 629114 |
|  | #19 | aspirin | 103919 |
|  | #20 | antiplatelet* | 33737 |
|  | #21 | antithrombotic* | 21329 |
|  | #22 | dual antiplatelet* | 5716 |
|  | #23 | dual antithrombotic* | 62 |
|  | #24 | double antiplatelet* | 290 |
|  | #25 | double antithrombotic | 8 |
|  | #26 | clopidogrel | 48388 |
|  | #27 | prasugrel | 5951 |
|  | #28 | ticagrelor | 4640 |
|  | #29 | P2Y12 receptor antagonist* | 653 |
|  | #30 | #19 OR #20 OR #21 OR #22 OR #23 OR #24 OR #25 OR #26 OR #27 OR #28 OR #29 OR #30 | 162216 |
|  | #31 | #9 AND #18 AND #30 | 14863 |
|  | #32 | Limit #31 to human  Update search since Oct 11, 2016 to Oct 1, 2017 | **14074**  **606**  **14680** |
| **CENTRAL** | #1 | anticoagulant | 7792 |
|  | #2 | vitamin K antagonist* | 612 |
|  | #3 | warfarin | 3483 |
|  | #4 | dabigatran | 468 |
|  | #5 | rivaroxaban | 578 |
|  | #6 | apixaban | 346 |
|  | #7 | edoxaban | 152 |
|  | #8 | #1 OR #2 OR #3 OR #4 OR #5 OR #6 OR #7 | 7100 |
|  | #9 | myocardial ischemia | 5255 |
|  | #10 | percutaneous coronary intervention | 5939 |
|  | #11 | stent | 6677 |
|  | #12 | acute coronary syndrome | 3561 |
|  | #13 | coronary artery disease | 13915 |
|  | #14 | myocardial infarction | 21801 |
|  | #15 | angioplasty | 7457 |
|  | #16 | revascularization | 6452 |
|  | #17 | #9 OR #10 OR #11 OR #12 OR #13 OR #14 OR #15 OR #16 | 41183 |
|  | #18 | aspirin | 10079 |
|  | #19 | antiplatelet* | 3209 |
|  | #20 | antithrombotic* | 1663 |
|  | #21 | dual antiplatelet* | 717 |
|  | #22 | dual antithrombotic* | 73 |
|  | #23 | double antiplatelet* | 941 |
|  | #24 | double antithrombotic* | 546 |
|  | #25 | clopidogrel | 3306 |
|  | #26 | prasugrel | 499 |
|  | #27 | ticagrelor | 423 |
|  | #28 | P2Y12 receptor antagonist* | 72 |
|  | #29 | #18 OR #19 OR #20 OR #21 OR #22 OR #23 OR #24 OR #25 OR #26 OR #27 OR #28 | 14256 |
|  | #30 | #8 AND #17 AND #29  Update search since Oct 2016 to Oct 2017 | **1177**  **50**  **1227** |
| **ClinicalTrials.gov** | #1 | anticoagulant | 2632 |
|  | #2 | vitamin K antagonist | 198 |
|  | #3 | warfarin | 532 |
|  | #4 | dabigatran | 227 |
|  | #5 | rivaroxaban | 245 |
|  | #6 | apixaban | 135 |
|  | #7 | edoxaban | 44 |
| **ClinicalTrials.gov** | #8 | #1 OR #2 OR #3 OR #4 OR #5 OR #6 OR #7 | 2763 |
|  | #9 | myocardial ischemia | 6619 |
|  | #10 | percutaneous coronary intervention | 1725 |
|  | #11 | stent | 2647 |
|  | #12 | acute coronary syndrome | 1527 |
|  | #13 | coronary artery disease | 8918 |
|  | #14 | myocardial infarction | 3868 |
|  | #15 | angioplasty | 1025 |
|  | #16 | revascularization | 1846 |
|  | #17 | #9 OR #10 OR #11 OR #12 OR #13 OR #14 OR #15 OR #16 | 11971 |
|  | #18 | aspirin | 1390 |
|  | #19 | antiplatelet | 839 |
|  | #20 | antithrombotic | 248 |
|  | #21 | dual antiplatelet | 247 |
|  | #22 | dual antithrombotic | 32 |
|  | #23 | double antiplatelet | 181 |
|  | #24 | double antithrombotic | 45 |
|  | #25 | clopidogrel | 828 |
|  | #26 | prasugrel | 194 |
|  | #27 | ticagrelor | 301 |
|  | #28 | P2Y12 receptor antagonist | 174 |
|  | #29 | #18 OR #19 OR #20 OR #21 OR #22 OR #23 OR #24 OR #25 OR #26 OR #27 OR #28 | 2267 |
|  | #30 | #8 AND #17 AND #29  Update search since Oct 11, 2016 to Oct 1, 2017 | **176**  **7**  **183** |

# Appendix 2

**Details of bleeding definition and compatibility**

We collected major bleeding outcome based on BARC 3-5 criteria because this bleeding definition was standardized including both clinical and laboratory information. BARC definition was the most updated bleeding definition. [1] Furthermore, there were few studies illustrated that BARC 3 was associated with long-term mortality in patients with acute coronary syndrome. [2, 3] We directly collected major bleeding outcome from any trials reported outcome as BARC definition. If not, we considered compatibility criteria. Our criteria was that compatible definition could be standardized based on BARC type 3 – 5 criteria and those definitions must not contained any lower severity of bleeding. For example, TIMI major bleeding definition (fatal bleeding, intracranial hemorrhage, or clinically overt sign of bleeding with reduction of hemoglobin ≥ 5 g/dL) could be categorized into BARC type 3b (reduction of hemoglobin 5 ≥ g/dL), type 3c (intracranial hemorrhage), and type 5 (fatal bleeding). Therefore, TIMI major bleeding was a compatible definition and TIMI major bleeding outcomes could be collected to our analysis. In contrast, a non-official definition, defined as intracranial or gastrointestinal and/or requiring blood transfusion or surgical intervention and/or hospitalization, could not be categorized as a compatible one due to inclusion of hospitalization which was unclear in severity and might not be compatible with BARC 3-5.

## eTable 2 Major bleeding definition of each study and compatibility with BARC 3-5, TIMI major, GUSTO severe, and ISTH major definitions

| **Study Name/ First Author** | **Definition of Major Bleeding** | **Details (in case of non-official definition)** | **Compatibility with BARC**  **3-5** | **Compatibility with TIMI major** | **Compatibility with GUSTO severe** | **Compatibility with ISTH major** |
| --- | --- | --- | --- | --- | --- | --- |
| WOEST^[4]^ | BARC, GUSTO, TIMI | - | Reported as BARC | Reported as TIMI | Reported as GUSTO | BARC 3b,3c,5 |
| PIONEER AF-PCI^[5]^ | BARC, GUSTO, ISTH, TIMI | - | Reported as BARC | Reported as TIMI | Reported as GUSTO | Reported as ISTH |
| REDUAL-PCI^[6]^ | ISTH, TIMI | - | BARC 3a,3b,3c,5 | Reported as TIMI | No *(incompatible due to haemoglobin criteria)* | Reported as ISTH |
| MUSICA registry^[7]^ | PRISM-PLUS | - | BARC 3a,3b,3c | No *(incompatible due to hemoglobin criteria)* | No *(incompatible due to haemoglobin and transfusion criteria)* | Compatible |
| Gao F^[8]^ | TIMI | - | BARC 3b,3c,5 | Reported as TIMI | No *(incompatible due to lab-based criteria of TIMI)* | Compatible |
| WAR-STENT registry^[9]^ | TIMI | - | BARC 3b,3c,5 | Reported as TIMI | No *(incompatible due to lab-based criteria of TIMI)* | Compatible |
| AFCAS registry^[10]^ | BARC 3-5 | - | Reported as BARC | BARC 3b,3c,5 | No *(incompatible due to haemoglobin criteria)* | BARC 3b,3c,5 |
| De Vecchis R^[11]^ | Not defined | - | No *(not defined major bleeding definition)* | No *(not defined major bleeding definition)* | No *(not defined major bleeding definition)* | No *(not defined major bleeding definition)* |
| Sarafoff N^[12]^ | TIMI | - | BARC 3b,3c,5 | Reported as TIMI | No *(incompat due to lab-based criteria of TIMI)* | Compatible |
| Braun OO ^[13]^ | BARC, GUSTO, TIMI | - | Reported as BARC | Reported as TIMI | Reported as GUSTO | BARC 3b,3c,5 |
| Fu A ^[14]^ | BARC, TIMI | - | Reported as BARC | Reported as TIMI | No *(incompatible due to haemoglobin criteria)* | BARC 3b,3c,5 |
| GRACE registry ^[15]^ | x | - | x | x | x | x |
| Suh SY^[16]^ | Non-official definition | Any intracranial bleeding or any bleeding associated with clinically overt signs, and a drop in hemoglobin [2 g/dl which needs transfusion | BARC 3a,3c | No *(incompatible due to haemoglobin criteria)* | No (incompatible due to ignorance of hemodynamic status) | Compatible |
| STENTICO^[17]^ | GUSTO (moderate & severe) | - | BARC 3a,3b,3c | No *(incompatible due to haemoglobin criteria in moderate GUSTO)* | No *(incompatible due to haemoglobin criteria in moderate GUSTO)* | No *(incompatible due to haemoglobin criteria in moderate GUSTO)* |
| REAL registry^[18]^ | Non-official definition | Intracranial or gastrointestinal and/or requiring blood transfusion or surgical intervention and/or hospitalization | No *(incompatible due to inclusion of hospitalization)* | No *(incompatible due to inclusion of hospitalization)* | No *(incompatible due to inclusion of hospitalization)* | No *(incompatible due to inclusion of hospitalization)* |
| Ho KW^[19]^ | Non-official definition | Any bleeding requiring transfusion | 3a | No *(none of the compatible criteria)* | No *(none of the compatible criteria)* | No *(none of the compatible criteria)* |
| Dabrowska M^[20]^ | Non-official definition | Bleeding requiring hospitalization and/or discontinuation of any antithrombotic medication | No | No | No | No |
| Hess CN^[21]^ | Non-official definition | Bleeding requiring hospitalization or intracranial hemorrhage readmission | Compatible (ICH) | Compatible (ICH) | Compatible (ICH) | Compatible (ICH) |
| Kang DO^[22]^ | GUSTO (moderate or severe) | - | BARC 3a,3b,3c | No *(incompatible due to haemoglobin criteria in moderate GUSTO)* | No *(incompatible due to haemoglobin criteria in moderate GUSTO)* | No *(incompatible due to haemoglobin criteria in moderate GUSTO)* |
| Caballero L^[23]^ | PRISM-PLUS | - | BARC 3a,3b,3c | No *(incompatible due to haemoglobin criteria)* | No *(incompatible due to haemoglobin and transfusion criteria)* | Compatible |
| Sambola A^[24]^ | PRISM-PLUS, TIMI | - | BARC 3a,3b,3c | Compatible but could not collect outcomes | No *(incompatible due to haemoglobin and transfusion criteria)* | Compatible |
| Maegdefessel L^[25]^ | Non-official definition | Collected outcomes were severe GI bleeding requiring endoscopy and blood transfusion > 3 units | BARC 3a,3b | No *(incompatible due to bleeding characteristics)* | No *(incompatible due to bleeding characteristics)* | Compatible |
| Sarafoff N^[26]^ | Non-official definition | Intracranial or clinically significant overt signs of haemorrhage associated with >50 g/L decrease in haemoglobin level or an absolute decrease in haematocrit >15% (when haemoglobin level was not available) | BARC 3b,3c | Compatible | No *(incompatible due to lab-based criteria of TIMI)* | Compatible |
| Manzano-Fernandez S^[27]^ | PRISM-PLUS | - | BARC 3a,3b,3c | No *(incompatible due to haemoglobin criteria)* | No *(incompatible due to haemoglobin and transfusion criteria)* | Compatible |
| Ruiz-Nodar JM^[28]^ | PRISM-PLUS | - | BARC 3a,3b,3c | No *(incompatible due to haemoglobin criteria)* | No *(incompatible due to haemoglobin and transfusion criteria)* | Compatible |
| Goto K^[29]^ | GUSTO (moderate or severe) | - | BARC 3a,3b,3c | No *(incompatible due to haemoglobin criteria in moderate GUSTO)* | No *(incompatible due to haemoglobin criteria in moderate GUSTO)* | No *(incompatible due to haemoglobin criteria in moderate GUSTO)* |
| Jang SW^[30]^ | Non-official definition | Decrease in hemoglobin of 2 g/dL or more over a 24-hour period, the need for a transfusion of two or more units of packed red blood cells, bleeding leading to death and bleeding at the following critical sites: intracranial, intraspinal, intraocular, pericardial, intra-articular, retroperitoneal or intramuscular bleeding with com-partment syndrome | BARC 3a,3c,5 | No *(incompatible due to haemoglobin criteria)* | No *(incompatible due to bleeding characteristics)* | Compatible |
| Valencia J^[31]^ | Non-official definition | The appearance of bleeding associated with hemodynamic instability requiring transfusion and/or the performance of a diagnostic or invasive therapeutic procedure, as well as all intracranial bleeding | BARC 3a,3b,3c | No *(incompatible due to bleeding characteristics)* | Compatible | No *(incompatible due to hemoglobin criteria)* |
| ISAR-TRIPLE^[32]^ | BARC, TIMI | - | Reported as BARC | Reported as TIMI | No *(incompatible due to hemoglobin criteria)* | BARC 3b,3c,5 |
| Choi H^[33]^ | TIMI | - | BARC 3b,3c,5 | Reported as TIMI | No *(incompat due to lab-based criteria of TIMI)* | Compatible |

**Abbreviations:** GUSTO, Global Use of Strategies to Open Occluded Coronary Arteries; TIMI, Thrombolysis In Myocardial Infarction; BARC, Bleeding Academic Research Consortium; PRISM-PLUS, Platelet Receptor Inhibition in Ischemic Syndrome Management in Patients Limited by Unstable Signs and Symptoms; GI, gastrointestinal; A+C, aspirin + clopidogrel; A+C+LMWH, aspirin + clopidogrel + low-molecular weight heparin; A+C+VKA, aspirin + clopidogrel + vitamin K antagonist; A+P+VKA, aspirin + prasugrel + vitamin K antagonist; A+T+VKA, aspirin + ticagrelor + vitamin K antagonist; A+VKA, aspirin + vitamin K antagonist; C+VKA, clopidogrel + vitamin K antagonist; T+VKA, ticagrelor + vitamin K antagonist; A+C+r, aspirin + clopidogrel + very low-dose rivaroxaban; C+R, clopidogrel + low-dose rivaroxaban

# Appendix 3

**General characteristics of treatment options**

## eTable 3 General characteristics of treatment options

| **Treatment Abbreviations** | **General characteristics** |
| --- | --- |
| Dual antiplatelet (DAPT) | |
| A+C | Aspirin 81-162 mg OD +  Clopidogrel 75 mg OD |
| Dual therapies | |
| A+VKA | Aspirin 81-162 mg OD +  Vitamin K antagonist (i.e. warfarin, acenocoumarol, phenprocoumon, dosing according to the standard treatment guidelines) |
| C+VKA | Clopidogrel 75 mg OD +  Vitamin K antagonist (i.e. warfarin, acenocoumarol, phenprocoumon, dosing according to the standard treatment guidelines) |
| T+VKA | Ticagrelor 90 mg BID +  Vitamin K antagonist (i.e. warfarin, acenocoumarol, phenprocoumon, dosing according to the standard treatment guidelines) |
| C+R | Clopidogrel 75 mg OD +  Low-dose rivaroxaban (15 mg OD) |
| C+d | Clopidogrel 75 mg OD +  Dabigatran etexilate 110 mg BID |
| C+D | Clopidogrel 75 mg OD +  Dabigatran etexilate 150 mg BID |
| Triple therapies | |
| A+C+VKA | Aspirin 81-162 mg OD +  Clopidogrel 75 mg OD +  Vitamin K antagonist (i.e. warfarin, acenocoumarol, phenprocoumon, dosing according to the standard treatment guidelines) |
| A+P+VKA | Aspirin 81-162 mg OD +  Prasugrel 10 mg OD +  Vitamin K antagonist (i.e. warfarin, acenocoumarol, phenprocoumon, dosing according to the standard treatment guidelines) |
| A+T+VKA | Aspirin 81-162 mg OD +  Ticagrelor 90 mg BID +  Vitamin K antagonist (i.e. warfarin, acenocoumarol, phenprocoumon, dosing according to the standard treatment guidelines) |
| A+C+LMWH | Aspirin 81-162 mg OD +  Clopidogrel 75 mg OD +  Low-molecular weight heparin |
| A+C+r | Aspirin 81-162 mg OD +  Clopidogrel 75 mg OD +  Very low-dose rivaroxaban (2.5 mg BID) |

**Abbreviations:** A, Aspirin; BID, twice daily; C, Clopidogrel; D, Dabigatran; P, Prasugrel; T, Ticagrelor; R, Rivaroxaban (low-dose); r, Rivaroxaban (very low-dose); VKA, Vitamin K antagonist

# Appendix 4

**Description of included RCTs**

## eTable 4.1 Description of included RCTs

| **Study group/ First author** | **Year** | **Location (s)** | **Study design** | **Type of study** | **N** | **Intervention (N)** | **Follow-up period (year)** |
| --- | --- | --- | --- | --- | --- | --- | --- |
| WOEST^[4]^ | 2013 | Neitherland & Belgium | Randomized controlled trials | Multicenter | 563 | A+C+VKA (284)  C+VKA (279) | 1 |
| PIONEER AF-PCI^[5]^ | 2016 | International | Randomized controlled trials | Multicenter | 2124 | A+C+VKA (706)  A+C+r (709)  C+R (709) | 1 |
| REDUAL-PCI^[6]^ | 2017 | International | Randomized controlled trials | Multicenter | 2725 | A+C+VKA (981)  C+d (981)  C+D (763) | 1.17 |

**Abbreviations:** A+C+VKA, aspirin + clopidogrel + vitamin K antagonist; C+VKA, clopidogrel + vitamin K antagonist; A+C+r, aspirin + clopidogrel + very low-dose rivaroxaban; C+R, clopidogrel + low-dose rivaroxaban, C+d; clopidogrel + low-dose dabigatran, C+D; clopidogrel + high-dose dabigatran

## eTable 4.2 Description of participants of included RCTs

| **Study group/ First author** | **Regimen** | **Age (year)** | | **Male (%)** | **DM (%)** | **HTN (%)** | **DLP (%)** | **Smoking history (%)** | **Previous MI (%)** | **Previous PCI (%)** | **CKD (%)** | **Bleeding history (%)** | **Ejection Fraction (%)** | |
| --- | --- | --- | --- | --- | --- | --- | --- | --- | --- | --- | --- | --- | --- | --- |
| WOEST^[4]^ | A+C+VKA | 69.5 ± 8 | Mean | 82 | 25 | 68 | 72 | 15 | 35 | 36 | 17 | 5 | 47 ± 13 | Mean |
|  | C+VKA | 70.3 ± 7 | Mean | 77 | 24 | 69 | 68 | 22 | 34 | 31 | 18 | 5 | 46 ± 15 | Mean |
| PIONEER AF-PCI^[5]^ | A+C+VKA | 69.9 ± 8.7 | Mean | 73.4 | 31.3 | 75.4 | 44.8 | 6.8 | 22.2 | NA | NA | 0.7 | NA | - |
|  | A+C+r | 70 ± 9.1 | Mean | 75.5 | 28.1 | 73.2 | 41.6 | 7.9 | 25.4 | NA | NA | 1.3 | NA | - |
|  | C+R | 70.4 ± 9.1 | Mean | 74.5 | 28.8 | 73.3 | 42.6 | 5.2 | 19.8 | NA | NA | 1 | NA | - |
| REDUAL-PCI^[6]^ | A+C+VKA | 71.7 ± 8.9 | Mean | 76.5 | 37.9 | NA | NA | NA | 27.3 | 35.4 | 19.2 | NA | NA | - |
|  | C+d | 71.5 ± 8.9 | Mean | 74.2 | 36.9 | NA | NA | NA | 24.2 | 33.2 | 16 | NA | NA | - |
|  | C+D | 68.6 ± 7.7 | Mean | 77.6 | 34.1 | NA | NA | NA | 25.4 | 31.3 | 15.2 | NA | NA | - |

**Abbreviations:** NA, Not available; DM, Diabetes Mellitus; HTN, Hypertension; DLP, Dyslipidemia; CKD, Chronic Kidney Disease; MI, Myocardial infarction; PCI, Percutaneous coronary intervention; IQR, Interquartile range; A+C+VKA, aspirin + clopidogrel + vitamin K antagonist; C+VKA, clopidogrel + vitamin K antagonist; A+C+r, aspirin + clopidogrel + very low-dose rivaroxaban; C+R, clopidogrel + low-dose rivaroxaban, C+d; clopidogrel + low-dose dabigatran, C+D; clopidogrel + high-dose dabigatran

## eTable 4.3 Procedural Characteristics (RCTs)

| **Study group/ First author** | **Regimen** | **Type of index event/**  **indication of PCI** | | **Number of vessel treated (N)** | | **Number of stent implanted (N)** | | **Type of stent implanted** | |
| --- | --- | --- | --- | --- | --- | --- | --- | --- | --- |
|  |  | **ACS (%)** | **SCAD (%)** |  |  |  |  | **BMS (%)** | **DES (%)** |
| WOEST^[4]^ | A+C+VKA | 30 | 70 | 1 = 70%,  2 = 24%,  3 = 5% | Percent | NA | - | 30 | 68 |
|  | C+VKA | 25 | 75 | 1 = 72%,  2 = 20%,  3 = 5% | Percent | NA | - | 32 | 66 |
| PIONEER AF-PCI^[5]^ | A+C+VKA | 36.4 | 63.6 | NA | - | NA | - | 31.8 | 68.2 |
|  | A+C+r | 39.4 | 60.6 | NA | - | NA | - | 31.2 | 68.8 |
|  | C+R | 39.6 | 60.4 | NA | - | NA | - | 32.6 | 67.4 |
| REDUAL-PCI^[6]^ | A+C+VKA | 48.4 | 43.7 | NA | - | NA | - | 13.6 | 84.6 |
|  | C+d | 51.9 | 44.1 | NA | - | NA | - | 15.1 | 82.1 |
|  | C+D | 51.2 | 41.9 | NA | - | NA | - | 16.1 | 81.5 |

**Abbreviations:** NA, Not available; PCI, percutaneous coronary intervention; ACS, acute coronary syndrome; SCAD, stable coronary artery disease; BMS, bare-metal stent; DES, drug-eluting stent; IQR, Interquartile range; A+C+VKA, aspirin + clopidogrel + vitamin K antagonist; C+VKA, clopidogrel + vitamin K antagonist; A+C+r, aspirin + clopidogrel + very low-dose rivaroxaban; C+R, clopidogrel + low-dose rivaroxaban, C+d; clopidogrel + low-dose dabigatran, C+D; clopidogrel + high-dose dabigatran

## eTable 4.4 Indication of anticoagulant therapy & Risk scores (CHADS_2_ score & HASBLED score) among RCTs

| **Study group/ First author** | **Regimen** | **Indication of anticoagulant** | | | | | **Thromboembolic risk**  **(patients with AF only)** | | | **Bleeding risk**  **(patients with AF only)** |
| --- | --- | --- | --- | --- | --- | --- | --- | --- | --- | --- |
|  |  | **AF (%)** | **MHV (%)** | **LV**  **thrombus (%)** | **ACS/ dyskinesia (%)** | **VTE (%)** | **Average CHADS_2_ score** | | **CHADS_2_ ≥ 2**  **(%)** | **Average HASBLED score** |
| WOEST^[4]^ | A+C+VKA | 69 | 11 | NA | NA | NA | NA | - | 86 | NA |
|  | C+VKA | 69 | 10 | NA | NA | NA | NA | - | 88 | NA |
| PIONEER AF-PCI^[5]^ | A+C+VKA | 100 | 0 | 0 | 0 | 0 | NA | - | 56.2 | NA |
|  | A+C+r | 100 | 0 | 0 | 0 | 0 | NA | - | 54.5 | NA |
|  | C+R | 100 | 0 | 0 | 0 | 0 | NA | - | 55.1 | NA |
| REDUAL-PCI^[6]^ | A+C+VKA | 100 | 0 | 0 | 0 | 0 | 3.8* | 1.5 | 80.3 | 2.8 ± 0.8 |
|  | C+d | 100 | 0 | 0 | 0 | 0 | 3.7* | 1.5 | 76.6 | 2.7 ± 0.7 |
|  | C+D | 100 | 0 | 0 | 0 | 0 | 3.3* | 1.6 | 67.6 | 2.6 ± 0.7 |

**Abbreviations:** NA, Not available; AF, Atrial fibrillation; MHV, mechanical heart valve; LV, Left ventricular; ACS, Acute coronary syndrome; VTE, Venous thromboembolism; A+C+VKA, aspirin + clopidogrel + vitamin K antagonist; C+VKA, clopidogrel + vitamin K antagonist; A+C+r, aspirin + clopidogrel + very low-dose rivaroxaban; C+R, clopidogrel + low-dose rivaroxaban, C+d; clopidogrel + low-dose dabigatran, C+D; clopidogrel + high-dose dabigatran

***** CHA_2_DS_2_-VASc score

## eTable 4.5 Detail of regimens used in each study of RCTs

| **Study name/ First author**  **(year)** | **Regimen** | **Type of OAC** | **INR target** | **TTR**  **(%)** | **VKA**  **Naïve (%)** | **Dose of ASA**  **(mg)** | **Dose of P2Y_12_ inhibitor (mg)** | **Specified period of regimen use** | **Duration of regimen used/ percent continuation at the point of outcome measurement** |
| --- | --- | --- | --- | --- | --- | --- | --- | --- | --- |
| WOEST^[4]^ | A+C+VKA | NA | NA  (target INR being that indicated for underlying) | NA | NA | 80-100 OD | 75 OD | Antiplatelets: 1-12 months for BMS in SCAD, 12 months in ACS or DES | OAC at 1 year = 91·2%  Clopidogrel at 1 year = 78·9%  Aspirin at 1 year = 66·5% |
|  | C+VKA | NA | NA (target INR being that indicated for underlying) | NA | NA | 80-100 OD | 75 OD | Antipletelts: 1-12 months for BMS in SCAD, 12 months in ACS or DES | OAC at 1 year = 92·5%  Clopidogrel at 1 year = 80·6% |
| PIONEER AF- PCI^[5]^ | A+C+VKA | Warfarin | 2.0 – 3.0 | 65 | NA | 75 – 100 OD | 75 OD | 1 month, 6 months, and 12 months stratum | - |
|  | C+R | Low-dose rivaroxaban | - | - | - | - | 75 OD | 12 months | - |
|  | A+C+r | Very low-dose rivaroxaban | - | - | - | 75-100 OD | 75 OD | 1 month, 6 months, and 12 months stratum | - |
| REDUAL-PCI^[6]^ | A+C+VKA | Warfarin | 2.0 - 3.0 | 64 | NA | ≤ 100 OD | Clopidogrel 75 OD, ticagrelor 90 BID | Triple therapy: aspirin discontinuation at 1 month after BMS implantation or 3 months after DES implantation  Dual therapy: no specified | Concomitant antiplatelet at 105 days = 92.7%  Concomitant antiplatelet at 1 year = 56.4% |
|  | C+d** | Dabigatran 110 mg BID | - | - | - | - | Clopidogrel 75 OD, ticagrelor 90 BID | NA | Concomitant antiplatelet at 105 days = 96.5%  Concomitant antiplatelet at 1 year = 57.8% |
|  | C+D** | Dabigatran 150 mg BID | - | - | - | - | Clopidogrel 75 OD, ticagrelor 90 BID | NA | Concomitant antiplatelet at 105 days = 96.9%  Concomitant antiplatelet at 1 year = 59.8% |

**Abbreviation:** OAC, Oral anticoagulant; TTR, Time in therapeutic range; VKA, Vitamin K antagonist; ASA, Aspirin; INR, International normalized ratio; NA, Not available; ACS, Acute coronary syndrome; SCAD, Stable coronary artery disease; BMS, Bare-metal stent; DES, Drug-eluting stent; AF, Atrial fibrillation; MHV, Mechanical heart valve; A+C+VKA, aspirin + clopidogrel + vitamin K antagonist; C+VKA, clopidogrel + vitamin K antagonist; A+C+r, aspirin + clopidogrel + very low-dose rivaroxaban; C+R, clopidogrel + low-dose rivaroxaban

*Median (Range)

**rate of clopidogrel use = 88.0%, rate of ticagrelor use = 12.0%

## eTable 4.6 Summarized risk of bias of included RCTs

| **Study Name/**  **First Author**  **(year)** | **Randomization** | **Deviation from intended intervention** | **Missing outcome data** | **Measurement of outcomes** | **Selection of the reported results** | **Overall** |
| --- | --- | --- | --- | --- | --- | --- |
| WOEST^[4]^ | Low | Some Concerns | Low | Low | Low | **Some Concerns** |
| PIONEER AF-PCI^[5]^ | Low | Some Concerns | Low | Low | Low | **Some Concerns** |
| REDUAL-PCI^[6]^ | Low | Some Concerns | Low | Low | Low | **Some Concerns** |

# Appendix 5

**Description of included non-RCTs**

## eTable 5.1 Description of included non-RCTs

| **Study group/ First author** | **Year** | **Location (s)** | **Study design** | **Type of study** | **N** | **Intervention (N)** | **Follow-up period (year)** | **Methods of confounding factors adjustment** |
| --- | --- | --- | --- | --- | --- | --- | --- | --- |
| MUSICA registry^[7]^ | 2009 | Spain & United Kingdom | Prospective cohort | Multicenter | 405 | A+C+VKA (248)  A+C+LMWH (30)  A+VKA (6)  C+VKA (37)  A+LMWH (1)  C+LMWH (2)  A+C (81) | 0.5 | - |
| Gao F^[8]^ | 2010 | China | Prospective cohort | Single center | 622 | A+C+VKA (142)  A+VKA (16)  C+VKA (109)  A+C (355) | 1 | - |
| WAR-STENT registry^[9]^ | 2014 | Italy | Prospective cohort | Multicenter | 401 | A+C+VKA (339)  A+VKA/C+VKA (20)  A+C (42) | 1 | Multivariate analysis |
| AFCAS registry^[10]^ | 2014 | 5 European countries | Prospective cohort | Multicenter | 914 | A+C+VKA (679)  C+VKA (73)  A+C (162) | 1 | Multinomial logistic regression |
| De Vecchis R^[11]^ | 2016 | Italy | Retrospective cohort | Single center | 98 | A+C+VKA (48)  A+VKA/C+VKA (31)  A+C (19) | 1 | - |
| Sarafoff N^[12]^ | 2013 | Germany | Prospective cohort | Multicenter | 377 | A+C+VKA (356)  A+P+VKA (21) | 0.5 | Multivariate analysis |
| Braun OO^[13]^ | 2015 | Sweden | Retrospective cohort with historical control | Multicenter | 266 | A+C+VKA (159)  T+VKA (107) | 0.25 | - |
| Fu A^[14]^ | 2016 | NA | Retrospective analysis with prospective follow-up | NA | 152 | A+C+VKA (125)  A+T+VKA (27) | 1 | Multivariate analysis |
| GRACE registry^[15]^ | 2007 | North and South America, Europe, Australia, and New Zealand | Retrospective cohort | Multicenter | 800 | A+C+VKA (580)  A+VKA (107)  C+VKA (113) | 0.5 | - |
| Suh SY^[16]^ | 2014 | Korea | Retrospective cohort | Single center | 203 | A+C+VKA (37)  A+C (166) | 3.5 | - |
| STENTICO^[17]^ | 2009 | France | Prospective cohort | Multicenter | 359 | A+C+VKA (125)  A+C (234) | 1 | - |
| REAL registry^[18]^ | 2012 | Italy | Prospective cohort | Multicenter | 622 | A+C+VKA (111)  A+VKA (205)  A+C (306) | 1 | Multivariate analysis |
| Ho KW^[19]^ | 2013 | Canada | Retrospective cohort | Single center | 602 | A+C+VKA (382)  A+C (220) | 0.49 | Multivariate analysis |
| Dabrowska M^[20]^ | 2013 | Poland | Prospective cohort | Single center | 47 | A+C+VKA (18)  A+C (29) | 1 | - |
| Hess CN^[21]^ | 2015 | United States of America | Retrospective cohort | Multicenter | 4959 | A+C+VKA (1370)  A+C (3589) | 2 | Inverse probability weighted propensity score |
| Kang DO^[22]^ | 2015 | Korea | Retrospective cohort | Multicenter | 367 | A+C+VKA (131)  A+C (236) | 1.72 | Propensity score matching |
| Caballero L^[23]^ | 2013 | NA | Retrospective cohort | Multicenter | 81 | A+C+VKA (45)  A+C (36) | 1.42 | Multivariable analysis |
| Sambola A^[24]^ | 2016 | Spain | Prospective cohort | Multicenter | 585 | A+C+VKA (319)  A+C (266) | 1 | Multivariate analysis |
| Maegdefessel L^[25]^ | 2008 | Germany | Retrospective cohort | Single center | 159 | A+C+VKA (14)  A+C+LMWH (42)  A+C (103) | 1.4 | - |
| Sarafoff N^[26]^ | 2008 | Germany | Prospective cohort | Single center | 515 | A+C+VKA (306)  A+C (209) | 2 | - |
| Manzano-Fernandez S^[27]^ | 2008 | Spain | Retrospective cohort | Single center | 104 | A+C+VKA (51)  A+C (53) | 1 (median) | Multivariate analysis |
| Ruiz-Nodar JM^[28]^ | 2008 | Spain | Retrospective cohort | Multicenter | 426 | A+C+VKA (242)  A+C (184) | 595 days (median) | Multivariate analysis |
| Goto K^[29]^ | 2014 | Japan | Retrospective cohort | Multicenter | 1007 | OAC-based regimen*  No OAC-based regimen* | 5.1 (median) | Multivariate analysis |
| Jang SW^[30]^ | 2011 | Korea | Retrospective cohort | Multicenter | 362 | OAC-based regimen*  No OAC-based regimen* | NA | Multivariate analysis |
| Valencia J^[31]^ | 2008 | NA | Prospective cohort | NA | 70 | A+C+VKA (45)  A+VKA (2)  C+VKA (5)  A+C (18) | 1 | - |
| ISAR-TRIPLE^[32]^ | 2015 | Germany & Denmark | Post-hoc analysis of randomized controlled trials | Multicenter | 614 | A+C+VKA (307)  A+VKA (307) | 0.75 | - |
| Choi H^[33]^ | 2017 | Korea | Prospective cohort | Single center | 704 | A+C+VKA (75)  A+C (629) | 6.2 | Inverse probability of treatment weighting |

*Unclear of definite regimens used

**Abbreviations:** NA, Not available; A+C, aspirin + clopidogrel; A+C+LMWH, aspirin + clopidogrel + low-molecular weight heparin; A+C+VKA, aspirin + clopidogrel + vitamin K antagonist; A+P+VKA, aspirin + prasugrel + vitamin K antagonist; A+T+VKA, aspirin + ticagrelor + vitamin K antagonist; A+VKA, aspirin + vitamin K antagonist; C+VKA, clopidogrel + vitamin K antagonist; T+VKA, ticagrelor + vitamin K antagonist

## eTable 5.2 Description of participants of included non-RCTs

| **Study group/ First author** | **Regimen** | **Age (year)** | | **Male (%)** | **DM (%)** | **HTN (%)** | **DLP (%)** | **Smoking history (%)** | **Previous MI (%)** | **Previous PCI (%)** | **CKD (%)** | **Bleeding history (%)** | **Ejection Fraction (%)** | |
| --- | --- | --- | --- | --- | --- | --- | --- | --- | --- | --- | --- | --- | --- | --- |
| MUSICA registry^[7]^ | A+C+VKA | 70 ± 9 | Mean | 82 | 36 | 66.2 | 48.6 | 41.4 | 38.5 | 45.7 | 6.5 | NA | 53.5 ± 14.4 | Mean |
|  | A+VKA/C+VKA | 72 ± 9 | Mean | 78 | 26.1 | 47.8 | 50 | 45.6 | 28.3 | 26.1 | 2.2 | NA | 49.8 ± 12.5 | Mean |
|  | A+C | 72 ± 8 | Mean | 80 | 25.9 | 67.9 | 45.7 | 42 | 25.9 | 24.7 | 6.2 | NA | 55.5 ± 14.4 | Mean |
| Gao F^[8]^ | A+C+VKA | 70.97 ± 5.56 | Mean | 72.2 | 38.3 | 73 | 71.3 | 33.9 | 19.1 | 12.1 | 23.2 | NA | NA | - |
|  | A+VKA/C+VKA | 72.81 ± 5.22 | Mean | 69 | 40.2 | 69 | 66.7 | 28.7 | 21.8 | 15 | 21.7 | NA | NA | - |
|  | A+C | 71.7 ± 5.4 | Mean | 71.2 | 35.7 | 68 | 67.4 | 36.9 | 17 | 13 | 28 | NA | NA | - |
| WAR-STENT registry^[9]^ | A+C+VKA | 74 ± 9 | Mean | 26 | 36 | 84 | 51 | 12 | 25 | 34 | 27 | 3 | 47 ± 11 | Mean |
|  | A+VKA/C+VKA | 71 ± 11 | Mean | 25 | 35 | 85 | 55 | 15 | 15 | 35 | 30 | 0 | 48 ± 10 | Mean |
|  | A+C | 76 ± 8 | Mean | 26 | 31 | 81 | 67 | 24 | 17 | 26 | 40 | 0 | 46 ± 12 | Mean |
| AFCAS registry^[10]^ | A+C+VKA | 73 ± 8 | Mean | 71 | 37 | 84 | 67 | 10 | 25 | 16 | NA | 4 | 49 ± 14 | Mean |
|  | C+VKA | 74 ± 8 | Mean | 71 | 37 | 82 | 63 | 10 | 22 | 22 | NA | 4 | 48 ± 14 | Mean |
|  | A+C | 73 ± 8 | Mean | 65 | 33 | 88 | 67 | 9 | 28 | 22 | NA | 6 | 52 ± 14 | Mean |
| De Vecchis R^[11]^ | A+C+VKA | 72 ± 7.5 | Mean | 46 | 37.5 | 75 | 56 | 12.5 | 25 | 35 | 27 | 0 | 50.7 ± 4.7 | Mean |
|  | A+VKA/C+VKA | 73 ± 8 | Mean | 39 | 32 | 87 | 57 | 19.3 | 19.3 | 32 | 26 | 2 | 49.8 ± 6 | Mean |
|  | A+C | 77 ± 5 | Mean | 53 | 31.5 | 68 | 47 | 21 | 31.5 | 37 | 37 | 2 | 50 ± 5 | Mean |
| Sarafoff N^[12]^ | A+C+VKA | 72.6 ± 7 | Mean | 80.3 | 29.2 | 58.7 | 69.6 | 9.3 | 30.6 | NA | NA | NA | 46 ± 15 | Mean |
|  | A+P+VKA | 71 ± 11 | Mean | 76.2 | 33.3 | 57.1 | 76.2 | 4.8 | 19 | NA | NA | NA | 41 ± 13 | Mean |
| Braun OO^[13]^ | A+C+VKA | 67 ± 11 | Mean | 78 | 18.2 | 42.1 | NA | NA | NA | NA | 1.9 | NA | NA | - |
|  | T+VKA | 74 ± 9 | Mean | 75.7 | 34.6 | 74.8 | NA | NA | NA | NA | 3.7 | NA | NA | - |
| Fu A ^[14]^ | A+C+VKA | 69.3 ± 13.9 | Mean | 72 | 37.6 | 76 | 69.6 | 29.6 | 28 | 25.6 | 5.6 | 6.4 | NA | - |
|  | A+T+VKA | 56.4 ± 10.7 | Mean | 71.4 | 18.5 | 51.9 | 59.3 | 40.7 | 18.5 | 11.1 | 3.7 | 11.1 | NA | - |
| GRACE registry ^[15]^ | A+C+VKA | 55-75 (64) | Median (IQR) | 74 | 23 | 57 | 52 | 58 | 27 | 19 | NA | NA | NA | - |
|  | A+VKA | 66 (58-77) | Median (IQR) | 70 | 23 | 59 | 47 | 53 | 26 | 16 | NA | NA | NA | - |
|  | C+VKA | 66 (58-77) | Median (IQR) | 70 | 23 | 59 | 47 | 53 | 26 | 16 | NA | NA | NA | - |
| Suh SY^[16]^ | A+C+VKA | 65.6 ± 10.6 | Mean | 75.7 | 24.3 | 67.6 | 16.2 | 13.5 | 2.7 | 10.8 | 2.7 | NA | 55 ± 14.5 | Mean |
|  | A+C | 68.9 ± 9.9 | Mean | 59.6 | 38 | 71.1 | 24.1 | 12.7 | 4.2 | 15.1 | 10.8 | NA | 51.1 ± 15.5 | Mean |
| STENTICO^[17]^ | A+C+VKA | 71 ± 9 | Mean | 83 | 26 | 59 | 60 | 46 | 26 | 22 | NA | NA | NA | - |
|  | A+C | 72 ± 9 | Mean | 84 | 30 | 65 | 57 | 52 | 25 | 26 | NA | NA | NA | - |
| REAL registry^[18]^ | A+C+VKA  A+VKA  A+C | 73.1 ± 8.4 | Mean | 73 | 31 | 88 | 59 | 11 | 37 | 11 | 9 | NA | NA | - |
|  | *data in each group was not available | | | | | | | | | | | | | |
| Ho KW^[19]^ | A+C+VKA | 72.9 ± 9.5 | Mean | 74.3 | 36.9 | 76.4 | 75.9 | 59.2 | NA | NA | 3.4 | NA | 40 ± 15.6 | Mean |
|  | A+C | 70.5 ± 10.7 | Mean | 65.9 | 32.3 | 82.3 | 75.5 | 56.4 | NA | NA | 4.1 | NA | 45.5 ± 14.8 | Mean |
| Dabrowska M^[20]^ | A+C+VKA | 69 ± 7 | Mean | 66 | 57 | 93 | 98 | NA | 43 | 43 | NA | NA | 45 ± 11 | Mean |
|  | A+C | 71 ± 9 | Mean | 53 | 28 | 85 | 95 | NA | 40 | 35 | NA | NA | 50 ± 12 | Mean |
| Hess CN^[21]^ | A+C+VKA | 77 (72-82) | Median (IQR) | 63.1 | 35.5 | 83.6 | 67.3 | NA | 31.5 | 31 | NA | 3 | NA | - |
|  | A+C | 78 (72.84) | Median (IQR) | 55.4 | 30 | 81.1 | 62 | NA | 27.6 | 28.3 | NA | 3.5 | NA | - |
| Kang DO^[22]^ | A+C+VKA | 69.06 ± 8.2 | Mean | 66.4 | 32.8 | 74 | 39.6 | 39.6 | 6.8 | NA | 9.1 | 2.2 | NA | - |
|  | A+C | 67.52 ± 9.73 | Mean | 64.4 | 30.5 | 75 | 46.1 | 38.9 | 8.4 | NA | 10.1 | 0.8 | NA | - |
| Caballero L^[23]^ | A+C+VKA  A+C | 83.1 ± 2.9 | Mean | 57.9 | 32.6 | 77.4 | NA | NA | 35.4 | NA | 26.9 | NA | NA | - |
|  | *data of each group was not available | | | | | | | | | | | | | |
| Sambola A^[24]^ | A+C+VKA | 73 ± 8 | Mean | 74.9 | 40.4 | 79.9 | 54.4 | 45.8 | 36.5 | 40.3 | 15.1 | NA | NA | - |
|  | A+C | 73 ± 8 | Mean | 76.3 | 34.2 | 69.5 | 45.4 | 55.2 | 30.2 | 23.6 | 17.4 | NA | NA | - |
| Maegdefessel L^[25]^ | A+C+VKA | 68.5 ± 10.6 | Mean | 78.6 | 7.1 | 78.6 | 64.3 | 28.6 | NA | NA | NA | NA | 47.7 ± 19 | Mean |
|  | A+C+LMWH | 72.1 ± 8.5 | Mean | 66.7 | 33.3 | 78.6 | 54.8 | 19 | NA | NA | NA | NA | 50 ± 12.7 | Mean |
|  | A+C | 69.8 ± 9.2 | Mean | 73.5 | 30.1 | 91.3 | 68 | 15.5 | NA | NA | NA | NA | 55.5 ± 14.1 | Mean |
| Sarafoff N^[26]^ | A+C+VKA | 71.4 ± 9.9 | Mean | 75 | 26 | 88 | 75 | 10 | 32 | 42 | NA | NA | 47.3 ± 14.6 | Mean |
|  | A+C | 72.4 ± 9.3 | Mean | 75 | 28 | 90 | 61 | 10 | 32 | 37 | NA | NA | 48.9 ± 14.5 | Mean |
| Manzano-Fernandez S^[27]^ | A+C+VKA | 69 ± 4 | Mean | 74 | 51 | 86 | 57 | 27 | 27 | 64 | 57 | 14 | 50 (42-60) | Median (IQR) |
|  | A+C | 74 ± 8 | Mean | 66 | 54 | 77 | 52 | 15 | 29 | 68 | 60 | 13 | 55 (43-55) | Median (IQR) |
| Ruiz-Nodar JM^[28]^ | A+C+VKA | 71.6 ± 8.7 | Mean | 70.7 | 42.5 | 81.6 | NA | NA | NA | NA | 10.9 | NA | NA | - |
|  | A+C | 71.2 ± 8.5 | Mean | 70.4 | 41.8 | 72.1 | NA | NA | NA | NA | 22.9 | NA | NA | - |
| Goto K^[29]^ | A+C+VKA | 72 ± 8.8 | Mean | 75.7 | 35 | 86 | NA | 23.3 | 12.3 | NA | 5.5 | NA | 54.4 ± 14.4 | Mean |
|  | A+C | 73 ± 9.7 | Mean | 67 | 33.6 | 84.8 | NA | 21.6 | 12 | NA | 5.6 | NA | 56.4 ± 13.8 | Mean |
| Jang SW^[30]^ | A+C+VKA | 66 ± 7.2 | Mean | 67.9 | 42.9 | 60.7 | NA | NA | NA | NA | NA | NA | 52.5 ± 9.7 | Mean |
|  | A+C | 69 ± 7.9 | Mean | 68 | 35.6 | 59 | NA | NA | NA | NA | NA | NA | 53.5 ± 11.3 | Mean |
| Valencia J^[31]^ | A+C+VKA  A+C | 70.5 ± 8.7 | Mean | 81.4 | 37.2 | 67.1 | 55.7 | 14.3 | NA | NA | 7.1 | NA | NA | - |
|  | *data of each group was not available | | | | | | | | | | | | | |
| ISAR-TRIPLE^[32]^ | A+C+VKA | 73.3 ± 8.7 | Mean | 78.8 | 23.5 | 75.6 | 74.9 | 10.4 | 24.8 | NA | NA | NA | NA | - |
|  | A+VKA | 73.9 ± 7.7 | Mean | 74.6 | 27.7 | 76.9 | 73.9 | 9.1 | 29.3 | NA | NA | NA | NA | - |
| Choi H^[33]^ | A+C+VKA | 68 (60-73) | Median (IQR) | 73.3 | 30.7 | 66.7 | 41.3 | NA | 9.3 | 10.7 | 8 | NA | 56.5 (48-62) | Median (IQR) |
|  | A+C | 68 (61-74) | Median (IQR) | 73.9 | 34.7 | 65 | 41.8 | NA | 14.5 | 19.1 | 7.2 | NA | 60 (52-64) | Median (IQR) |

**Abbreviations:** NA, Not available; DM, Diabetes Mellitus; HTN, Hypertension; DLP, Dyslipidemia; CKD, Chronic Kidney Disease; MI, Myocardial infarction; PCI, Percutaneous coronary intervention; IQR, Interquartile range; A+C, aspirin + clopidogrel; A+C+LMWH, aspirin + clopidogrel + low-molecular weight heparin; A+C+VKA, aspirin + clopidogrel + vitamin K antagonist; A+P+VKA, aspirin + prasugrel + vitamin K antagonist; A+T+VKA, aspirin + ticagrelor + vitamin K antagonist; A+VKA, aspirin + vitamin K antagonist; C+VKA, clopidogrel + vitamin K antagonist; T+VKA, ticagrelor + vitamin K antagonist

## eTable 5.3 Procedural Characteristics (non-RCTs)

| **Study group/ First author** | **Regimen** | **Type of index event/**  **indication of PCI** | | **Number of vessel treated (N)** | | **Number of stent implanted (N)** | | **Type of stent implanted** | |
| --- | --- | --- | --- | --- | --- | --- | --- | --- | --- |
|  |  | **ACS (%)** | **SCAD (%)** |  |  |  |  | **BMS (%)** | **DES (%)** |
| MUSICA registry^[7]^ | A+C+VKA | 69.8 | 30.2 | 1.9 ± 0.79 | Mean | 1.47 ± 0.73 | Mean | 51.8 | 48.2 |
|  | A+VKA/C+VKA | 69.6 | 30.4 | 1.8 ± 0.8 | Mean | 1.54 ± 0.78 | Mean | 71.7 | 28.3 |
|  | A+C | 75.3 | 24.7 | 1.51 ± 0.66 | Mean | 1.47 ± 0.87 | Mean | 50.6 | 49.4 |
| Gao F^[8]^ | A+C+VKA | 12.2 | 87.8 | NA | - | 1.97 ± 0.91 | Mean | 0 | 100 |
|  | A+VKA/C+VKA | 13.8 | 86.2 | NA | - | 2.28 ± 1.09 | Mean | 0 | 100 |
|  | A+C | 15.3 | 84.7 | NA | - | 2.1 ± 0.99 | Mean | 0 | 100 |
| WAR-STENT registry^[9]^ | A+C+VKA | 66 | 29 | NA | - | NA | - | 63 | 34 |
|  | A+VKA/C+VKA | 55 | 40 | NA | - | NA | - | 60 | 20 |
|  | A+C | 65 | 24 | NA | - | NA | - | 67 | 33 |
| AFCAS registry^[10]^ | A+C+VKA | 55 | 46 | 1 = 85%,  2 = 13%,  3 = 1% | Percent | NA | - | 76 | 24 |
|  | C+VKA | 57 | 43 | 1 = 82%,  2 = 18%,  3 = 0% | Percent | NA | - | 79 | 21 |
|  | A+C | 66 | 34 | 1 = 80%,  2 = 18%,  3 = 1% | Percent | NA | - | 68 | 32 |
| De Vecchis R^[11]^ | A+C+VKA | 69 | 25 | NA | - | NA | - | NA | NA |
|  | A+VKA/C+VKA | 61.3 | 38.7 | NA | - | NA | - | NA | NA |
|  | A+C | 68.4 | 31.6 | NA | - | NA | - | NA | NA |
| Sarafoff N^[12]^ | A+C+VKA | 35.1 | 64.9 | NA | - | NA | - | 0 | 100 |
|  | A+P+VKA | 66.7 | 33.3 | NA | - | NA | - | 0 | 100 |
| Braun OO ^[13]^ | A+C+VKA | 100 | 0 | NA | - | NA | - | 78.6 | 21.4 |
|  | T+VKA | 100 | 0 | NA | - | NA | - | 25.2 | 74.8 |
| Fu A ^[14]^ | A+C+VKA | 74.4 | 25.6 | NA | - | 0 = 1.6%,  1 = 59.2%, 2 = 24%,  3 = 10.4%, ≥4 = 4.8% | Percent | 46.4 | 52 |
|  | A+T+VKA | 96.3 | 3.7 | NA | NA | 0 = 3.7%,  1 = 55.6%, 2 = 22.2%, 3 = 11.1%, ≥ 4 = 7.4% | Percent | 25.9 | 70.4 |
| GRACE registry^[15]^ | A+C+VKA | 100 | 0 | NA | - | NA | - | 72 | 28 |
|  | A+VKA | 100 | 0 | NA | - | NA | - | 78 | 22 |
|  | C+VKA | 100 | 0 | NA | - | NA | - | 78 | 22 |
| Suh SY^[16]^ | A+C+VKA | 32.4 | 67.6 | 1 = 83.8%,  ≥ 2 =16.2% | Percent | 1.14 ± 0.49 | Mean | 18.9 | 81.1 |
|  | A+C | 42.1 | 57.8 | 1 = 78.9%,  ≥ 2 = 21.1% | Percent | 1.19 ± 0.54 | Mean | 16.9 | 83.1 |
| STENTICO^[17]^ | A+C+VKA | 76 | 27 | 1.28 ± 0.59 | Mean | 1.46 ± 0.86 | Mean | 75 | 25 |
|  | A+C | 75 | 25 | 1.37 ± 0.62 | Mean | 1.55 ± 0.83 | Mean | 67 | 33 |
| REAL registry^[18]^ | A+C+VKA  A+VKA  A+C | 63 | 100-63 | 1 = 80%,  ≥ 2 = 20% | percent | NA | - | 71 | 25 |
|  | *data of each group was not available | | | | | | | | |
| Ho KW^[19]^ | A+C+VKA | 70.7 | 29.3 | 1.8 ± 1.1 | Mean | 2 ± 1.2 | Mean | reported as mean | reported as mean |
|  | A+C | 67.7 | 32.3 | 1.8 ± 1.1 | Mean | 2.1 ± 1.3 | Mean | reported as mean | reported as mean |
| Dabrowska M^[20]^ | A+C+VKA | NA | NA | NA | - | NA | - | 73 | 27 |
|  | A+C | NA | NA | NA | - | NA | - | 78 | 22 |
| Hess CN^[21]^ | A+C+VKA | 100 | 0 | NA | - | NA | - | 52 | 48 |
|  | A+C | 100 | 0 | NA | - | NA | - | 47.8 | 52.2 |
| Kang DO^[22]^ | A+C+VKA | 77.8 | 18.3 | 2.05 ± 0.96 | Mean | 1.52 ± 0.77 | Mean | 0 | 100 |
|  | A+C | 77.4 | 19.4 | 2.17 ± 1.04 | Mean | 1.63 ± 0.88 | Mean | 0 | 100 |
| Caballero L^[23]^ | A+C+VKA | data in each group not available | data in each group not available | NA | - | NA | - | data in each group not available | data in each group not available |
|  | A+C | data in each group not available | data in each group not available | NA | - | NA | - | data in each group not available | data in each group not available |
| Sambola A^[24]^ | A+C+VKA | 68.3 | 31.7 | NA | - | NA | - | 60.7 | 39.3 |
|  | A+C | 79.2 | 20.8 | NA | - | NA | - | 58.8 | 41.2 |
| Maegdefessel L^[25]^ | A+C+VKA | 71.4 | 28.6 | 1 = 28.6%,  2 = 35.7%,  3 = 35.7% | Percent | NA | - | NA | NA |
|  | A+C+LMWH | 85.7 | 28.6 | 1 = 28.6%,  2 = 33.3%,  3 = 38.1% | Percent | NA | - | NA | NA |
|  | A+C | 88.4 | 33 | 1 = 33%,  2 = 26.2%,  3 = 40.8% | Percent | NA | - | NA | NA |
| Sarafoff N^[26]^ | A+C+VKA | NA | NA | NA | - | NA | - | 0 | 100 |
|  | A+C | NA | NA | NA | - | NA | - | 0 | 100 |
| Manzano-Fernandez S^[27]^ | A+C+VKA | 88.2 | 11.8 | NA | - | 1 (1-2) | Median (IQR) | 29 | 71 |
|  | A+C | 92 | 8 | NA | - | 1 (1-3) | Median (IQR) | 38 | 62 |
| Ruiz-Nodar JM^[28]^ | A+C+VKA | 82 | 18.6 | NA | - | NA | - | NA | NA |
|  | A+C | 84.4 | 15.6 | NA | - | NA | - | NA | NA |
| Goto K^[29]^ | A+C+VKA | 33.2 | 66.8 | NA | - | NA | - | 47.8 | 52.2 |
|  | A+C | 40.7 | 59.3 | NA | - | NA | - | 56.1 | 43.9 |
| Jang SW^[30]^ | A+C+VKA | 50 | 50 | NA | - | 1.5 ± 0.6 | Mean | 10.7 | 89.3 |
|  | A+C | 59.4 | 40.6 | NA | - | 1.7 ± 0.9 | Mean | 8.6 | 91.4 |
| Valencia J^[31]^ | A+C+VKA  A+C | 74.3 | 25.7 | NA | - | NA | - | 40 | 60 |
|  | A+C |  |  |  |  |  |  |  |  |
| ISAR-TRIPLE^[32]^ | A+C+VKA | 31 | 69.1 | NA | - | NA | - | 0 | 100 |
|  | A+VKA | 33.3 | 66.8 | NA | - | NA | - | 0.5 | 99.5 |
| Choi H^[33]^ | A+C+VKA | 57.3 | 42.7 | NA | - | 1.9 | Mean | 0 | 100 |
|  | A+C | 53 | 47.1 | NA | - | 2.1 | Mean | 0 | 100 |

**Abbreviations:** NA, Not available; PCI, percutaneous coronary intervention; ACS, acute coronary syndrome; SCAD, stable coronary artery disease; BMS, bare-metal stent; DES, drug-eluting stent; IQR, Interquartile range; A+C, aspirin + clopidogrel; A+C+LMWH, aspirin + clopidogrel + low-molecular weight heparin; A+C+VKA, aspirin + clopidogrel + vitamin K antagonist; A+P+VKA, aspirin + prasugrel + vitamin K antagonist; A+T+VKA, aspirin + ticagrelor + vitamin K antagonist; A+VKA, aspirin + vitamin K antagonist; C+VKA, clopidogrel + vitamin K antagonist; T+VKA, ticagrelor + vitamin K antagonist

## eTable 5.4 Indication of anticoagulant therapy & Risk scores (CHADS_2_ score & HASBLED score) among non-RCTs

| **Study group/ First author** | **Regimen** | **Indication of anticoagulant** | | | | | **Thromboembolic risk**  **(patients with AF only)** | | | **Bleeding risk**  **(patients with AF only)** |
| --- | --- | --- | --- | --- | --- | --- | --- | --- | --- | --- |
|  |  | **AF (%)** | **MHV (%)** | **LV**  **thrombus (%)** | **ACS/ dyskinesia (%)** | **VTE (%)** | **Average CHADS_2_ score** | | **CHADS_2_ ≥ 2**  **(%)** | **Average HASBLED score** |
| MUSICA registry^[7]^ | A+C+VKA | 64.7 | 17.3 | NA | NA | NA | NA | - | NA | NA |
|  | A+VKA/C+VKA | 58.7 | 28.3 | NA | NA | NA | NA | - | NA | NA |
|  | A+C | 82.7 | 0 | NA | NA | NA | NA | - | NA | NA |
| Gao F^[8]^ | A+C+VKA | 100 | 0 | 0 | 0 | 0 | NA | - | 49.6 | NA |
|  | A+VKA/C+VKA | 100 | 0 | 0 | 0 | 0 | NA | - | 60.9 | NA |
|  | A+C | 100 | 0 | 0 | 0 | 0 | NA | - | 37.8 | NA |
| WAR-STENT registry^[9]^ | A+C+VKA | 79 | 8 | 4 | NA | 5 | NA | - | NA | NA |
|  | A+VKA/C+VKA | 85 | 5 | 5 | NA | 0 | NA | - | NA | NA |
|  | A+C | 64 | 0 | 0 | NA | 12 | NA | - | NA | NA |
| AFCAS registry^[10]^ | A+C+VKA | 100 | 0 | 0 | 0 | 0 | 2.3 ± 1.2 | Mean | 71 | 2.9 ± 0.7 |
|  | C+VKA | 100 | 0 | 0 | 0 | 0 | 2.4 ± 1.3 | Mean | 77 | 2.9 ± 0.8 |
|  | A+C | 100 | 0 | 0 | 0 | 0 | 2.1 ± 1.2 | Mean | 65 | 2.9 ± 0.8 |
| De Vecchis R^[11]^ | A+C+VKA | 85.4 | 27 | NA | NA | NA | 1.8 ± 1.5** | Mean | NA | 2.3 ± 0.5** |
|  | A+VKA/C+VKA | 90 | 25.8 | NA | NA | NA | 1.8 ± 1.5** | Mean | NA | 2.3 ± 0.5** |
|  | A+C | 73.6 | 0 | NA | NA | NA | 1.8 ± 1.5** | Mean | NA | 2.3 ± 0.5** |
| Sarafoff N^[12]^ | A+C+VKA | 80.3 | 4.8 | 6.2 | NA | 7.3 | NA | - | NA | NA |
|  | A+P+VKA | 28.6 | 9.5 | 33.3 | NA | 19 | NA | - | NA | NA |
| Braun OO ^[13]^ | A+C+VKA | 39.6 | 3.8 | 10.7 | 37.8 | 6.9 | 4.5 ± 1.5 | Mean | NA | 2.2 ± 1 |
|  | T+VKA | 77.6 | 4.7 | 2.8 | 4.7 | 9.3 | 4.8 ± 1.1 | Mean | NA | 2.2 ± 0.8 |
| Fu A ^[14]^ | A+C+VKA | 48.8 | 5.6 | 34.4 | NA | 8 | 2.6 ± 1.3 | Mean | NA | NA |
|  | A+T+VKA | 11.1 | 0 | 77.7 | NA | 3.7 | 2 ± 0 | Mean | NA | NA |
| GRACE registry ^[15]^ | A+C+VKA  A+VKA  C+VKA | 40 | 2.5 | NA | 43 | 4 | NA | - | NA | NA |
|  | *data of each group was not available | | | | | | | | | |
| Suh SY^[16]^ | A+C+VKA | 100 | 0 | 0 | 0 | 0 | 1.81 ± 1.18 | Mean | 56.8 | 1.81 ± 0.61 |
|  | A+C | 100 | 0 | 0 | 0 | 0 | 1.95 ± 1.2 | Mean | 64.5 | 2.0 ± 0.64 |
| STENTICO^[17]^ | A+C+VKA | 41 | 18 | NA | NA | 17 | NA | - | NA | NA |
|  | A+C | 53 | 11 | NA | NA | 9 | NA | - | NA | NA |
| REAL registry^[18]^ | A+C+VKA  A+VKA  A+C | 58 | 7 | 3 | 1 | 10 | NA | - | NA | NA |
|  | *data of each group was not available | | | | | | | | | |
| Ho KW^[19]^ | A+C+VKA | 100 | 0 | 0 | 0 | 0 | 2.6 ± 1.2 | Mean | NA | NA |
|  | A+C | 100 | 0 | 0 | 0 | 0 | 2.1 ± 1.1 | Mean | NA | NA |
| Dabrowska M^[20]^ | A+C+VKA | 100 | 0 | 0 | 0 | 0 | 2.12 ± 1.18** | Mean | NA | NA |
|  | A+C | 100 | 0 | 0 | 0 | 0 | 2.12 ± 1.18** | Mean | NA | NA |
| Hess CN^[21]^ | A+C+VKA | 100 | 0 | 0 | 0 | 0 | NA | - | 79 | Reported as ATRIA score |
|  | A+C | 100 | 0 | 0 | 0 | 0 | NA | - | 73.4 | Reported as ATRIA score |
| Kang DO ^[22]^ | A+C+VKA | 100 | 0 | 0 | 0 | 0 | 2.06 ± 1.18 | Mean | 68.7 | 2.24 ± 0.76 |
|  | A+C | 100 | 0 | 0 | 0 | 0 | 1.68 ± 1.15 | Mean | 52.1 | 2.13 ± 0.74 |
| Caballero L^[23]^ | A+C+VKA | 100 | 0 | 0 | 0 | 0 | 2.78 ± 1.16** | Mean | NA | 3.05 ± 0.78 |
|  | A+C | 100 | 0 | 0 | 0 | 0 | 2.78 ± 1.16** | Mean | NA | 3.05 ± 0.78 |
| Sambola A^[24]^ | A+C+VKA | 100 | 0 | 0 | 0 | 0 | NA | - | 55.5 | NA |
|  | A+C | 100 | 0 | 0 | 0 | 0 | NA | - | 44.5 | NA |
| Maegdefessel L^[25]^ | A+C+VKA | 100 | 0 | 0 | 0 | NA | NA | - | NA | NA |
|  | A+C+LMWH | 100 | 0 | 0 | 0 | NA | NA | - | NA | NA |
|  | A+C | 100 | 0 | 0 | 0 | NA | NA | - | NA | NA |
| Sarafoff N^[26]^ | A+C+VKA | 67 | 17 | NA | 0 | 0 | NA | - | NA | NA |
|  | A+C | 93 | 0 | NA | 0 | 0 | NA | - | NA | NA |
| Manzano-Fernandez S^[27]^ | A+C+VKA | 100 | 0 | 0 | 0 | 0 | 2 (2-3) | Median (IQR) | NA | NA |
|  | A+C | 100 | 0 | 0 | 0 | 0 | 2 (1-3) | Median (IQR) | NA | NA |
| Ruiz-Nodar JM^[28]^ | A+C+VKA | 100 | 0 | 0 | 0 | 0 | 2 (1-3) | Median (IQR) | NA | NA |
|  | A+C | 100 | 0 | 0 | 0 | 0 | 2 (1-3) | Median (IQR) | NA | NA |
| Goto K^[29]^ | A+C+VKA | 100 | 0 | 0 | 0 | 0 | 2.4 ± 1.2 | Mean | 76.9 | NA |
|  | A+C | 100 | 0 | 0 | 0 | 0 | 2.4 ± 1.3 | Mean | 73.7 | NA |
| Jang SW^[30]^ | A+C+VKA | 100 | 0 | 0 | 0 | 0 | 1.6 ± 1.4 | Mean | NA | NA |
|  | A+C | 100 | 0 | 0 | 0 | 0 | 1.6 ± 1.2 | Mean | NA | NA |
| Valencia J^[31]^ | A+C+VKA  A+C | 68.6 | 10 | NA | NA | NA | NA | - | NA | NA |
|  | *data of each group was not available | | | | | | | | | |
| ISAR-TRIPLE^[32]^ | A+C+VKA | 85 | 9.1 | NA | NA | 3.6 | NA | - | 78.9 | NA |
|  | A+VKA | 82.7 | 5.5 | NA | NA | 7.5 | NA | - | 83.4 | NA |
| Choi H^[33]^ | A+C+VKA | 100 | 0 | 0 | 0 | 0 | 2.88 ± 1.82* | Mean | 73.3 | NA |
|  | A+C | 100 | 0 | 0 | 0 | 0 | 2.53 ± 1.58* | Mean | 71.1 | NA |

**Abbreviations:** NA, Not available; AF, Atrial fibrillation; MHV, mechanical heart valve; LV, Left ventricular; ACS, Acute coronary syndrome; VTE, Venous thromboembolism; IQR, Interquartile range; A+C, aspirin + clopidogrel; A+C+LMWH, aspirin + clopidogrel + low-molecular weight heparin; A+C+VKA, aspirin + clopidogrel + vitamin K antagonist; A+P+VKA, aspirin + prasugrel + vitamin K antagonist; A+T+VKA, aspirin + ticagrelor + vitamin K antagonist; A+VKA, aspirin + vitamin K antagonist; C+VKA, clopidogrel + vitamin K antagonist; T+VKA, ticagrelor + vitamin K antagonist

* CHA_2_DS_2_-VASc score

## eTable 5.5 Detail of regimens used in each study of non-RCTs

| **Study name/ First author**  **(year)** | **Regimen** | **Type of OAC** | **INR target** | **TTR**  **(%)** | **VKA**  **Naïve (%)** | **Dose of ASA**  **(mg)** | **Dose of P2Y_12_ inhibitor (mg)** | **Specified period of regimen use** | **Duration of regimen used/ percent continuation at the point of outcome measurement** |
| --- | --- | --- | --- | --- | --- | --- | --- | --- | --- |
| MUSICA registry^[7]^ | A+C+VKA  A+VKA  C+VKA  A+C | Warfarin, LMWH | NA | NA | NA | NA | NA | NA | NA |
| Gao F^[8]^ | A+C+VKA | Warfarin | 1.8 - 2.5  (specified) | 72% | 26.1 | NA | NA | NA | Triple Therapy at 1 year = 47% |
|  | A+VKA  C+VKA | Warfarin | 1.8 - 2.5  (specified) | 72% | 20.7 | NA | NA | Antiplatelets trough12 months in dual therapy & dual antiplatelet | Dual therapy at 1 year = 92.8% |
|  | A+C | - | - | - | - | NA | NA | Antiplatelets trough12 months in dual therapy & dual antiplatelet | Dual antiplatelets at 1 year = 95.5% |
| WAR-STENT registry^[9]^ | A+C+VKA | Warfarin | 2.0 – 3.0 (70%);  1.8 - 2.5 (29%)  (not specified) | NA | NA | NA | NA | NA | Duration of P2Y_12_ inhibitor used = 4 weeks (0-78)  (outcome measurement = 1 year) |
|  | A+VKA  C+VKA | Warfarin | 2.0 – 3.0 (95%)  (not specified) | NA | NA | NA | NA | NA | Duration of P2Y_12_ inhibitor used = 4 weeks (0-52)  (outcome measurement = 1 year) |
|  | A+C | - | - | NA | NA | NA | NA | NA | NA |
| AFCAS registry^[10]^ | A+C+VKA | VKA | 2.0-3.0  (specified) | NA | NA | NA | NA | NA | Prescribed duration of OAC = lifelong  Prescribed duration of clopidogrel; 1 month = 44%, 3 months = 20%, 6 months = 10%, 12 months = 20%  Prescribed duration of aspirin = lifelong (60%) |
|  | C+VKA | VKA | 2.0-3.0  (specified) | NA | NA | NA | NA | VKA lifelong, clopidogrel was either 1 or 12 months | Prescribed duration of OAC = lifelong  Prescribed duration of clopidogrel; 1 month = 37%, 3 months = 16%, 6 months = 4%, 12 months = 34% |
|  | A+C | VKA | - | NA | NA | NA | NA | DAPT 12 months then ASA lifelong | Prescribed duration of clopidogrel; 1 month = 23%, 3 months = 6%, 6 months = 3%, 12 months = 53%  Prescribed duration of aspirin = lifelong (60%) |
| De Vecchis R^[11]^ | A+C+VKA  A+VKA  C+VKA  A+C | Warfarin | NA | NA | NA | NA | NA | The therapy was either stopped after 1 month if a BMS had been implanted or was interrupted after a variable period (extending from 1 to 3 months) if a DES had been used. Antiaggregant agents was prescribed for a period of 1 month (BMS) or 9-12 months (DES) | NA |
| Sarafoff N^[12]^ | A+C+VKA | Phenpro-coumon | 2.0-2.5 (AF),  2.5-3.0 (MHV)  (specified) | NA | NA | NA | 75 OD | NA | NA |
|  | A+P+VKA | Phenpro-coumon | 2.0-2.5 (AF),  2.5-3.0 (MHV)  (specified) | NA | NA | NA | 10 OD;  5 OD in less than 75 y/o or < 60 kg | NA |  |
| Braun OO^[13]^ | A+C+VKA | Warfarin | 2.0-3.0  (2.0-2.5 = 47.8%)  (specified) | 75.8 | 66.7 | 75 OD | 75 OD | In general, if warfarin was terminated the patients continued with a P2Y12 inhibitor and aspirin. If a P2Y12 inhibitor was terminated, the patients continued with warfarin and aspirin | Mean duration of treatment = 2.5 ± 0.9 months  (outcome measurement = 3 months) |
|  | T+VKA | Warfarin | 2.0-3.0  (2.0-2.5 = 1.9%)  (specified) | 71.5 | 48.6 | x | 90 BID | In general, if warfarin was terminated the patients continued with a P2Y12 inhibitor and aspirin. If a P2Y12 inhibitor was terminated, the patients continued with warfarin and aspirin | Mean duration of treatment = 2.7 ± 0.8 months  (outcome measurement = 3 months) |
| Fu A ^[14]^ | A+C+VKA  A+T+VKA | VKA | NA | NA | NA | NA | NA | NA | NA |
| GRACE registry ^[15]^ | A+C+VKA | Warfarin | NA | NA | 72% | NA | NA | Not defined | At the point of outcome measure; Triple therapy = 24%, Dual Therapy = 49%, Warfarin only = 27% |
|  | A+VKA  C+VKA | Warfarin | NA | NA | 72% | NA | NA | Not defined | At the point of outcome measure; Dual antiplatelet = 12%, Dual Therapy = 49%, Warfarin only = 39% |
| Suh SY^[16]^ | A+C+VKA  A+C | Warfarin | NA | NA | NA | NA | NA | NA | NA |
| STENTICO^[17]^ | A+C+VKA  A+C | OAC | NA | NA | NA | NA | NA | NA | NA |
| REAL registry^[18]^ | A+C+VKA  A+VKA  A+C | OAC | NA | NA | NA | NA | NA | NA | NA |
| Ho KW^[19]^ | A+C+VKA | Warfarin | NA | NA | NA | NA | NA | NA | Duration of Triple therapy = 5.8 ± 5.0 months  (outcome measurement = 6 months) |
|  | A+C | Warfarin | NA | NA | NA | NA | NA | NA | Duration of Dual antiplatelet = 6.1 ± 4.9 months  (outcome measurement = 6 months) |
| Dabrowska  M^[20]^ | A+C+VKA | Warfarin or Acenocoumarol | Standard dose | NA | NA | Standard dose | Standard dose | NA | Triple therapy at 12 months = 41% |
|  | A+C | - | - | - | - | Standard dose | Standard dose | NA | Dual antiplatelet at 12 months = 48.3% |
| Hess CN ^[21]^ | A+C+VKA | Warfarin | NA | NA | 37.9 | NA | NA | NA | OAC continuation at 3 months = 93.4%, 6 months = 91,8%, 12 months = 57.4%  Clopidogrel continuation at 3 months = 95.9%, 6 months = 94.6%, 12 months = 63.7%  (outcome measurement = 2 years) |
|  | A+C | - | - | - | - | NA | NA | NA | Clopidogrel continuation at 3 months = 94.7%, 6 months = 94.4%, 12 months = 73.4%  (outcome measurement = 2 years) |
| Kang DO ^[22]^ | A+C+VKA | Warfarin | 2.0-3.0 | 29.2 ± 24.9 | NA | Standard dose | Standard dose | NA | Mean Triple therapy duration = 352.4 ± 144.0 days  (outcome measurement = 1.7 years) |
|  | A+C | - | - | - | - | Standard dose | Standard dose | NA | NA |
| Caballero L^[23]^ | A+C+VKA  A+C | OAC | NA | NA | NA | NA | NA | NA | NA |
| Sambola A ^[24]^ | A+C+VKA | VKA | 2.0-2.5 | วัดเป็น % INR in range (P.356) | NA | 100 OD | 75 OD | NA | BMS; duration of Triple therapy = 1.5 ± 1.0 months  (population in this study predominate by BMS implantation) |
|  | A+C | - | 2.0-2.5 | - | NA | 100 OD | 75 OD | 1 antiplatelet agent should be stopped at least 1 month following PCI when a bare-metal stent (BMS) was used, and between 3 and 12 months when a DES was used. | NA |
| Maegdefessel L^[25]^ | A+C+VKA  A+C+LMWH  A+C | OAC | NA | NA | 42.9 | NA | NA | NA | NA |
| Sarafoff N^[26]^ | A+C+VKA | Phenpro-coumon | 2.5 for MHV, 2.0 for other conditions | NA | NA | 100 BID | 75 OD | NA | Median duration of Triple therapy continuation = 12 weeks, after that switched to A+VKA |
|  | A+C | - | - | - | - | 100 BID | 75 OD | NA | Median duration of dual antiplatelet = 4 weeks, after that switched to A+VKA |
| Manzano-Fernandez S^[27]^ | A+C+VKA | Acenocou-marol | NA | NA | 27 | NA | NA | NA | Median duration of clopidogrel use = 12 months (1-12) |
|  | A+C | - | - | - | - | NA | NA | NA | Median duration of clopidogrel use = 7 months (1-12) |
| Ruiz-Nodar JM^[28]^ | A+C+VKA  A+C | Coumarins | NA | NA | 30.8 | NA | NA | Either coumarins or 1 antiplatelet agent being stopped at 1 month following PCI where bare-metal stents were used and between 3 and 12 months when a DES was used | NA |
| Goto K^[29]^ | A+C+VKA  A+C | Warfarin | 1.6-2.6 for patients > 70 years | 24.2 % for 2-3 &  52.6 % for 1.6-2.6 | NA | 81 OD | 75 OD | NA | After 5 years, OAC was discontinued in 22.7% |
| Jang SW^[30]^ | A+C+VKA  A+C | Warfarin | 2.0-3.0 | 52 | NA | NA | NA | Aspirin, clopidogrel and cilostazol were usually administered at least 1 year after the procedure regardless of the type of stent used | 17% of the patients discontinued warfarin within 1 year due to side effects or poor compliance |
| Valencia J^[31]^ | A+C+VKA | OAC | NA | NA | NA | NA | NA | NA | Continuation of Triple therapy; 1 month = 55%, 6 months = 33%, 12 months = 17% |
|  | A+C | - | NA | NA | NA | NA | NA | NA | Continuation of dual antiplatelet; 1 month = 83%, 6 months = 55%, 12 months = 29% |
| ISAR-TRIPLE^[32]^ | A+C+VKA | Warfarin or phenprocoumon | lowest recommended target INR; median = 2.3 (2.0-2.6) | 68.9 | 0 | 75-200 OD | 75 OD | Triple therapy along study period | Continuation of OAC use at 6 months = 90.6%  Compliance with assigned clopidogrel at 6 months = 86.6%  Continuation of aspirin at 6 months = 95% |
|  | A+VKA | Warfarin or phenprocoumon | lowest recommended target INR; median = 2.3 (2.0-2.6) | 68.9 | 0 | 75-200 OD | - | Aspirin + OAC along study period | Continuation of OAC use at 6 months = 90.6%  Continuation of aspirin at 6 months = 95% |
| Choi H^[33]^ | A+C+VKA | Warfarin | 2.0 – 3.0 | NA | NA | 100 – 200 OD | 75 OD | Indefinite use of aspirin, use of clopidogrel for 6 – 12 months | Aspirin maintenance at 1 year = 93.8%  Clopidogrel maintenance at 1 year = 75.4%  Warfarin maintenance at 1 year = 75.4% |
|  | A+C | - | - | - | NA | 100 – 200 OD | 75 OD | Indefinite use of aspirin, use of clopidogrel for 6 – 12 months | Aspirin maintenance at 1 year = 96.1%  Clopidogrel maintenance at 1 year = 76.3% |

**Abbreviation:** OAC, Oral anticoagulant; TTR, Time in therapeutic range; VKA, Vitamin K antagonist; ASA, Aspirin; INR, International normalized ratio; NA, Not available; ACS, Acute coronary syndrome; SCAD, Stable coronary artery disease; BMS, Bare-metal stent; DES, Drug-eluting stent; AF, Atrial fibrillation; MHV, Mechanical heart valve; A+C, aspirin + clopidogrel; A+C+LMWH, aspirin + clopidogrel + low-molecular weight heparin; A+C+VKA, aspirin + clopidogrel + vitamin K antagonist; A+P+VKA, aspirin + prasugrel + vitamin K antagonist; A+T+VKA, aspirin + ticagrelor + vitamin K antagonist; A+VKA, aspirin + vitamin K antagonist; C+VKA, clopidogrel + vitamin K antagonist; T+VKA, ticagrelor + vitamin K antagonist

*Median (Range)

## eTable 5.6 Summarized risk of bias of included non-RCTs using ROBINS-I (Risk Of Bias In Non-randomized Studies - of Interventions) tool

| **Study Name/ First Author** | **Confounding** | **Participant Selection** | **Classification of intervention** | **Deviation from intended intervention** | **Missing data** | **Measurement of outcomes** | **Selection of the reported results** | **Overall** |
| --- | --- | --- | --- | --- | --- | --- | --- | --- |
| MUSICA registry^[7]^ | Serious | NI | Moderate | NI | Low | Serious | Low | **Serious** |
| Gao F^[8]^ | Serious | NI | Moderate | NI | Serious | Serious | Moderate | **Serious** |
| WAR-STENT registry^[9]^ | Serious | NI | Moderate | NI | NI | NI | Low | **Serious** |
| AFCAS registry^[10]^ | Low | NI | Moderate | NI | NI | Serious | Moderate | **Serious** |
| De Vecchis R^[11]^ | Serious | NI | Moderate | NI | NI | Serious | Low | **Serious** |
| Sarafoff N^[12]^ | Serious | NI | Low | NI | NI | Low | Low | **Serious** |
| Braun OO^[13]^ | Serious | NI | Moderate | NI | Moderate | Serious | Low | **Serious** |
| Fu A^[14]^ | Moderate | NI | Moderate | NI | NI | Serious | Serious | **Serious** |
| GRACE registry^[15]^ | Serious | NI | NI | NI | Critical | Serious | Low | **Critical** |
| Suh SY^[16]^ | Low | NI | Moderate | NI | NI | Serious | Low | **Serious** |
| STENTICO^[17]^ | Low | Low | Low | NI | Low | Serious | Moderate | **Serious** |
| REAL registry^[18]^ | NI | NI | Low | NI | NI | Moderate | Low | **Moderate** |
| Ho KW^[19]^ | Serious | NI | Moderate | NI | NI | Serious | Moderate | **Serious** |
| Dabrowska M^[20]^ | Serious | NI | Low | NI | Low | Serious | Low | **Serious** |
| Hess CN^[21]^ | Moderate | NI | Moderate | NI | NI | Serious | Low | **Serious** |
| Kang DO^[22]^ | Moderate | NI | Moderate | NI | NI | Serious | Low | **Serious** |
| Caballero L^[23]^ | NI | NI | Moderate | NI | NI | Serious | Low | **Serious** |
| Sambola A^[24]^ | Low | NI | Moderate | NI | NI | Moderate | Low | **Moderate** |
| Maegdefessel L^[25]^ | Low | NI | NI | NI | NI | Serious | Low | **Serious** |
| Sarafoff N^[26]^ | NI | NI | Low | NI | NI | Low | Low | **Low** |
| Manzano-Fernandez^[27]^ | Serious | NI | NI | NI | NI | Serious | Low | **Serious** |
| Ruiz-Nodar^[28]^ | Serious | NI | NI | NI | NI | Serious | Low | **Serious** |
| Goto K^[29]^ | Moderate | NI | Moderate | NI | NI | NI | Low | **Moderate** |
| Jang SW^[30]^ | Serious | NI | Moderate | NI | NI | Moderate | Low | **Serious** |
| Valencia J^[31]^ | NI | NI | NI | NI | NI | Moderate | Low | **Moderate** |
| ISAR-TRIPLE^[32]^ | NI | Low | Low | NI | NI | Low | Low | **Low** |
| Choi H^[33]^ | Moderate | Low | Low | NI | Low | Low | Low | **Moderate** |

**Abbreviation:** NI, No information

# Appendix 6

**Results of meta-analyses of direct comparisons of treatment options**

## eTable 6 Pairwise meta-analysis risk ratio (and 95% CI) for all dichotomous outcomes

| **Comparisons** | | **No. studies** | **No. of events/**  **Total No. of Treatment 1** | **No. of events/**  **Total No. of Treatment 2** | **Pairwise**  **meta-analysis**  **risk ratio (95% CI)** | **Heterogeneity I^2^ (variation in RR attributable to heterogeneity)** |
| --- | --- | --- | --- | --- | --- | --- |
| **Major bleeding** | | | | | | |
| **Comparator** | **Intervention** |  | | | | |
| A+C+VKA | A+C | 13 |  |  | **0.58 (0.40 to 0.83)** | 54.0% |
| C+VKA | A+C | 1 |  |  | 1.80 (0.70 to 4.62) | NA |
| A+C+VKA | C+VKA | 1 |  |  | 0.68 (0.28 to 1.61) | NA |
| A+C+VKA | T+VKA | 1 |  |  | 0.59 (0.12 to 3.03) | NA |
| A+C+VKA | A+T+VKA | 1 |  |  | 0.93 (0.29 to 2.94) | NA |
| A+C+VKA | A+VKA | 1 |  |  | 0.58 (0.25 to 1.37) | 0.0% |
| A+C+LMWH | A+C | 1 |  |  | 2.07 (0.10 to 42.17) | NA |
| A+C+VKA | A+C+LMWH | 1 |  |  | * | * |
| A+C+VKA | A+P+VKA | 1 |  |  | **5.00 (1.52 to 16.67)** | NA |
| **Stroke** | | | | | | |
| **Comparator** | **Intervention** |  | | | | |
| A+C+VKA | A+C | 17 |  |  | **1.60 (1.04 to 2.45)** | 39.8% |
| C+VKA | A+C | 1 |  |  | 3.15 (0.40 to 25.17) | NA |
| A+C+VKA | C+VKA | 2 |  |  | 1.56 (0.34 to 7.14) | 24.9% |
| A+C+VKA | T+VKA | 1 |  |  | 0.74 (0.07 to 8.33) | NA |
| A+C+VKA | A+T+VKA | 1 |  |  | 0.35 (0.02 to 5.88) | NA |
| A+C+VKA | A+VKA | 3 |  |  | 1.54 (0.21 to 11.11) | 58.1% |
| C+VKA | A+VKA | 1 |  |  | 2.07 (0.39 to 11.01) | NA |
| A+C+LMWH | A+C | 1 |  |  | 0.92 (0.30 to 2.82) | NA |
| A+C+VKA | A+C+LMWH | 1 |  |  | 3.14 (0.18 to 54.93) | NA |
| A+VKA | A+C | 1 |  |  | 3.76 (0.84 to 16.75) | NA |
| A+C+VKA | A+P+VKA | 1 |  |  | 2.86 (0.36 to 25.00) | NA |
| **Myocardial infarction** | | | | | | |
| **Comparator** | **Intervention** |  | | | | |
| A+C+VKA | A+C | 15 |  |  | 1.23 (0.92 to 1.64) | 26.0% |
| C+VKA | A+C | 1 |  |  | 0.45 (0.12 to 1.75) | NA |
| A+C+VKA | C+VKA | 2 |  |  | 1.12 (0.53 to 2.33) | 0.0% |
| A+C+VKA | T+VKA | 1 |  |  | 1.47 (0.30 to 7.14) | NA |
| A+C+VKA | A+T+VKA | 1 |  |  | 2.33 (0.45 to 12.5) | NA |
| A+C+VKA | A+VKA | 2 |  |  | 1.20 (0.35 to 4.17) | NA |
| C+VKA | A+VKA | 1 |  |  | 0.79 (0.18 to 3.41) | NA |
| A+C+LMWH | A+C | 1 |  |  | 3.72 (0.20 to 67.63) | NA |
| A+C+VKA | A+C+LMWH | 1 |  |  | * | * |
| A+C+VKA | A+P+VKA | 1 |  |  | 1.09 (0.06 to 20.00) | NA |
| **Repeated revascularization** | | | | | | |
| **Comparator** | **Intervention** |  | | | | |
| A+C+VKA | A+C | 7 |  |  | 1.07 (0.85 to 1.34) | 0.0% |
| C+VKA | A+C | 1 |  |  | 0.64 (0.26 to 1.62) | NA |
| A+C+VKA | C+VKA | 2 |  |  | 0.95 (0.56 to 1.61) | 0.0% |
| A+C+VKA | A+VKA | 1 |  |  | 1.61 (0.95 to 2.78) | NA |
| C+VKA | A+VKA | 1 |  |  | 2.19 (0.94 to 5.11) | NA |
| **Stent thrombosis** | | | | | | |
| **Comparator** | **Intervention** |  | | | | |
| A+C+VKA | A+C | 12 |  |  | 1.25 (0.67 to 2.34) | 0.0% |
| C+VKA | A+C | 1 |  |  | 0.68 (0.12 to 3.96) | NA |
| A+C+VKA | C+VKA | 1 |  |  | 2.08 (0.45 to 9.09) | NA |
| A+C+VKA | T+VKA | 1 |  |  | * | * |
| A+C+VKA | A+T+VKA | 1 |  |  | 9.09 (0.87 to 100) | NA |
| A+VKA | A+C | 1 |  |  | 0.83 (0.23 to 3.04) | NA |
| A+C+VKA | A+VKA | 2 |  |  | 1.02 (0.19 to 5.56) | NA |
| A+C+VKA | A+P+VKA | 1 |  |  | 3.23 (0.16 to 50.00) | NA |
| **All-cause death** | | | | | | |
| **Comparator** | **Intervention** |  | | | | |
| A+C+VKA | A+C | 17 |  |  | 1.01 (0.79 to 1.29) | 42.7% |
| C+VKA | A+C | 1 |  |  | 1.62 (0.63 to 4.20) | NA |
| A+C+VKA | C+VKA | 2 |  |  | 0.79 (0.42 to 1.52) | 0.0% |
| A+C+VKA | T+VKA | 1 |  |  | 1.19 (0.33 to 4.35) | NA |
| A+C+VKA | A+T+VKA | 1 |  |  | 0.20 (0.01 to 3.23) | NA |
| A+C+VKA | A+VKA | 2 |  |  | 0.90 (0.26 to 3.13) | 59.7% |
| C+VKA | A+VKA | 1 |  |  | 1.43 (0.47 to 4.34) | NA |
| A+C+LMWH | A+C | 1 |  |  | **0.24 (0.06 to 0.98)** | NA |
| A+C+VKA | A+C+LMWH | 1 |  |  | 1.67 (0.21 to 13.08) | NA |
| A+C+VKA | A+P+VKA | 1 |  |  | 2.44 (0.59 to 10.00) | NA |

**Abbreviations:** A+C, aspirin + clopidogrel; A+C+LMWH, aspirin + clopidogrel + low-molecular weight heparin; A+C+VKA, aspirin + clopidogrel + vitamin K antagonist; A+P+VKA, aspirin + prasugrel + vitamin K antagonist; A+T+VKA, aspirin + ticagrelor + vitamin K antagonist; A+VKA, aspirin + vitamin K antagonist; C+VKA, clopidogrel + vitamin K antagonist; T+VKA, ticagrelor + vitamin K antagonist

* excluded

# Appendix 7

**Assessment of global inconsistency for each outcome network in main analysis**

## eTable 7 Assessment of global inconsistency in networks using the ‘design-by-treatment’ interaction model

| **Network outcome** | **Chi-square** | **P value for test of global inconsistency** |
| --- | --- | --- |
| **Non-randomized studies** | | |
| Major bleeding | 3.06 | 0.2166 |
| Stroke or systemic embolism | 6.39 | 0.3809 |
| Myocardial infarction | 4.53 | 0.3386 |
| Repeated revascularization | 2.01 | 0.3655 |
| Stent thrombosis | 0.27 | 0.9649 |
| All-cause death | 3.75 | 0.4413 |

# Appendix 8

**Results of Network meta-analysis (Non-RCTs)**

Treatment options are in order of their efficacy or safety ranking. Estimates are presented as risk ratios (RR) and 95% confidence intervals. Treatments are ordered by rankings for each outcome.

Comparisons between treatments should be read from column to row for each outcome (row treatment is reference). Risk ratios less than 1 favor the column-defining treatment. To obtain risks ratios for comparisons in the opposite direction, reciprocals should be taken. Significant results are in bold and underlined. Blue-color box represents that unfavorable outcome was decreased. In contrast, red-color box represents that unfavorable outcome was increased.

## eTable 8.1 Results of network meta-analysis of treatment options on major bleeding

The following table shows the effect sizes (risk ratio) and the rank order (SUCRA ranks) compared to aspirin + clopidogrel + vitamin K antagonist

| **Treatment name** | **Risk ratio** | **95% Confidence interval** | **p-value** | **SUCRA rank** |
| --- | --- | --- | --- | --- |
| A+C+LMWH | 0.29 | 0.01, 6.52 | 0.436 | 1 |
| C+VKA | 0.48 | 0.14, 1.61 | 0.236 | 2 |
| A+C* | **0.57** | **0.39, 0.84** | **0.005** | 3 |
| A+VKA | 0.58 | 0.16, 2.05 | 0.400 | 4 |
| T+VKA | 0.59 | 0.09, 3.84 | 0.583 | 5 |
| A+T+VKA | 0.93 | 0.21, 4.12 | 0.920 | 6 |
| A+C+VKA* | reference | | | 7 |
| A+P+VKA | **5.09** | **1.10, 23.44** | **0.037** | 8 |
| **Number of studies** | **17** | **Number of studies with adjustment of confounding factors** | **4** |  |

*regimens adjusted mainly for these confounding factors: age, sex, dyslipidemia, heart failure, renal impairment, history of stroke, indication of PCI, DES implantation

**Abbreviations:** A+C, aspirin + clopidogrel; A+C+LMWH, aspirin + clopidogrel + low-molecular weight heparin; A+C+VKA, aspirin + clopidogrel + vitamin K antagonist; A+P+VKA, aspirin + prasugrel + vitamin K antagonist; A+T+VKA, aspirin + ticagrelor + vitamin K antagonist; A+VKA, aspirin + vitamin K antagonist; C+VKA, clopidogrel + vitamin K antagonist; T+VKA, ticagrelor + vitamin K antagonist

## eFigure 8.1 Network estimated risk ratios (95% confidence intervals) of treatment options on major bleeding

| **A+C+LMWH** |  |  |  |  |  |  |  |
| --- | --- | --- | --- | --- | --- | --- | --- |
| 0.60 (0.02,16.62) | **C+VKA** |  |  |  |  |  |  |
| 0.51 (0.02,11.18) | 0.84 (0.25,2.81) | **A+C*** |  |  |  |  |  |
| 0.50 (0.02,14.34) | 0.83 (0.15,4.75) | 0.99 (0.26,3.70) | **A+VKA** |  |  |  |  |
| 0.49 (0.01,18.47) | 0.82 (0.09,7.53) | 0.97 (0.14,6.54) | 0.98 (0.10,9.36) | **T+VKA** |  |  |  |
| 0.31 (0.01,9.89) | 0.52 (0.08,3.55) | 0.62 (0.13,2.90) | 0.63 (0.09,4.43) | 0.64 (0.06,7.00) | **A+T+VKA** |  |  |
| 0.06 (0.00,1.83) | 0.09 (0.01,0.66) | 0.11 (0.02,0.55) | 0.11 (0.02,0.83) | 0.12 (0.01,1.30) | 0.18 (0.02,1.54) | **A+P+VKA** |  |
| 0.29 (0.01,6.52) | 0.48 (0.14,1.61) | 0.57 (0.39,0.84) | 0.58 (0.16,2.05) | 0.59 (0.09,3.84) | 0.93 (0.21,4.12) | 5.09 (1.10,23.44) | **A+C+VKA*** |

*regimens adjusted mainly for these confounding factors: age, sex, dyslipidemia, heart failure, renal impairment, history of stroke, indication of PCI, DES implantation

**Abbreviations:** A+C, aspirin + clopidogrel; A+C+LMWH, aspirin + clopidogrel + low-molecular weight heparin; A+C+VKA, aspirin + clopidogrel + vitamin K antagonist; A+P+VKA, aspirin + prasugrel + vitamin K antagonist; A+T+VKA, aspirin + ticagrelor + vitamin K antagonist; A+VKA, aspirin + vitamin K antagonist; C+VKA, clopidogrel + vitamin K antagonist; T+VKA, ticagrelor + vitamin K antagonist

Treatments are ordered by SUCRA rank. Comparisons between treatments should be read from column to row for each outcome (row treatment is reference).

## eTable 8.2 Results of network meta-analysis of treatment options on stroke and/ or systemic embolism

The following table shows the effect sizes (risk ratio) and the rank order (SUCRA ranks) compared to aspirin + clopidogrel + vitamin K antagonist

| **Treatment name** | **Risk ratio** | **95% Confidence interval** | **p-value** | **SUCRA rank** |
| --- | --- | --- | --- | --- |
| A+T+VKA | 0.35 | 0.02, 7.51 | 0.499 | 1 |
| T+VKA | 0.74 | 0.05, 10.57 | 0.825 | 2 |
| A+C+VKA* | *Reference* | | | 3 |
| C+VKA | 0.93 | 0.21, 4.11 | 0.924 | 4 |
| A+VKA | 1.22 | 0.37, 4.09 | 0.743 | 5 |
| A+C* | **1.69** | **1.06, 2.68** | **0.027** | 6 |
| A+C+LMWH | 2.04 | 0.41, 10.08 | 0.380 | 7 |
| A+P+VKA | 2.83 | 0.26, 30.45 | 0.392 | 8 |
| **Number of studies** | **22** | **Number of studies with adjustment of confounding factors** | **3** |  |

*regimens adjusted mainly for these confounding factors: age, sex, dyslipidemia, heart failure, renal impairment, history of stroke, indication of PCI, DES implantation

**Abbreviations:** A+C, aspirin + clopidogrel; A+C+LMWH, aspirin + clopidogrel + low-molecular weight heparin; A+C+VKA, aspirin + clopidogrel + vitamin K antagonist; A+P+VKA, aspirin + prasugrel + vitamin K antagonist; A+T+VKA, aspirin + ticagrelor + vitamin K antagonist; A+VKA, aspirin + vitamin K antagonist; C+VKA, clopidogrel + vitamin K antagonist; T+VKA, ticagrelor + vitamin K antagonist

## eFigure 8.2 Network estimated risk ratios (95% confidence intervals) of treatment options on stroke and/ or systemic embolism

| **A+T+VKA** |  |  |  |  |  |  |  |
| --- | --- | --- | --- | --- | --- | --- | --- |
| 0.47 (0.01,27.27) | **T+VKA** |  |  |  |  |  |  |
| 0.37 (0.01,11.35) | 0.80 (0.04,16.74) | **C+VKA** |  |  |  |  |  |
| 0.28 (0.01,7.71) | 0.60 (0.03,11.21) | 0.76 (0.15,3.91) | **A+VKA** |  |  |  |  |
| 0.21 (0.01,4.61) | 0.44 (0.03,6.51) | 0.55 (0.12,2.50) | 0.73 (0.21,2.48) | **A+C*** |  |  |  |
| 0.17 (0.01,5.42) | 0.36 (0.02,8.04) | 0.45 (0.05,3.92) | 0.60 (0.08,4.28) | 0.83 (0.18,3.86) | **A+C+LMWH** |  |  |
| 0.12 (0.00,5.98) | 0.26 (0.01,9.27) | 0.33 (0.02,5.43) | 0.43 (0.03,6.23) | 0.60 (0.05,6.73) | 0.72 (0.04,12.68) | **A+P+VKA** |  |
| 0.35 (0.02,7.51) | 0.74 (0.05,10.57) | 0.93 (0.21,4.11) | 1.22 (0.37,4.09) | **1.69 (1.06,2.68)** | 2.04 (0.41,10.08) | 2.83 (0.26,30.45) | **A+C+VKA*** |

*regimens adjusted mainly for these confounding factors: age, sex, dyslipidemia, heart failure, renal impairment, history of stroke, indication of PCI, DES implantation

**Abbreviations:** A+C, aspirin + clopidogrel; A+C+LMWH, aspirin + clopidogrel + low-molecular weight heparin; A+C+VKA, aspirin + clopidogrel + vitamin K antagonist; A+P+VKA, aspirin + prasugrel + vitamin K antagonist; A+T+VKA, aspirin + ticagrelor + vitamin K antagonist; A+VKA, aspirin + vitamin K antagonist; C+VKA, clopidogrel + vitamin K antagonist; T+VKA, ticagrelor + vitamin K antagonist

Treatments are ordered by SUCRA rank. Comparisons between treatments should be read from column to row for each outcome (row treatment is reference)

## eTable 8.3 Results of network meta-analysis of treatment options on myocardial infarction

The following table shows the effect sizes (risk ratio) and the rank order (SUCRA ranks) compared to aspirin + clopidogrel + vitamin K antagonist

| **Treatment name** | **Risk ratio** | **95% Confidence interval** | **p-value** | **SUCRA rank** |
| --- | --- | --- | --- | --- |
| A+C+LMWH | 0.33 | 0.02, 6.07 | 0.453 | 1 |
| A+C+VKA* | *Reference* | | | 2 |
| A+VKA | 1.12 | 0.33, 3.75 | 0.853 | 3 |
| A+P+VKA | 1.08 | 0.06, 18.83 | 0.957 | 4 |
| C+VKA | 1.21 | 0.55, 2.69 | 0.631 | 5 |
| A+C* | 1.20 | 0.88, 1.66 | 0.252 | 6 |
| T+VKA | 1.48 | 0.29, 7.55 | 0.636 | 7 |
| A+T+VKA | 2.31 | 0.43, 12.55 | 0.331 | 8 |
| **Number of studies** | **20** | **Number of studies with adjustment of confounding factors** | **3** |  |

*regimens adjusted mainly for these confounding factors: age, sex, dyslipidemia, heart failure, renal impairment, history of stroke, indication of PCI, DES implantation

**Abbreviations:** A+C, aspirin + clopidogrel; A+C+LMWH, aspirin + clopidogrel + low-molecular weight heparin; A+C+VKA, aspirin + clopidogrel + vitamin K antagonist; A+P+VKA, aspirin + prasugrel + vitamin K antagonist; A+T+VKA, aspirin + ticagrelor + vitamin K antagonist; A+VKA, aspirin + vitamin K antagonist; C+VKA, clopidogrel + vitamin K antagonist; T+VKA, ticagrelor + vitamin K antagonist

## eFigure 8.3 Network estimated risk ratios (95% confidence intervals) of treatment options on myocardial infarction

| **A+C+LMWH** |  |  |  |  |  |  |  |
| --- | --- | --- | --- | --- | --- | --- | --- |
| 0.29 (0.01,6.88) | **A+VKA** |  |  |  |  |  |  |
| 0.30 (0.01,17.98) | 1.04 (0.05,23.05) | **A+P+VKA** |  |  |  |  |  |
| 0.27 (0.01,5.51) | 0.92 (0.24,3.52) | 0.89 (0.05,17.27) | **C+VKA** |  |  |  |  |
| 0.27 (0.01,4.97) | 0.93 (0.27,3.24) | 0.90 (0.05,15.91) | 1.01 (0.44,2.29) | **A+C*** |  |  |  |
| 0.22 (0.01,6.26) | 0.76 (0.10,5.75) | 0.73 (0.03,19.57) | 0.82 (0.13,5.02) | 0.81 (0.15,4.27) | **T+VKA** |  |  |
| 0.14 (0.00,4.13) | 0.48 (0.06,3.87) | 0.47 (0.02,12.92) | 0.52 (0.08,3.40) | 0.52 (0.09,2.91) | 0.64 (0.06,6.69) | **A+T+VKA** |  |
| 0.33 (0.02,6.07) | 1.12 (0.33,3.75) | 1.08 (0.06,18.83) | 1.21 (0.55,2.69) | 1.20 (0.88,1.66) | 1.48 (0.29,7.55) | 2.31 (0.43,12.55) | **A+C+VKA*** |

*regimens adjusted mainly for these confounding factors: age, sex, dyslipidemia, heart failure, renal impairment, history of stroke, indication of PCI, DES implantation

**Abbreviations:** A+C, aspirin + clopidogrel; A+C+LMWH, aspirin + clopidogrel + low-molecular weight heparin; A+C+VKA, aspirin + clopidogrel + vitamin K antagonist; A+P+VKA, aspirin + prasugrel + vitamin K antagonist; A+T+VKA, aspirin + ticagrelor + vitamin K antagonist; A+VKA, aspirin + vitamin K antagonist; C+VKA, clopidogrel + vitamin K antagonist; T+VKA, ticagrelor + vitamin K antagonist

Treatments are ordered by SUCRA rank. Comparisons between treatments should be read from column to row for each outcome (row treatment is reference)

## eTable 8.4 Results of network meta-analysis of treatment options on repeated revascularization

The following table shows the effect sizes (risk ratio) and the rank order (SUCRA ranks) compared to aspirin + clopidogrel + vitamin K antagonist

| **Treatment name** | **Risk ratio** | **95% Confidence interval** | **p-value** | **SUCRA rank** |
| --- | --- | --- | --- | --- |
| A+C+VKA* | *Reference* | | | 1 |
| C+VKA | 0.98 | 0.57, 1.66 | 0.927 | 2 |
| A+C* | 1.06 | 0.85, 1.34 | 0.600 | 3 |
| A+VKA | 1.68 | 0.99, 2.87 | 0.055 | 4 |
| **Number of studies** | **8** | **Number of studies with adjustment of confounding factors** | **2** |  |

## eFigure 8.4 Network estimated risk ratios (95% confidence intervals) of treatment options on repeated revascularization

| **C+VKA** |  |  |  |
| --- | --- | --- | --- |
| 0.92 (0.52,1.63) | **A+C*** |  |  |
| 0.58 (0.29,1.17) | 0.63 (0.35,1.13) | **A+VKA** |  |
| 0.98 (0.57,1.66) | 1.06 (0.85,1.34) | 1.68 (0.99,2.87) | **A+C+VKA*** |

*regimens adjusted mainly for these confounding factors: age, sex, dyslipidemia, heart failure, renal impairment, history of stroke, indication of PCI, DES implantation

**Abbreviations:** A+C, aspirin + clopidogrel; A+C+LMWH, aspirin + clopidogrel + low-molecular weight heparin; A+C+VKA, aspirin + clopidogrel + vitamin K antagonist; A+P+VKA, aspirin + prasugrel + vitamin K antagonist; A+T+VKA, aspirin + ticagrelor + vitamin K antagonist; A+VKA, aspirin + vitamin K antagonist; C+VKA, clopidogrel + vitamin K antagonist; T+VKA, ticagrelor + vitamin K antagonist

Treatments are ordered by SUCRA rank. Comparisons between treatments should be read from column to row for each outcome (row treatment is reference)

## eTable 8.5 Results of network meta-analysis of treatment options on stent thrombosis

The following table shows the effect sizes (risk ratio) and the rank order (SUCRA ranks) compared to aspirin + clopidogrel + vitamin K antagonist

| **Treatment name** | **Risk ratio** | **95% Confidence interval** | **p-value** | **SUCRA rank** |
| --- | --- | --- | --- | --- |
| A+C+VKA | *Reference* | | | 1 |
| A+C | 1.22 | 0.66, 2.26 | 0.519 | 2 |
| A+VKA | 1.30 | 0.38, 4.42 | 0.680 | 3 |
| T+VKA | 1.48 | 0.03, 73.85 | 0.845 | 4 |
| C+VKA | 2.00 | 0.45, 8.83 | 0.361 | 5 |
| A+P+VKA | 3.25 | 0.16, 65.56 | 0.443 | 6 |
| A+T+VKA | 9.26 | 0.87, 98.47 | 0.065 | 7 |
| **Number of studies** | **16** | **Number of studies with adjustment of confounding factors** | **0** |  |

**Abbreviations:** A+C, aspirin + clopidogrel; A+C+LMWH, aspirin + clopidogrel + low-molecular weight heparin; A+C+VKA, aspirin + clopidogrel + vitamin K antagonist; A+P+VKA, aspirin + prasugrel + vitamin K antagonist; A+T+VKA, aspirin + ticagrelor + vitamin K antagonist; A+VKA, aspirin + vitamin K antagonist; C+VKA, clopidogrel + vitamin K antagonist; T+VKA, ticagrelor + vitamin K antagonist

## eFigure 8.5 Network estimated risk ratios (95% confidence intervals) of treatment options on stent thrombosis

| **A+C** |  |  |  |  |  |  |
| --- | --- | --- | --- | --- | --- | --- |
| 0.94 (0.29,3.08) | **A+VKA** |  |  |  |  |  |
| 0.83 (0.02,43.47) | 0.88 (0.01,52.94) | **T+VKA** |  |  |  |  |
| 0.61 (0.13,2.88) | 0.65 (0.10,4.31) | 0.74 (0.01,48.51) | **C+VKA** |  |  |  |
| 0.38 (0.02,8.10) | 0.40 (0.02,10.26) | 0.45 (0.00,63.18) | 0.62 (0.02,17.61) | **A+P+VKA** |  |  |
| 0.13 (0.01,1.52) | 0.14 (0.01,2.01) | 0.16 (0.00,15.41) | 0.22 (0.01,3.52) | 0.35 (0.01,16.05) | **A+T+VKA** |  |
| 1.22 (0.66,2.26) | 1.30 (0.38,4.42) | 1.48 (0.03,73.85) | 2.00 (0.45,8.83) | 3.25 (0.16,65.56) | 9.26 (0.87,98.47) | **A+C+VKA** |

**Abbreviations:** A+C, aspirin + clopidogrel; A+C+LMWH, aspirin + clopidogrel + low-molecular weight heparin; A+C+VKA, aspirin + clopidogrel + vitamin K antagonist; A+P+VKA, aspirin + prasugrel + vitamin K antagonist; A+T+VKA, aspirin + ticagrelor + vitamin K antagonist; A+VKA, aspirin + vitamin K antagonist; C+VKA, clopidogrel + vitamin K antagonist; T+VKA, ticagrelor + vitamin K antagonist

Treatments are ordered by SUCRA rank. Comparisons between treatments should be read from column to row for each outcome (row treatment is reference)

## eTable 8.6 Results of network meta-analysis of treatment options on all-cause death

The following table shows the effect sizes (risk ratio) and the rank order (SUCRA ranks) compared to aspirin + clopidogrel + vitamin K antagonist

| **Treatment name** | **Risk ratio** | **95% Confidence interval** | **p-value** | **SUCRA rank** |
| --- | --- | --- | --- | --- |
| A+T+VKA | 0.20 | 0.01, 3.27 | 0.256 | 1 |
| C+VKA | 0.78 | 0.40, 1.50 | 0.451 | 2 |
| A+C+VKA* | *Reference* | | | 3 |
| A+VKA | 1.03 | 0.50, 2.12 | 0.943 | 4 |
| T+VKA | 1.18 | 0.32, 4.44 | 0.801 | 5 |
| A+C* | 1.15 | 0.97, 1.38 | 0.116 | 6 |
| A+P+VKA | 2.42 | 0.57, 10.23 | 0.229 | 7 |
| A+C+LMWH | 3.59 | 0.98, 13.10 | 0.053 | 8 |
| **Number of studies** | **22** | **Number of studies with adjustment of confounding factors** | **4** |  |

*regimens adjusted mainly for these confounding factors: age, sex, dyslipidemia, heart failure, renal impairment, history of stroke, indication of PCI, DES implantation

**Abbreviations:** A+C, aspirin + clopidogrel; A+C+LMWH, aspirin + clopidogrel + low-molecular weight heparin; A+C+VKA, aspirin + clopidogrel + vitamin K antagonist; A+P+VKA, aspirin + prasugrel + vitamin K antagonist; A+T+VKA, aspirin + ticagrelor + vitamin K antagonist; A+VKA, aspirin + vitamin K antagonist; C+VKA, clopidogrel + vitamin K antagonist; T+VKA, ticagrelor + vitamin K antagonist

## eFigure 8.6 Network estimated risk ratios (95% confidence intervals) of treatment options on all-cause death

| **A+T+VKA** |  |  |  |  |  |  |  |
| --- | --- | --- | --- | --- | --- | --- | --- |
| 0.25 (0.01,4.55) | **C+VKA** |  |  |  |  |  |  |
| 0.19 (0.01,3.49) | 0.76 (0.30,1.88) | **A+VKA** |  |  |  |  |  |
| 0.17 (0.01,3.70) | 0.65 (0.15,2.87) | 0.87 (0.19,3.91) | **T+VKA** |  |  |  |  |
| 0.17 (0.01,2.85) | 0.67 (0.34,1.33) | 0.89 (0.42,1.88) | 1.03 (0.27,3.89) | **A+C*** |  |  |  |
| 0.08 (0.00,1.91) | 0.32 (0.07,1.56) | 0.42 (0.08,2.13) | 0.49 (0.07,3.45) | 0.48 (0.11,2.03) | **A+P+VKA** |  |  |
| 0.05 (0.00,1.21) | **0.22 (0.05,0.92)** | 0.29 (0.06,1.26) | 0.33 (0.05,2.10) | 0.32 (0.09,1.17) | 0.67 (0.10,4.68) | **A+C+LMWH** |  |
| 0.20 (0.01,3.27) | 0.78 (0.40,1.50) | 1.03 (0.50,2.12) | 1.18 (0.32,4.44) | 1.15 (0.97,1.38) | 2.42 (0.57,10.23) | 3.59 (0.98,13.10) | **A+C+VKA*** |

*regimens adjusted mainly for these confounding factors: age, sex, dyslipidemia, heart failure, renal impairment, history of stroke, indication of PCI, DES implantation

**Abbreviations:** A+C, aspirin + clopidogrel; A+C+LMWH, aspirin + clopidogrel + low-molecular weight heparin; A+C+VKA, aspirin + clopidogrel + vitamin K antagonist; A+P+VKA, aspirin + prasugrel + vitamin K antagonist; A+T+VKA, aspirin + ticagrelor + vitamin K antagonist; A+VKA, aspirin + vitamin K antagonist; C+VKA, clopidogrel + vitamin K antagonist; T+VKA, ticagrelor + vitamin K antagonist

Treatments are ordered by SUCRA rank. Comparisons between treatments should be read from column to row for each outcome (row treatment is reference)

# Appendix 9

**Treatment ranking and surface under the cumulative ranking curves (SUCRA) for each outcome**

## eFigure 9.1 SUCRA ranking curve for major bleeding


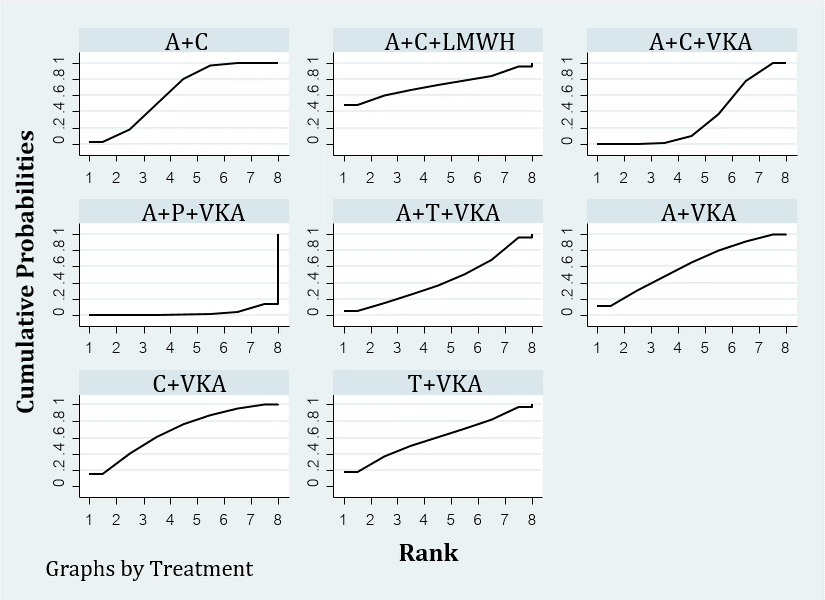


**Abbreviations:** A+C, aspirin + clopidogrel; A+C+LMWH, aspirin + clopidogrel + low-molecular weight heparin; A+C+VKA, aspirin + clopidogrel + vitamin K antagonist; A+P+VKA, aspirin + prasugrel + vitamin K antagonist; A+T+VKA, aspirin + ticagrelor + vitamin K antagonist; A+VKA, aspirin + vitamin K antagonist; C+VKA, clopidogrel + vitamin K antagonist; T+VKA, ticagrelor + vitamin K antagonist

## eFigure 9.2 SUCRA ranking curve for stroke and/or systemic embolism


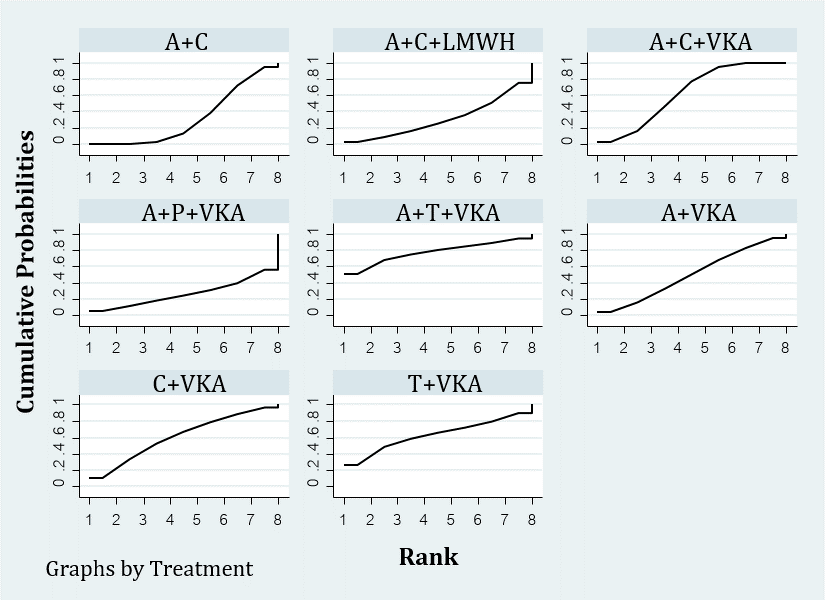


**Abbreviations:** A+C, aspirin + clopidogrel; A+C+LMWH, aspirin + clopidogrel + low-molecular weight heparin; A+C+VKA, aspirin + clopidogrel + vitamin K antagonist; A+P+VKA, aspirin + prasugrel + vitamin K antagonist; A+T+VKA, aspirin + ticagrelor + vitamin K antagonist; A+VKA, aspirin + vitamin K antagonist; C+VKA, clopidogrel + vitamin K antagonist; T+VKA, ticagrelor + vitamin K antagonist

## eFigure 9.3 SUCRA ranking curve for myocardial infarction


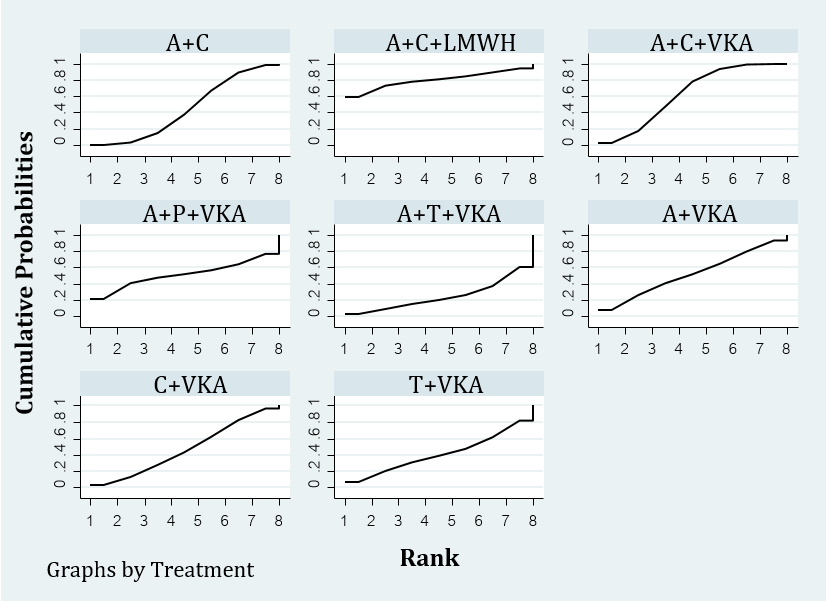


**Abbreviations:** A+C, aspirin + clopidogrel; A+C+LMWH, aspirin + clopidogrel + low-molecular weight heparin; A+C+VKA, aspirin + clopidogrel + vitamin K antagonist; A+P+VKA, aspirin + prasugrel + vitamin K antagonist; A+T+VKA, aspirin + ticagrelor + vitamin K antagonist; A+VKA, aspirin + vitamin K antagonist; C+VKA, clopidogrel + vitamin K antagonist; T+VKA, ticagrelor + vitamin K antagonist

## eFigure 9.4 SUCRA ranking curve for repeated revascularization


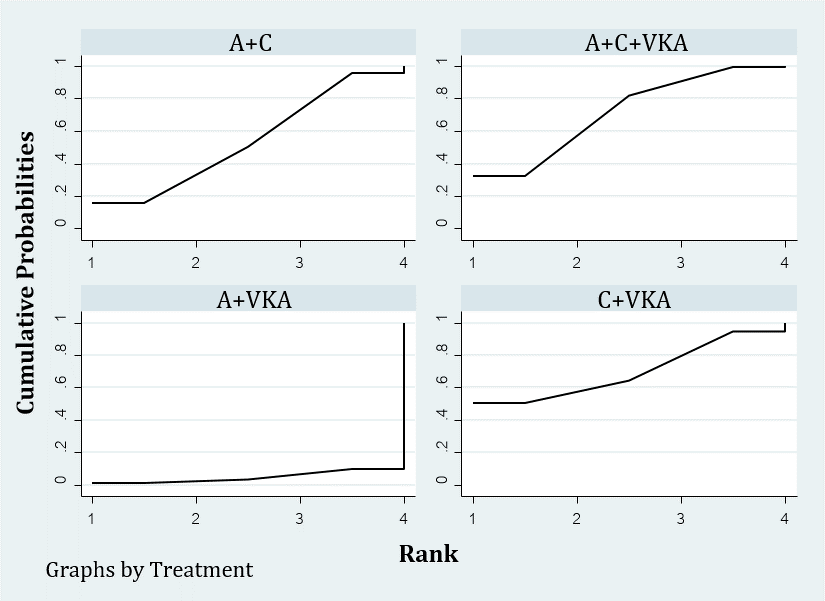


**Abbreviations:** A+C, aspirin + clopidogrel; A+C+VKA, aspirin + clopidogrel + vitamin K antagonist; A+VKA, aspirin + vitamin K antagonist; C+VKA, clopidogrel + vitamin K antagonist

## eFigure 9.5 SUCRA ranking curve for stent thrombosis


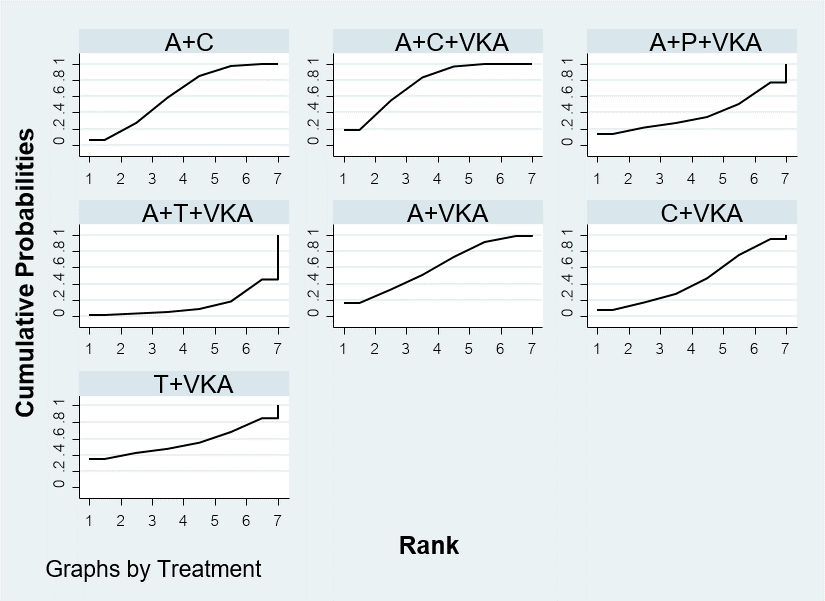


**Abbreviations:** A+C, aspirin + clopidogrel; A+C+VKA, aspirin + clopidogrel + vitamin K antagonist; A+P+VKA, aspirin + prasugrel + vitamin K antagonist; A+T+VKA, aspirin + ticagrelor + vitamin K antagonist; A+VKA, aspirin + vitamin K antagonist; C+VKA, clopidogrel + vitamin K antagonist; T+VKA, ticagrelor + vitamin K antagonist

## eFigure 9.6 SUCRA ranking curve for all-cause death


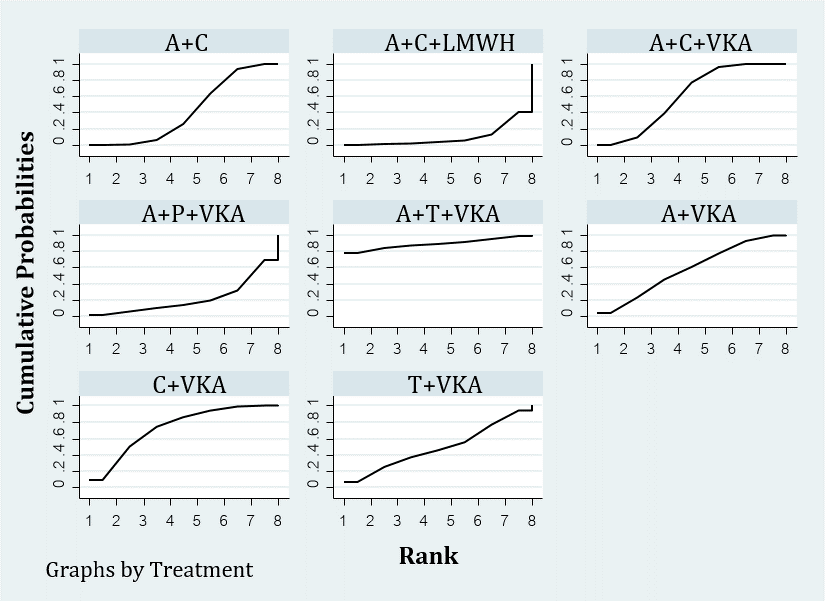


**Abbreviations:** A+C, aspirin + clopidogrel; A+C+LMWH, aspirin + clopidogrel + low-molecular weight heparin; A+C+VKA, aspirin + clopidogrel + vitamin K antagonist; A+P+VKA, aspirin + prasugrel + vitamin K antagonist; A+T+VKA, aspirin + ticagrelor + vitamin K antagonist; A+VKA, aspirin + vitamin K antagonist; C+VKA, clopidogrel + vitamin K antagonist; T+VKA, ticagrelor + vitamin K antagonist

# Appendix 10

**Analysis of re-classified regimens of RCTs**

## eTable 10.1 Analysis of re-classified regimens on major bleeding

The following table shows the effect sizes (risk ratio) and the rank order (SUCRA ranks) compared to aspirin + clopidogrel + vitamin K antagonist

| **Treatment name** | **Risk ratio** | **95% Confidence interval** | **p-value** | **SUCRA rank** |
| --- | --- | --- | --- | --- |
| VKA-DT | **0.51** | **0.30,0.87** | **0.014** | 1 |
| DOAC-DT | **0.68** | **0.49,0.94** | **0.020** | 2 |
| TT | reference | | | 3 |
| **Number of studies** | **3** |  |  |  |

**Abbreviations:** DOAC_DT, Direct-acting oral anticoagulant based dual therapy; TT, Triple therapy; VKA_DT, Vitamin K antagonist based dual therapy

## eFigure 10.1 Network estimated risk ratios (95% confidence intervals) of re-classified regimens on major bleeding

| **VKA_DT** |  |  |
| --- | --- | --- |
| 0.75 (0.40,1.41) | **DOAC_DT** |  |
| **0.51 (0.30,0.87)** | **0.68 (0.49,0.94)** | **TT** |

**Abbreviations:** DOAC_DT, Direct-acting oral anticoagulant based dual therapy; TT, Triple therapy; VKA_DT, Vitamin K antagonist based dual therapy

* Treatments are ordered by SUCRA rank. Comparisons between treatments should be read from column to row for each outcome (row treatment is reference)

## eTable 10.2 Analysis of re-classified regimens on stroke and/or systemic embolism

The following table shows the effect sizes (risk ratio) and the rank order (SUCRA ranks) compared to aspirin + clopidogrel + vitamin K antagonist

| **Treatment name** | **Risk ratio** | **95% Confidence interval** | **p-value** | **SUCRA rank** |
| --- | --- | --- | --- | --- |
| VKA-DT | 0.38 | 0.10,1.42 | 0.152 | 1 |
| TT | reference | | | 2 |
| DOAC-DT | 1.13 | 0.57,2.26 | 0.719 | 3 |
| **Number of studies** | **3** |  |  |  |

**Abbreviations:** DOAC_DT, Direct-acting oral anticoagulant based dual therapy; TT, Triple therapy; VKA_DT, Vitamin K antagonist based dual therapy

## eFigure 10.2 Network estimated risk ratios (95% confidence intervals) of re-classified regimens on stroke and/or systemic embolism

| **VKA_DT** |  |  |
| --- | --- | --- |
| 0.34 (0.08,1.49) | **DOAC_DT** |  |
| 0.38 (0.10,1.42) | 1.13 (0.57,2.26) | **TT** |

**Abbreviations:** DOAC_DT, Direct-acting oral anticoagulant based dual therapy; TT, Triple therapy; VKA_DT, Vitamin K antagonist based dual therapy

* Treatments are ordered by SUCRA rank. Comparisons between treatments should be read from column to row for each outcome (row treatment is reference)

## eTable 10.3 Analysis of re-classified regimens on myocardial infarction

The following table shows the effect sizes (risk ratio) and the rank order (SUCRA ranks) compared to aspirin + clopidogrel + vitamin K antagonist

| **Treatment name** | **Risk ratio** | **95% Confidence interval** | **p-value** | **SUCRA rank** |
| --- | --- | --- | --- | --- |
| VKA-DT | 0.70 | 0.31,1.62 | 0.411 | 1 |
| TT | reference | | | 2 |
| DOAC-DT | 1.05 | 0.69,1.58 | 0.824 | 3 |
| **Number of studies** | **3** |  |  |  |

**Abbreviations:** DOAC_DT, Direct-acting oral anticoagulant based dual therapy; TT, Triple therapy; VKA_DT, Vitamin K antagonist based dual therapy

## eFigure 10.3 Network estimated risk ratios (95% confidence intervals) of re-classified regimens on myocardial infarction

| **VKA_DT** |  |  |
| --- | --- | --- |
| 0.67 (0.27,1.70) | **DOAC_DT** |  |
| 0.70 (0.31,1.62) | 1.05 (0.69,1.58) | **TT** |

**Abbreviations:** DOAC_DT, Direct-acting oral anticoagulant based dual therapy; TT, Triple therapy; VKA_DT, Vitamin K antagonist based dual therapy

* Treatments are ordered by SUCRA rank. Comparisons between treatments should be read from column to row for each outcome (row treatment is reference)

## eTable 10.4 Analysis of re-classified regimens on stent thrombosis

The following table shows the effect sizes (risk ratio) and the rank order (SUCRA ranks) compared to aspirin + clopidogrel + vitamin K antagonist

| **Treatment name** | **Risk ratio** | **95% Confidence interval** | **p-value** | **SUCRA rank** |
| --- | --- | --- | --- | --- |
| VKA-DT | 0.45 | 0.14,1.45 | 0.183 | 1 |
| DOAC-DT | 1.09 | 0.48,2.47 | 0.833 | 2 |
| TT | reference | | | 3 |
| **Number of studies** | **3** |  |  |  |

**Abbreviations:** DOAC_DT, Direct-acting oral anticoagulant based dual therapy; TT, Triple therapy; VKA_DT, Vitamin K antagonist based dual therapy

## eFigure 10.4 Network estimated risk ratios (95% confidence intervals) of re-classified regimens on stent thrombosis

| **VKA_DT** |  |  |
| --- | --- | --- |
| 0.41 (0.10,1.72) | **DOAC_DT** |  |
| 0.45 (0.14,1.45) | 1.09 (0.48,2.47) | **TT** |

**Abbreviations:** DOAC_DT, Direct-acting oral anticoagulant based dual therapy; TT, Triple therapy; VKA_DT, Vitamin K antagonist based dual therapy

* Treatments are ordered by SUCRA rank. Comparisons between treatments should be read from column to row for each outcome (row treatment is reference)

## eTable 10.5 Analysis of re-classified regimens on all-cause death

The following table shows the effect sizes (risk ratio) and the rank order (SUCRA ranks) compared to aspirin + clopidogrel + vitamin K antagonist

| **Treatment name** | **Risk ratio** | **95% Confidence interval** | **p-value** | **SUCRA rank** |
| --- | --- | --- | --- | --- |
| VKA-DT | **0.40** | **0.17,0.93** | **0.035** | 1 |
| DOAC-DT | 0.98 | 0.59,1.61 | 0.927 | 2 |
| TT | reference | | | 3 |
| **Number of studies** | **3** |  |  |  |

**Abbreviations:** DOAC_DT, Direct-acting oral anticoagulant based dual therapy; TT, Triple therapy; VKA_DT, Vitamin K antagonist based dual therapy

## eFigure 10.5 Network estimated risk ratios (95% confidence intervals) of re-classified regimens on all-cause death

| **VKA_DT** |  |  |
| --- | --- | --- |
| 0.41 (0.15,1.09) | **DOAC_DT** |  |
| **0.40 (0.17,0.93)** | 0.98 (0.59,1.61) | **TT** |

**Abbreviations:** DOAC_DT, Direct-acting oral anticoagulant based dual therapy; TT, Triple therapy; VKA_DT, Vitamin K antagonist based dual therapy

* Treatments are ordered by SUCRA rank. Comparisons between treatments should be read from column to row for each outcome (row treatment is reference)

# Appendix 11

**Analysis of re-classified regimens of non-RCTs**

## eTable 11.1 Analysis of re-classified regimens on major bleeding outcome

The following table shows the effect sizes (risk ratio) and the rank order (SUCRA ranks) compared to aspirin + clopidogrel + vitamin K antagonist

| **Treatment name** | **Risk ratio** | **95% Confidence interval** | **p-value** | **SUCRA rank** |
| --- | --- | --- | --- | --- |
| DAPT | **0.59** | **0.41,0.85** | **0.004** | 1 |
| VKA-DT | 0.74 | 0.41,1.34 | 0.322 | 2 |
| TT | reference | | | 3 |
| newP2Y12TT | 2.12 | 0.76,6.00 | 0.155 | 4 |
| **Number of studies**  **(Number of studies with adjustment of confounding factors)** | **17**  **(4)** | **Overall inconsistency**  **chi2 (P value)** | **1.82**  **(P=0.4023)** |  |

**Abbreviations:** DAPT, Dual antiplatelet; TT, Triple therapy; newP2Y12TT, New P2Y_12_ inhibitor-based triple therapy; VKA_DT, Vitamin K antagonist based dual therapy

## eFigure 11.1 Network estimated risk ratios (95% confidence intervals) of re-classified regimens on major bleeding outcome

| **DAPT** |  |  |  |
| --- | --- | --- | --- |
| 0.80 (0.42,1.54) | **VKA-DT** |  |  |
| **0.28 (0.09,0.84)** | 0.35 (0.11,1.15) | **newP2Y12TT** |  |
| **0.59 (0.41,0.85)** | 0.74 (0.41,1.34) | 2.12 (0.75,6.00) | **TT** |

**Abbreviations:** DAPT, Dual antiplatelet; TT, Triple therapy; newP2Y12TT, New P2Y_12_ inhibitor-based triple therapy; VKA_DT, Vitamin K antagonist based dual therapy

* Treatments are ordered by SUCRA rank. Comparisons between treatments should be read from column to row for each outcome (row treatment is reference)

## eTable 11.2 Analysis of re-classified regimens on stroke and/ or systemic embolism

The following table shows the effect sizes (risk ratio) and the rank order (SUCRA ranks) compared to aspirin + clopidogrel + vitamin K antagonist

| **Treatment name** | **Risk ratio** | **95% Confidence interval** | **p-value** | **SUCRA rank** |
| --- | --- | --- | --- | --- |
| TT | reference | | | 1 |
| VKA-DT | 1.05 | 0.48, 2.33 | 0.900 | 2 |
| newP2Y12TT | 1.30 | 0.21, 8.22 | 0.779 | 3 |
| DAPT | **1.65** | **1.08, 2.51** | **0.020** | 4 |
| **Number of studies**  **(Number of studies with adjustment of confounding factors)** | **22**  **(3)** | **Overall inconsistency**  **chi2 (P value)** | **3.57**  **(P=0.1677)** |  |

**Abbreviations:** DAPT, Dual antiplatelet; TT, Triple therapy; newP2Y12TT, New P2Y_12_ inhibitor-based triple therapy; VKA_DT, Vitamin K antagonist based dual therapy

## eFigure 11.2 Network estimated risk ratios (95% confidence intervals) of re-classified regimens on stroke and/ or systemic embolism

| **VKA-DT** |  |  |  |
| --- | --- | --- | --- |
| 0.81 (0.11,6.00) | **newP2Y12TT** |  |  |
| 0.64 (0.28,1.44) | 0.79 (0.12,5.22) | **DAPT** |  |
| 1.05 (0.48,2.33) | 1.30 (0.21,8.22) | **1.65 (1.08,2.51)** | **TT** |

**Abbreviations:** DAPT, Dual antiplatelet; TT, Triple therapy; newP2Y12TT, New P2Y_12_ inhibitor-based triple therapy; VKA_DT, Vitamin K antagonist based dual therapy

* Treatments are ordered by SUCRA rank. Comparisons between treatments should be read from column to row for each outcome (row treatment is reference)

## eTable 11.3 Analysis of re-classified regimens on myocardial infarction

The following table shows the effect sizes (risk ratio) and the rank order (SUCRA ranks) compared to aspirin + clopidogrel + vitamin K antagonist

| **Treatment name** | **Risk ratio** | **95% Confidence interval** | **p-value** | **SUCRA rank** |
| --- | --- | --- | --- | --- |
| TT | reference | | | 1 |
| DAPT | 1.19 | 0.88,1.60 | 0.265 | 2 |
| VKA-DT | 1.23 | 0.75,2.02 | 0.418 | 3 |
| newP2Y12TT | 1.90 | 0.45,8.05 | 0.381 | 4 |
| **Number of studies**  **(Number of studies with adjustment of confounding factors)** | **20**  **(3)** | **Overall inconsistency**  **chi2 (P value)** | **0.46**  **(P=0.7945)** |  |

**Abbreviations:** DAPT, Dual antiplatelet; TT, Triple therapy; newP2Y12TT, New P2Y_12_ inhibitor-based triple therapy; VKA_DT, Vitamin K antagonist based dual therapy

## eFigure 11.3 Network estimated risk ratios (95% confidence intervals) of re-classified regimens on myocardial infarction

| **DAPT** |  |  |  |
| --- | --- | --- | --- |
| 0.97 (0.58,1.62) | **VKA-DT** |  |  |
| 0.62 (0.14,2.72) | 0.65 (0.14,2.97) | **newP2Y12TT** |  |
| 1.19 (0.88,1.60) | 1.23 (0.75,2.02) | 1.90 (0.45,8.05) | **TT** |

**Abbreviations:** DAPT, Dual antiplatelet; TT, Triple therapy; newP2Y12TT, New P2Y_12_ inhibitor-based triple therapy; VKA_DT, Vitamin K antagonist based dual therapy

* Treatments are ordered by SUCRA rank. Comparisons between treatments should be read from column to row for each outcome (row treatment is reference)

## eTable 11.4 Analysis of re-classified regimens on repeated revascularization

The following table shows the effect sizes (risk ratio) and the rank order (SUCRA ranks) compared to aspirin + clopidogrel + vitamin K antagonist

| **Treatment name** | **Risk ratio** | **95% Confidence interval** | **p-value** | **SUCRA rank** |
| --- | --- | --- | --- | --- |
| TT | reference | | | 1 |
| DAPT | 1.07 | 0.85,1.34 | 0.556 | 2 |
| VKA-DT | 1.24 | 0.88,1.77 | 0.224 | 3 |
| **Number of studies**  **(Number of studies with adjustment of confounding factors)** | **8**  **(2)** | **Overall inconsistency**  **chi2 (P value)** | **0.24**  **(P=0.8875)** |  |

**Abbreviations:** DAPT, Dual antiplatelet; TT, Triple therapy; newP2Y12TT, New P2Y_12_ inhibitor-based triple therapy; VKA_DT, Vitamin K antagonist based dual therapy

## eFigure 11.4 Network estimated risk ratios (95% confidence intervals) of re-classified regimens on repeated revascularization

| **DAPT** |  |  |
| --- | --- | --- |
| 0.86 (0.58,1.28) | **VKA-DT** |  |
| 1.07 (0.85,1.34) | 1.24 (0.88,1.77) | **TT** |

**Abbreviations:** DAPT, Dual antiplatelet; TT, Triple therapy; newP2Y12TT, New P2Y_12_ inhibitor-based triple therapy; VKA_DT, Vitamin K antagonist based dual therapy

* Treatments are ordered by SUCRA rank. Comparisons between treatments should be read from column to row for each outcome (row treatment is reference)

## eTable 11.5 Analysis of re-classified regimens on stent thrombosis

The following table shows the effect sizes (risk ratio) and the rank order (SUCRA ranks) compared to aspirin + clopidogrel + vitamin K antagonist

| **Treatment name** | **Risk ratio** | **95% Confidence interval** | **p-value** | **SUCRA rank** |
| --- | --- | --- | --- | --- |
| TT | reference | | | 1 |
| DAPT | 1.28 | 0.71,2.31 | 0.409 | 2 |
| VKA-DT | 1.89 | 1.00,3.56 | 0.049 | 3 |
| newP2Y12TT | 6.20 | 0.97,39.77 | 0.054 | 4 |
| **Number of studies**  **(Number of studies with adjustment of confounding factors)** | **16**  **(0)** | **Overall inconsistency**  **chi2 (P value)** | **0.72**  **(P=0.6983)** |  |

**Abbreviations:** DAPT, Dual antiplatelet; TT, Triple therapy; newP2Y12TT, New P2Y_12_ inhibitor-based triple therapy; VKA_DT, Vitamin K antagonist based dual therapy

## eFigure 11.5 Network estimated risk ratios (95% confidence intervals) of re-classified regimens on stent thrombosis

| **DAPT** |  |  |  |
| --- | --- | --- | --- |
| 0.68 (0.33,1.39) | **VKA-DT** |  |  |
| 0.21 (0.03,1.45) | 0.30 (0.04,2.17) | **newP2Y12TT** |  |
| 1.28 (0.71,2.31) | **1.89 (1.00,3.56)** | 6.20 (0.97,39.77) | **TT** |

**Abbreviations:** DAPT, Dual antiplatelet; TT, Triple therapy; newP2Y12TT, New P2Y_12_ inhibitor-based triple therapy; VKA_DT, Vitamin K antagonist based dual therapy

* Treatments are ordered by SUCRA rank. Comparisons between treatments should be read from column to row for each outcome (row treatment is reference)

## eTable 11.6 Analysis of re-classified regimens on all-cause death

The following table shows the effect sizes (risk ratio) and the rank order (SUCRA ranks) compared to aspirin + clopidogrel + vitamin K antagonist

| **Treatment name** | **Risk ratio** | **95% Confidence interval** | **p-value** | **SUCRA rank** |
| --- | --- | --- | --- | --- |
| VKA-DT | 0.94 | 0.63,1.43 | 0.787 | 1 |
| TT | reference | | | 2 |
| newP2Y12TT | 1.41 | 0.37,5.28 | 0.614 | 3 |
| DAPT | 1.17 | 0.94,1.46 | 0.163 | 4 |
| **Number of studies**  **(Number of studies with adjustment of confounding factors)** | **22**  **(4)** | **Overall inconsistency**  **chi2 (P value)** | **0.16**  **(P=0.9213)** |  |

**Abbreviations:** DAPT, Dual antiplatelet; TT, Triple therapy; newP2Y12TT, New P2Y_12_ inhibitor-based triple therapy; VKA_DT, Vitamin K antagonist based dual therapy

## eFigure 11.6 Network estimated risk ratios (95% confidence intervals) of re-classified regimens on all-cause death

| **VKA-DT** |  |  |  |
| --- | --- | --- | --- |
| 0.67 (0.17,2.69) | **newP2Y12TT** |  |  |
| 0.81 (0.52,1.26) | 1.20 (0.31,4.60) | **DAPT** |  |
| 0.94 (0.63,1.43) | 1.41 (0.37,5.28) | 1.17 (0.94,1.46) | **TT** |

**Abbreviations:** DAPT, Dual antiplatelet; TT, Triple therapy; newP2Y12TT, New P2Y_12_ inhibitor-based triple therapy; VKA_DT, Vitamin K antagonist based dual therapy

* Treatments are ordered by SUCRA rank. Comparisons between treatments should be read from column to row for each outcome (row treatment is reference)

# Appendix 12

**Subgroup analyses**

## eTable 12.1 Subgroup analyses for the risk of major bleeding with treatment options in different groups of population

The following table shows the effect sizes (risk ratio) and the rank order (SUCRA ranks) compared to A+C+VKA before (main analysis) and after subgroup analyses.

| **Treatment name** | **Main analysis** | **SUCRA rank** | **Atrial fibrillation** | **SUCRA rank** | **AF predominant** | **SUCRA rank** | **ACS predominant** | **SUCRA rank** | **SCAD predominant** | **SUCRA rank** | **DES predominant** | **SUCRA rank** | **BMS predominant** | **SUCRA rank** |
| --- | --- | --- | --- | --- | --- | --- | --- | --- | --- | --- | --- | --- | --- | --- |
| A+C+LMWH | 0.29 (0.01,6.52) | 1 | 0.29 (0.01,6.40) | 1 | 0.29 (0.01,6.52) | 1 | 0.27 (0.01,6.06) | 1 | - | - | - | - | - | - |
| C+VKA | 0.48 (0.14,1.61) | 2 | 0.48 (0.14,1.59) | 2 | 0.48 (0.14,1.61) | 2 | 0.46 (0.14,1.55) | 2 | - | - | - | - | 0.49 (0.14,1.67) | 1 |
| A+C | **0.57 (0.39,0.84)** | 3 | **0.56 (0.37,0.86)** | 3 | **0.57 (0.39,0.84)** | 3 | **0.52 (0.35,0.80)** | 3 | 0.61 (0.00,195.88) | 3 | 0.48 (0.23,1.00) | 1 | 0.59 (0.33,1.06) | 2 |
| A+VKA | 0.58 (0.16,2.05) | 4 | - | - | 0.58 (0.16,2.05) | 4 | - | - | 0.58 (0.00,85.35) | 4 | 0.58 (0.12,2.72) | 2 | - | - |
| T+VKA | 0.59 (0.09,3.84) | 5 | - | - | 0.59 (0.09,3.84) | 5 | 0.59 (0.09,3.82) | 4 | - | - | - | - | 0.59 (0.09,3.85) | 3 |
| A+T+VKA | 0.93 (0.21,4.12) | 6 | - | - | - | - | 0.93 (0.21,4.10) | 5 | - | - | 0.93 (0.16,5.27) | 3 | - | - |
| A+C+VKA | reference | 7 | reference | 4 | reference | 6 | reference | 6 | reference | 2 | reference | 4 | reference | 4 |
| A+P+VKA | **5.09 (1.10,23.44)** | 8 | - | - | **5.09 (1.10,23.44)** | 7 | - | - | 5.09 (0.10,255.43) | 1 | 5.09 (0.87,29.82) | 5 | - | - |
| Overall inconsistency  chi2 (P value) | 3.06  (P=0.217) |  | 3.54  (P=0.170) |  | 3.06  (p=0.217) |  | 4.10  (P=0.129) |  | 0.05  (P=0.831) |  | 0.47  (P=0.491) |  | 5.31  (P=0.021) |  |
| Number of  studies | 17 |  | 9 |  | 16 |  | 13 |  | 3 |  | 8 |  | 7 |  |

**Abbreviations:** A+C, aspirin + clopidogrel; A+C+LMWH, aspirin + clopidogrel + low-molecular weight heparin; A+C+VKA, aspirin + clopidogrel + vitamin K antagonist; A+P+VKA, aspirin + prasugrel + vitamin K antagonist; A+T+VKA, aspirin + ticagrelor + vitamin K antagonist; A+VKA, aspirin + vitamin K antagonist; C+VKA, clopidogrel + vitamin K antagonist; T+VKA, ticagrelor + vitamin K antagonist; AF, atrial fibrillation; ACS, acute coronary syndrome; SCAD, stable coronary artery disease; DES, drug-eluting stent; BMS, bare-metal stent

## eTable 12.2 Subgroup analyses for the risk of major bleeding with treatment options based on study characteristics

The following table shows the effect sizes (risk ratio) and the rank order (SUCRA ranks) compared to A+C+VKA before (main analysis) and after subgroup analyses.

| **Treatment name** | **Main analysis** | **SUCRA rank** | **< 1 year follow-up** | **SUCRA rank** | **1 year follow-up** | **SUCRA rank** | **> 1 year follow-up** | **SUCRA rank** | **European only** | **SUCRA rank** | **Exclude Asia** | **SUCRA rank** | **Prospective design** | **SUCRA rank** | **Retrospective design** | **SUCRA rank** |
| --- | --- | --- | --- | --- | --- | --- | --- | --- | --- | --- | --- | --- | --- | --- | --- | --- |
| A+C+LMWH | 0.29 (0.01,6.52) | 1 | - | - | - | - | 0.27 (0.01,6.45) | 1 | 0.33 (0.01,7.67) | 1 | 0.34 (0.02,7.23) | 1 | - | - | 0.29 (0.01,6.24) | 1 |
| C+VKA | 0.48 (0.14,1.61) | 2 | - | - | 0.49 (0.13,1.78) | 1 | - | - | 0.52 (0.15,1.80) | 2 | 0.53 (0.17,1.60) | 2 | 0.48 (0.12,1.86) | 1 | - | - |
| A+C | **0.57 (0.39,0.84)** | 3 | 0.79 (0.19,3.24) | 3 | 0.59 (0.30,1.16) | 2 | **0.51 (0.27,0.95)** | 2 | 0.68 (0.40,1.17) | 5 | 0.69 (0.47,1.00) | 5 | 0.58 (0.33,1.03) | 2 | **0.57 (0.34,0.95)** | 2 |
| A+VKA | 0.58 (0.16,2.05) | 4 | 0.58 (0.20,1.69) | 1 | - | - | - | - | 0.58 (0.16,2.12) | 3 | 0.58 (0.19,1.82) | 3 | - | - | - | - |
| T+VKA | 0.59 (0.09,3.84) | 5 | 0.59 (0.10,3.38) | 2 | - | - | - | - | 0.59 (0.09,3.92) | 4 | 0.59 (0.10,3.54) | 4 | - | - | 0.59 (0.10,3.50) | 3 |
| A+T+VKA | 0.93 (0.21,4.12) | 6 | - | - | 0.93 (0.19,4.42) | 3 | - | - | - | - | - | - | - | - | - | - |
| A+C+VKA | reference | 7 | reference | 4 | reference | 4 | reference | 3 | reference | 6 | reference | 6 | reference | 3 | reference | 4 |
| A+P+VKA | **5.09 (1.10,23.44)** | 8 | **5.09 (1.29,20.01)** | 5 | - | - | - | - | **5.09 (1.08,24.04)** | 7 | **5.09 (1.22,21.22)** | 7 | 5.09 (0.96,27.00) | 4 | - | - |
| Overall inconsistency  chi2 (P value) | 3.06  (P=0.217) |  | 0.35  (P=0.556) |  | 8.81  (p=0.003) |  | 0.05  (P=0.832) |  | 1.89  (P=0.390) |  | 2.75  (P=0.253) |  | 2.96  (P=0.085) |  | 0.02  (P=0.876) |  |
| Number of  studies | 17 |  | 5 |  | 6 |  | 6 |  | 11 |  | 13 |  | 9 |  | 6 |  |

**Abbreviations:** A+C, aspirin + clopidogrel; A+C+LMWH, aspirin + clopidogrel + low-molecular weight heparin; A+C+VKA, aspirin + clopidogrel + vitamin K antagonist; A+P+VKA, aspirin + prasugrel + vitamin K antagonist; A+T+VKA, aspirin + ticagrelor + vitamin K antagonist; A+VKA, aspirin + vitamin K antagonist; C+VKA, clopidogrel + vitamin K antagonist; T+VKA, ticagrelor + vitamin K antagonist

# Appendix 13

**Sensitivity analyses**

## eTable 13.1 Sensitivity analyses for the risk of major bleeding with treatment options in the variety of major bleeding definitions

The following table shows the effect sizes (risk ratio) and the rank order (SUCRA ranks) compared to A+C+VKA before (standard analysis) and after sensitivity analyses.

| **Treatment name** | **Main analysis** | **SUCRA rank** | **TIMI (major)-based definition** | **SUCRA rank** | **GUSTO (severe)-based definition** | **SUCRA rank** | **ISTH (major)- based definition** | **SUCRA rank** |
| --- | --- | --- | --- | --- | --- | --- | --- | --- |
| A+C+LMWH | 0.29 (0.01,6.52) | 1 | - | - | - | - | 0.35 (0.01,16.94) | 2 |
| C+VKA | 0.48 (0.14,1.61) | 2 | 0.18 (0.02,1.72) | 1 | - | - | 0.25 (0.03,1.84) | 1 |
| A+C | **0.57 (0.39,0.84)** | 3 | 0.74 (0.39,1.44) | 3 | - | - | 0.72 (0.04,14.47) | 4 |
| A+VKA | 0.58 (0.16,2.05) | 4 | 0.43 (0.07,2.73) | 2 | - | - | 0.51 (0.15,1.72) | 3 |
| T+VKA | 0.59 (0.09,3.84) | 5 | 0.74 (0.05,11.00) | 4 | - | - | 0.74 (0.07,8.17) | 5 |
| A+T+VKA | 0.93 (0.21,4.12) | 6 | 1.39 (0.24,8.01) | 6 | - | - | 1.39 (0.40,4.85) | 6 |
| A+C+VKA | reference | 7 | reference | 5 | - | - | reference | 7 |
| A+P+VKA | **5.09 (1.10,23.44)** | 8 | 5.09 (0.89,29.15) | 7 | - | - | **5.09 (1.47,17.59)** | 8 |
| Overall inconsistency  chi2 (P value) | 3.06  (P=0.217) |  | 2.41  (P=0.121) |  |  |  | 8.97  (P=0.011) |  |
| Number of  studies | 17 |  | 10 |  | - | - | 14 |  |

**Abbreviations:** A+C, aspirin + clopidogrel; A+C+LMWH, aspirin + clopidogrel + low-molecular weight heparin; A+C+VKA, aspirin + clopidogrel + vitamin K antagonist; A+P+VKA, aspirin + prasugrel + vitamin K antagonist; A+T+VKA, aspirin + ticagrelor + vitamin K antagonist; A+VKA, aspirin + vitamin K antagonist; C+VKA, clopidogrel + vitamin K antagonist; T+VKA, ticagrelor + vitamin K antagonist; TIMI, Thrombolysis in Myocardial Infarction; GUSTO, Global Use of Strategies to Open Occluded Arteries ; ISTH, International Society of Thrombosis and Hemostasis

## eFigure 13.1 Network estimated risk ratios (95% confidence intervals) of treatment options of major bleeding according to TIMI (major) definition

| **C+VKA** |  |  |  |  |  |  |
| --- | --- | --- | --- | --- | --- | --- |
| 0.41 (0.02,7.58) | **A+VKA** |  |  |  |  |  |
| 0.24 (0.02,2.32) | 0.58 (0.08,4.12) | **A+C** |  |  |  |  |
| 0.24 (0.01,8.14) | 0.59 (0.02,15.36) | 1.00 (0.06,16.14) | **T+VKA** |  |  |  |
| 0.13 (0.01,2.25) | 0.31 (0.02,3.96) | 0.54 (0.08,3.48) | 0.53 (0.02,13.30) | **A+T+VKA** |  |  |
| **0.03 (0.00,0.61)** | 0.09 (0.01,1.08) | **0.15 (0.02,0.95)** | 0.15 (0.01,3.62) | 0.27 (0.02,3.24) | **A+P+VKA** |  |
| 0.18 (0.02,1.72) | 0.43 (0.07,2.73) | 0.74 (0.39,1.44) | 0.74 (0.05,11.00) | 1.39 (0.24,8.01) | 5.09 (0.89,29.15) | **A+C+VKA** |

**Abbreviations:** A+C, aspirin + clopidogrel; A+C+LMWH, aspirin + clopidogrel + low-molecular weight heparin; A+C+VKA, aspirin + clopidogrel + vitamin K antagonist; A+P+VKA, aspirin + prasugrel + vitamin K antagonist; A+T+VKA, aspirin + ticagrelor + vitamin K antagonist; A+VKA, aspirin + vitamin K antagonist; C+VKA, clopidogrel + vitamin K antagonist; T+VKA, ticagrelor + vitamin K antagonist; TIMI, Thrombolysis in Myocardial Infarction

* Treatments are ordered by SUCRA rank. Comparisons between treatments should be read from column to row for each outcome (row treatment is reference)

## eFigure 13.2 Network estimated risk ratios (95% confidence intervals) of treatment options of major bleeding according to ISTH (major) definition

| **C+VKA** |  |  |  |  |  |  |  |
| --- | --- | --- | --- | --- | --- | --- | --- |
| 0.33 (0.01,12.55) | **A+C+LMWH** |  |  |  |  |  |  |
| 0.50 (0.05,5.10) | 0.69 (0.01,40.16) | **A+VKA** |  |  |  |  |  |
| 0.16 (0.02,1.21) | 0.48 (0.02,9.97) | 0.71 (0.03,17.97) | **A+C** |  |  |  |  |
| 0.34 (0.02,7.70) | 0.47 (0.00,45.34) | 0.69 (0.05,10.17) | 0.97 (0.02,45.43) | **T+VKA** |  |  |  |
| 0.18 (0.02,1.90) | 0.25 (0.00,14.85) | 0.37 (0.06,2.10) | 0.52 (0.02,13.38) | 0.53 (0.04,7.99) | **A+T+VKA** |  |  |
| **0.05 (0.00,0.52)** | 0.07 (0.00,4.05) | **0.10 (0.02,0.57)** | 0.14 (0.01,3.64) | 0.15 (0.01,2.17) | 0.27 (0.05,1.59) | **A+P+VKA** |  |
| 0.25 (0.03,1.84) | 0.35 (0.01,16.94) | 0.51 (0.15,1.72) | 0.72 (0.04,14.47) | 0.74 (0.07,8.17) | 1.39 (0.40,4.85) | **5.08 (1.47,17.59)** | **A+C+VKA** |

**Abbreviations:** A+C, aspirin + clopidogrel; A+C+LMWH, aspirin + clopidogrel + low-molecular weight heparin; A+C+VKA, aspirin + clopidogrel + vitamin K antagonist; A+P+VKA, aspirin + prasugrel + vitamin K antagonist; A+T+VKA, aspirin + ticagrelor + vitamin K antagonist; A+VKA, aspirin + vitamin K antagonist; C+VKA, clopidogrel + vitamin K antagonist; T+VKA, ticagrelor + vitamin K antagonist; ISTH, International Society of Thrombosis and Hemostasis

*Treatments are ordered by SUCRA rank. Comparisons between treatments should be read from column to row for each outcome (row treatment is reference)

## eTable 13.2 Sensitivity analyses for the risk of major bleeding with treatment options of different study characteristics

The following table shows the effect sizes (risk ratio) and the rank order (SUCRA ranks) compared to A+C+VKA before (standard analysis) and after sensitivity analyses.

| **Treatment name** | **Main analysis** | **SUCRA rank** | **Inclusion only studies with adjusted analysis** | **SUCRA rank** | **Omitting small sample size studies (>25^th^ percentile)** | **SUCRA rank** | **Omitting serious-to-critical risk of ROBs** | **SUCRA rank** | **Inclusion of multicentre only** | **SUCRA rank** |
| --- | --- | --- | --- | --- | --- | --- | --- | --- | --- | --- |
| A+C+LMWH | 0.29 (0.01,6.52) | 1 | - | - | - | - | - | - | - | - |
| C+VKA | 0.48 (0.14,1.61) | 2 | - | - | 0.51 (0.16,1.58) | 1 | - | - | 0.45 (0.13,1.56) | 1 |
| A+C | **0.57 (0.39,0.84)** | 3 | **0.36**  **(0.22,0.60)** | 1 | **0.63 (0.44,0.91)** | 2 | 0.56 (0.17,1.84) | 1 | **0.50 (0.30,0.83)** | 2 |
| A+VKA | 0.58 (0.16,2.05) | 4 | - | - | 0.58 (0.18,1.89) | 3 | 0.58 (0.08,4.28) | 2 | 0.58 (0.16,2.11) | 3 |
| T+VKA | 0.59 (0.09,3.84) | 5 | - | - | - | - | - | - | 0.59 (0.09,3.91) | 4 |
| A+T+VKA | 0.93 (0.21,4.12) | 6 | - | - | - | - | - | - | - | - |
| A+C+VKA | reference | 7 | reference | 2 | reference | 4 | reference | 3 | reference | 5 |
| A+P+VKA | **5.09 (1.10,23.44)** | 8 | - | - | **5.09 (1.18,21.86)** | 5 | - | - | **5.09 (1.08,23.97)** | 6 |
| Overall inconsistency  chi2 (P value) | 3.06  (P=0.217) |  | 15.39  (P=0.001) |  | 3.28  (P=0.070) |  | 0.28  (P=0.594) |  | 8.13  (P=0.004) |  |
| Number of  studies | 17 |  | 4 |  | 13 |  | 4 |  | 11 |  |

**Abbreviations:** A+C, aspirin + clopidogrel; A+C+LMWH, aspirin + clopidogrel + low-molecular weight heparin; A+C+VKA, aspirin + clopidogrel + vitamin K antagonist; A+P+VKA, aspirin + prasugrel + vitamin K antagonist; A+T+VKA, aspirin + ticagrelor + vitamin K antagonist; A+VKA, aspirin + vitamin K antagonist; C+VKA, clopidogrel + vitamin K antagonist; T+VKA, ticagrelor + vitamin K antagonist; ROB, Risk of bias

## eFigure 13.3 Network estimated risk ratios (95% confidence intervals) of treatment options on major bleeding by omitting studies with small sample size (less than 25^th^ percentile of populations)

| **C+VKA** |  |  |  |  |
| --- | --- | --- | --- | --- |
| 0.81 (0.26,2.52) | **A+C** |  |  |  |
| 0.87 (0.17,4.47) | 1.08 (0.32,3.70) | **A+VKA** |  |  |
| **0.10 (0.02,0.63)** | **0.12 (0.03,0.56)** | **0.11 (0.02,0.74)** | **A+P+VKA** |  |
| 0.51 (0.16,1.58) | **0.63 (0.44,0.91)** | 0.58 (0.18,1.89) | **5.09 (1.18,21.86)** | **A+C+VKA** |

**Abbreviations:** A+C, aspirin + clopidogrel; A+C+VKA, aspirin + clopidogrel + vitamin K antagonist; A+P+VKA, aspirin + prasugrel + vitamin K antagonist; A+VKA, aspirin + vitamin K antagonist; C+VKA, clopidogrel + vitamin K antagonist

* Treatments are ordered by SUCRA rank. Comparisons between treatments should be read from column to row for each outcome (row treatment is reference)

## eFigure 13.4 Network estimated risk ratios (95% confidence intervals) of treatment options on major bleeding by omitting studies with serious-to-critical risk of ROBs

| **A+C** |  |  |
| --- | --- | --- |
| 0.96 (0.09,9.81) | **A+VKA** |  |
| 0.56 (0.17,1.84) | 0.58 (0.08,4.28) | **A+C+VKA** |

**Abbreviations:** A+C, aspirin + clopidogrel; A+C+VKA, aspirin + clopidogrel + vitamin K antagonist; A+VKA, aspirin + vitamin K antagonist

* Treatments are ordered by SUCRA rank. Comparisons between treatments should be read from column to row for each outcome (row treatment is reference)

## eFigure 13.5 Network estimated risk ratios (95% confidence intervals) of treatment options on major bleeding by inclusion of multicenter studies

| **C+VKA** |  |  |  |  |  |
| --- | --- | --- | --- | --- | --- |
| 0.55 (0.21,1.47) | **A+C** |  |  |  |  |
| 1.16 (0.32,4.17) | 0.77 (0.29,2.07) | **A+VKA** |  |  |  |
| 1.14 (0.17,7.46) | 0.76 (0.14,4.13) | 0.98 (0.15,6.37) | **T+VKA** |  |  |
| **0.13 (0.03,0.62)** | **0.09 (0.02,0.33)** | **0.11 (0.02,0.53)** | **0.12 (0.01,0.91)** | **A+P+VKA** |  |
| 0.67 (0.27,1.68) | **0.45 (0.29,0.68)** | 0.58 (0.24,1.42) | 0.59 (0.11,3.06) | **5.08 (1.47,17.59)** | **A+C+VKA** |

**Abbreviations:** A+C, aspirin + clopidogrel; A+C+VKA, aspirin + clopidogrel + vitamin K antagonist; A+P+VKA, aspirin + prasugrel + vitamin K antagonist; A+VKA, aspirin + vitamin K antagonist; C+VKA, clopidogrel + vitamin K antagonist; T+VKA, ticagrelor + vitamin K antagonist

* Treatments are ordered by SUCRA rank. Comparisons between treatments should be read from column to row for each outcome (row treatment is reference)

## eTable 13.3 Sensitivity analysis of type of stroke: ischemic stroke

The following table shows the effect sizes (risk ratio) and the rank order (SUCRA ranks) compared to aspirin + clopidogrel + vitamin K antagonist

| **Treatment name** | **Risk ratio** | **95% Confidence interval** | **p-value** | **SUCRA rank** |
| --- | --- | --- | --- | --- |
| A+VKA | 0.20 | 0.01,4.15 | 0.298 | 1 |
| A+T+VKA | 0.35 | 0.02,5.97 | 0.465 | 2 |
| A+C+VKA | Reference | | | 3 |
| A+C | 1.03 | 0.79,1.33 | 0.843 | 4 |
| A+P+VKA | 1.48 | 0.08,25.73 | 0.790 | 5 |
| **Number of studies** | **9** | **Overall inconsistency**  **chi2 (P value)** | **1.08**  **(P=0.298)** |  |

**Abbreviations:** A+C, aspirin + clopidogrel; A+C+VKA, aspirin + clopidogrel + vitamin K antagonist; A+P+VKA, aspirin + prasugrel + vitamin K antagonist; A+T+VKA, aspirin + ticagrelor + vitamin K antagonist; A+VKA, aspirin + vitamin K antagonist

## eFigure 13.6 Network estimated risk ratios (95% confidence intervals) of ischemic stroke

| **A+VKA** |  |  |  |  |
| --- | --- | --- | --- | --- |
| 0.58 (0.01,37.00) | **A+T+VKA** |  |  |  |
| 0.19 (0.01,4.09) | 0.34 (0.02,5.88) | **A+C** |  |  |
| 0.14 (0.00,8.78) | 0.23 (0.00,13.30) | 0.70 (0.04,12.32) | **A+P+VKA** |  |
| 0.20 (0.01,4.15) | 0.35 (0.02,5.97) | 1.03 (0.79,1.33) | 1.48 (0.08,25.83) | **A+C+VKA** |

**Abbreviations:** A+C, aspirin + clopidogrel; A+C+VKA, aspirin + clopidogrel + vitamin K antagonist; A+P+VKA, aspirin + prasugrel + vitamin K antagonist; A+T+VKA, aspirin + ticagrelor + vitamin K antagonist; A+VKA, aspirin + vitamin K antagonist

* Treatments are ordered by SUCRA rank. Comparisons between treatments should be read from column to row for each outcome (row treatment is reference)

## eTable 13.4 Sensitivity analysis of type of stroke: hemorrhagic stroke

The following table shows the effect sizes (risk ratio) and the rank order (SUCRA ranks) compared to aspirin + clopidogrel + vitamin K antagonist

| **Treatment name** | **Risk ratio** | **95% Confidence interval** | **p-value** | **SUCRA rank** |
| --- | --- | --- | --- | --- |
| **A+C** | **0.38** | **0.16,0.92** | **0.003** | 1 |
| A+VKA | 0.99 | 0.02,56.78 | 0.997 | 2 |
| A+C+VKA | reference | | | 3 |
| T+VKA | 1.48 | 0.08,28.05 | 0.780 | 4 |
| A+P+VKA | 16.95 | 0.92,313.80 | 0.057 | 5 |
| **Number of studies** | **6** | **Overall inconsistency**  **chi2 (P value)** | **0.07**  **(P=0.794)** |  |

**Abbreviations:** A+C, aspirin + clopidogrel; A+C+VKA, aspirin + clopidogrel + vitamin K antagonist; A+P+VKA, aspirin + prasugrel + vitamin K antagonist; A+VKA, aspirin + vitamin K antagonist; T+VKA, ticagrelor + vitamin K antagonist

## eFigure 13.7 Network estimated risk ratios (95% confidence intervals) of hemorrhagic stroke

| **A+C** |  |  |  |  |
| --- | --- | --- | --- | --- |
| 0.39 (0.01,24.24) | **A+VKA** |  |  |  |
| 0.26 (0.01,5.57) | 0.67 (0.00,99.73) | **T+VKA** |  |  |
| **0.02 (0.00,0.48)** | 0.06 (0.00,8.60) | 0.09 (0.00,5.50) | **A+P+VKA** |  |
| **0.38 (0.16,0.92)** | 0.99 (0.02,56.78) | 1.48 (0.08,28.05) | 16.95 (0.92,313.80) | **A+C+VKA** |

**Abbreviations:** A+C, aspirin + clopidogrel; A+C+VKA, aspirin + clopidogrel + vitamin K antagonist; A+P+VKA, aspirin + prasugrel + vitamin K antagonist; A+VKA, aspirin + vitamin K antagonist; T+VKA, ticagrelor + vitamin K antagonist

* Treatments are ordered by SUCRA rank. Comparisons between treatments should be read from column to row for each outcome (row treatment is reference)

# Appendix 14

**Comparison-adjusted funnel plot for each outcome form the network meta-analyses**

## eFigure 14.1 Comparison-adjusted funnel plot for the network of major bleeding in all comparisons

**
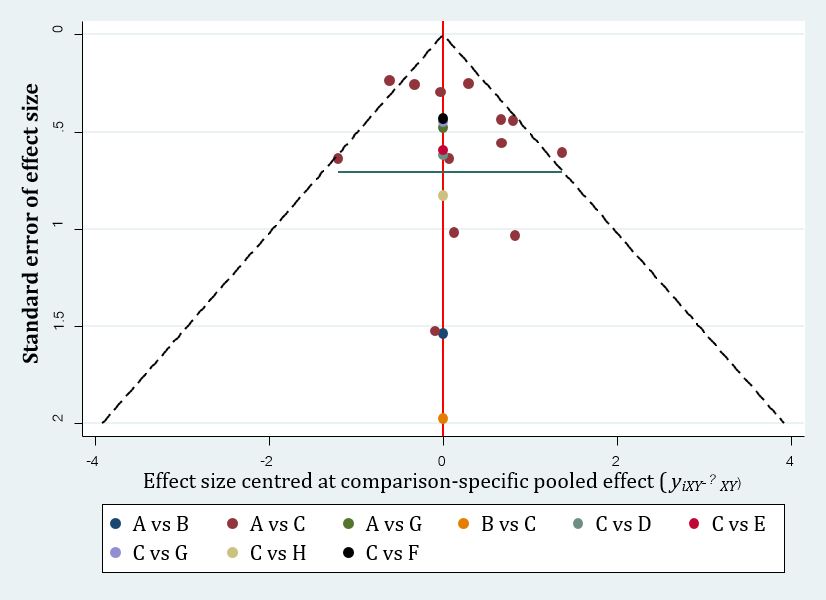
**

**Abbreviations:** A, aspirin + clopidogrel; B, aspirin + clopidogrel + low molecular weight heparin; C, aspirin + clopidogrel + vitamin K antagonist; D, aspirin + prasugrel + vitamin K antagonist; E, aspirin + ticagrelor + vitamin K antagonist; F, aspirin + vitamin K antagonist; G, clopidogrel + vitamin K antagonist; H, ticagrelor + vitamin K antagonist

## eFigure 14.2 Comparison-adjusted funnel plot for the network of stroke and/or systemic embolism in all comparisons

**
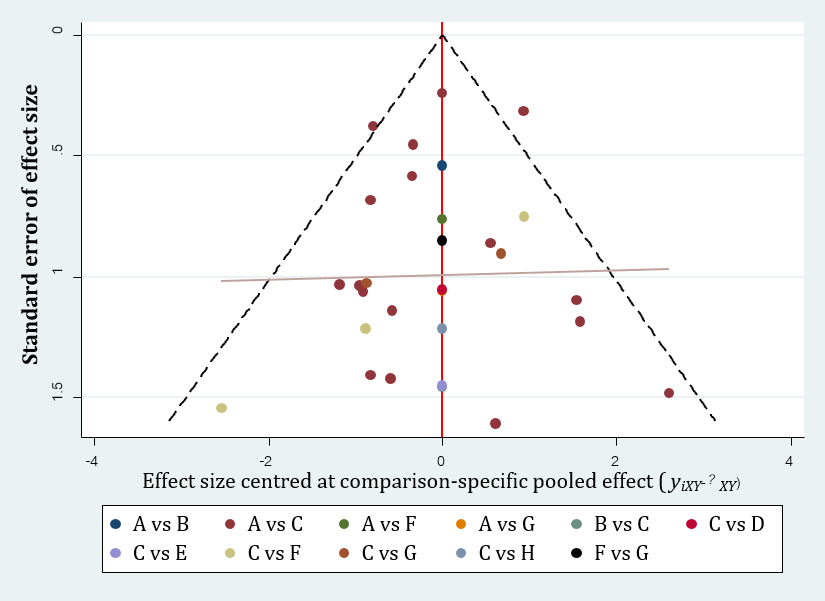
**

**Abbreviations:** A, aspirin + clopidogrel; B, aspirin + clopidogrel + low molecular weight heparin; C, aspirin + clopidogrel + vitamin K antagonist; D, aspirin + prasugrel + vitamin K antagonist; E, aspirin + ticagrelor + vitamin K antagonist; F, aspirin + vitamin K antagonist; G, clopidogrel + vitamin K antagonist; H, ticagrelor + vitamin K antagonist

## eFigure 14.3 Comparison-adjusted funnel plot for the network of myocardial infarction in all comparisons

**
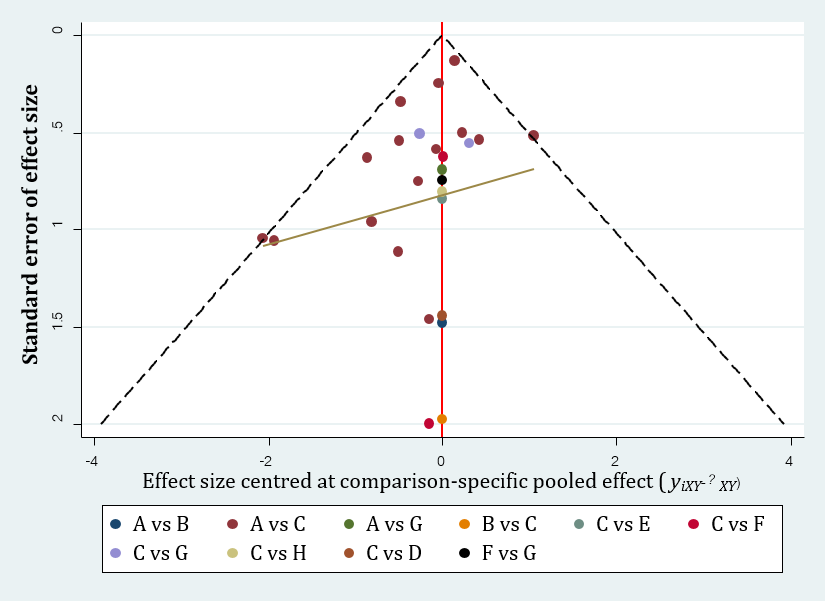
**

**Abbreviations:** A, aspirin + clopidogrel; B, aspirin + clopidogrel + low molecular weight heparin; C, aspirin + clopidogrel + vitamin K antagonist; D, aspirin + prasugrel + vitamin K antagonist; E, aspirin + ticagrelor + vitamin K antagonist; F, aspirin + vitamin K antagonist; G, clopidogrel + vitamin K antagonist; H, ticagrelor + vitamin K antagonist

## eFigure 14.4 Comparison-adjusted funnel plot for the network of repeated revascularization in all comparisons

**
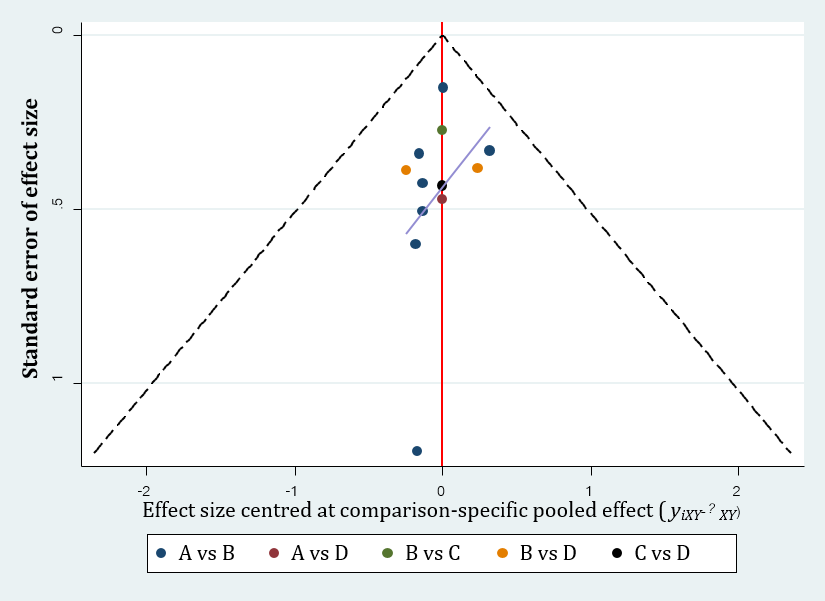
**

**Abbreviations:** A, aspirin + clopidogrel; B, aspirin + clopidogrel + vitamin K antagonist; C, aspirin + vitamin K antagonist; D, clopidogrel + vitamin K antagonist

## eFigure 14.5 Comparison-adjusted funnel plot for the network of stent thrombosis in all comparisons

**
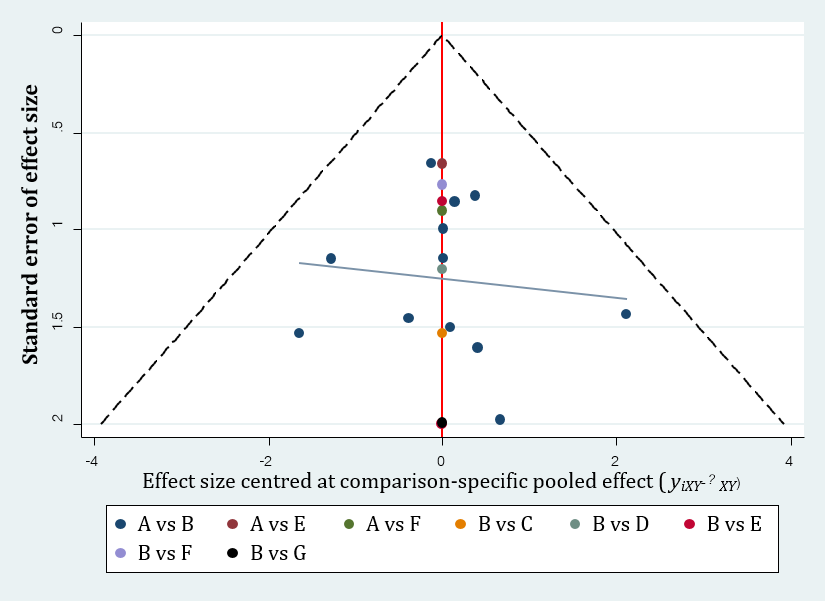
**

**Abbreviations:** A, aspirin + clopidogrel; B, aspirin + clopidogrel + vitamin K antagonist; C, aspirin + prasugrel + vitamin K antagonist; D, aspirin + ticagrelor + vitamin K antagonist; E, aspirin + vitamin K antagonist; F, clopidogrel + vitamin K antagonist; G, ticagrelor + vitamin K antagonist

## eFigure 14.6 Comparison-adjusted funnel plot for the network of all-cause death in all comparisons

**
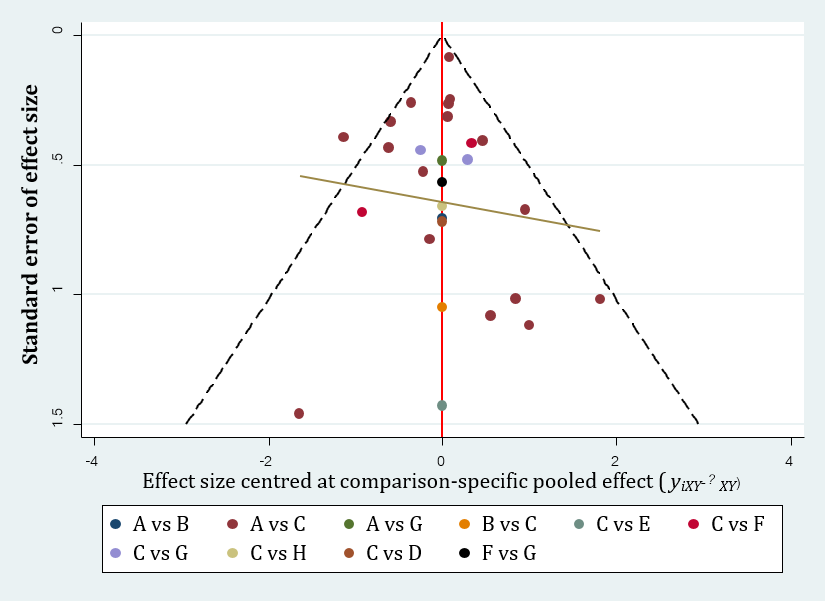
**

**Abbreviations:** A, aspirin + clopidogrel; B, aspirin + clopidogrel + low molecular weight heparin; C, aspirin + clopidogrel + vitamin K antagonist; D, aspirin + prasugrel + vitamin K antagonist; E, aspirin + ticagrelor + vitamin K antagonist; F, aspirin + vitamin K antagonist; G, clopidogrel + vitamin K antagonist; H, ticagrelor + vitamin K antagonist

# Appendix 15

## References of included studies

1. Mehran R, Rao SV, Bhatt DL, Gibson CM, Caixeta A, Eikelboom J, Kaul S, Wiviott SD, Menon V, Nikolsky E, Serebruany V, Valgimigli M, Vranckx P, Taggart D, Sabik JF, Cutlip DE, Krucoff MW, Ohman EM, Steg PG, White H. Standardized bleeding definitions for cardiovascular clinical trials: a consensus report from the Bleeding Academic Research Consortium. Circulation. 2011;123(23):2736-47.

2. Kikkert WJ, van Geloven N, van der Laan MH, Vis MM, Baan J, Jr., Koch KT, Peters RJ, de Winter RJ, Piek JJ, Tijssen JG, Henriques JP. The prognostic value of bleeding academic research consortium (BARC)-defined bleeding complications in ST-segment elevation myocardial infarction: a comparison with the TIMI (Thrombolysis In Myocardial Infarction), GUSTO (Global Utilization of Streptokinase and Tissue Plasminogen Activator for Occluded Coronary Arteries), and ISTH (International Society on Thrombosis and Haemostasis) bleeding classifications. J Am Coll Cardiol. 2014;63(18):1866-75.

3. Vranckx P, Leonardi S, Tebaldi M, Biscaglia S, Parrinello G, Rao SV, Mehran R, Valgimigli M. Prospective validation of the Bleeding Academic Research Consortium classification in the all-comer PRODIGY trial. Eur Heart J. 2014;35(37):2524-9.

4. Dewilde WJ, Oirbans T, Verheugt FW, Kelder JC, De Smet BJ, Herrman JP, Adriaenssens T, Vrolix M, Heestermans AA, Vis MM, Tijsen JG, van 't Hof AW, ten Berg JM. Use of clopidogrel with or without aspirin in patients taking oral anticoagulant therapy and undergoing percutaneous coronary intervention: an open-label, randomised, controlled trial. Lancet. 2013;381(9872):1107-15.

5. Gibson CM, Mehran R, Bode C, Halperin J, Verheugt FW, Wildgoose P, Birmingham M, Ianus J, Burton P, van Eickels M, Korjian S, Daaboul Y, Lip GY, Cohen M, Husted S, Peterson ED, Fox KA. Prevention of Bleeding in Patients with Atrial Fibrillation Undergoing PCI. N Engl J Med. 2016;375(25):2423-34.

6. Cannon CP, Bhatt DL, Oldgren J, Lip GYH, Ellis SG, Kimura T, Maeng M, Merkely B, Zeymer U, Gropper S, Nordaby M, Kleine E, Harper R, Manassie J, Januzzi JL, Ten Berg JM, Steg PG, Hohnloser SH. Dual Antithrombotic Therapy with Dabigatran after PCI in Atrial Fibrillation. N Engl J Med. 2017;377(16):1513-24.

7. Sambola A, Ferreira-Gonzalez I, Angel J, Alfonso F, Maristany J, Rodriguez O, Bueno H, Lopez-Minguez JR, Zueco J, Fernandez-Aviles F, San Roman A, Prendergast B, Mainar V, Garcia-Dorado D, Tornos P. Therapeutic strategies after coronary stenting in chronically anticoagulated patients: the MUSICA study. Heart. 2009;95(18):1483-8.

8. Gao F, Zhou YJ, Wang ZJ, Shen H, Liu XL, Nie B, Yan ZX, Yang SW, Jia de A, Yu M. Comparison of different antithrombotic regimens for patients with atrial fibrillation undergoing drug-eluting stent implantation. Circ J. 2010;74(4):701-8.

9. Rubboli A, Saia F, Sciahbasi A, Bacchi-Reggiani ML, Steffanon L, Briguori C, Calabro P, Palmieri C, Rizzi A, Imperadore F, Sangiorgi GM, Valgimigli M, Carosio G, Steffenino G, Galvani M, Di Pasquale G, La Vecchia L, Maggioni AP, Bolognese L. Outcome of patients on oral anticoagulation undergoing coronary artery stenting: data from discharge to 12 months in the Warfarin and Coronary Stenting (WAR-STENT) Registry. J Invasive Cardiol. 2014;26(11):563-9.

10. Rubboli A, Schlitt A, Kiviniemi T, Biancari F, Karjalainen PP, Valencia J, Laine M, Kirchhof P, Niemela M, Vikman S, Lip GY, Airaksinen KE. One-year outcome of patients with atrial fibrillation undergoing coronary artery stenting: an analysis of the AFCAS registry. Clin Cardiol. 2014;37(6):357-64.

11. De Vecchis R, Cantatrione C, Mazzei D. Clinical Relevance of Anticoagulation and Dual Antiplatelet Therapy to the Outcomes of Patients With Atrial Fibrillation and Recent Percutaneous Coronary Intervention With Stent. J Clin Med Res. 2016;8(2):153-61.

12. Sarafoff N, Martischnig A, Wealer J, Mayer K, Mehilli J, Sibbing D, Kastrati A. Triple therapy with aspirin, prasugrel, and vitamin K antagonists in patients with drug-eluting stent implantation and an indication for oral anticoagulation. J Am Coll Cardiol. 2013;61(20):2060-6.

13. Braun OO, Bico B, Chaudhry U, Wagner H, Koul S, Tyden P, Schersten F, Jovinge S, Svensson PJ, Gustav Smith J, van der Pals J. Concomitant use of warfarin and ticagrelor as an alternative to triple antithrombotic therapy after an acute coronary syndrome. Thromb Res. 2015;135(1):26-30.

14. Fu A, Singh K, Abunassar J, Malhotra N, Le May M, Labinaz M, Glover C, Marquis JF, Froeschl M, Dick A, Hibbert B, Chong AY, So DY. Ticagrelor in Triple Antithrombotic Therapy: Predictors of Ischemic and Bleeding Complications. Clin Cardiol. 2016;39(1):19-23.

15. Nguyen MC, Lim YL, Walton A, Lefkovits J, Agnelli G, Goodman SG, Budaj A, Gulba DC, Allegrone J, Brieger D. Combining warfarin and antiplatelet therapy after coronary stenting in the Global Registry of Acute Coronary Events: is it safe and effective to use just one antiplatelet agent? Eur Heart J. 2007;28(14):1717-22.

16. Suh SY, Kang WC, Oh PC, Choi H, Moon CI, Lee K, Han SH, Ahn T, Choi IS, Shin EK. Efficacy and safety of aspirin, clopidogrel, and warfarin after coronary artery stenting in Korean patients with atrial fibrillation. Heart Vessels. 2014;29(5):578-83.

17. Gilard M, Blanchard D, Helft G, Carrier D, Eltchaninoff H, Belle L, Finet G, Le Breton H, Boschat J. Antiplatelet therapy in patients with anticoagulants undergoing percutaneous coronary stenting (from STENTIng and oral antiCOagulants [STENTICO]). Am J Cardiol. 2009;104(3):338-42.

18. Rubboli A, Magnavacchi P, Guastaroba P, Saia F, Vignali L, Giacometti P, Franco N, Benassi A, Varani E, Campo G, Manari A, De Palma R, Marzocchi A. Antithrombotic management and 1-year outcome of patients on oral anticoagulation undergoing coronary stent implantation (from the Registro Regionale Angioplastiche Emilia-Romagna Registry). Am J Cardiol. 2012;109(10):1411-7.

19. Ho KW, Ivanov J, Freixa X, Overgaard CB, Osten MD, Ing D, Horlick E, Mackie K, Seidelin PH, Dzavik V. Antithrombotic therapy after coronary stenting in patients with nonvalvular atrial fibrillation. Can J Cardiol. 2013;29(2):213-8.

20. Dabrowska M, Ochala A, Cybulski W, Tendera M. Balancing between bleeding and thromboembolism after percutaneous coronary intervention in patients with atrial fibrillation. Could triple anticoagulant therapy be a solution? Postepy Kardiol Interwencyjnej. 2013;9(3):234-40.

21. Hess CN, Peterson ED, Peng SA, de Lemos JA, Fosbol EL, Thomas L, Bhatt DL, Saucedo JF, Wang TY. Use and Outcomes of Triple Therapy Among Older Patients With Acute Myocardial Infarction and Atrial Fibrillation. J Am Coll Cardiol. 2015;66(6):616-27.

22. Kang DO, Yu CW, Kim HD, Cho JY, Joo HJ, Choi RK, Park JS, Lee HJ, Kim JS, Park JH, Hong SJ, Lim DS. Triple antithrombotic therapy versus dual antiplatelet therapy in patients with atrial fibrillation undergoing drug-eluting stent implantation. Coron Artery Dis. 2015;26(5):372-80.

23. Caballero L, Ruiz-Nodar JM, Marin F, Roldan V, Hurtado JA, Valencia J, Manzano-Fernandez S, Sogorb F, Valdes M, Lip GY. Oral anticoagulation improves the prognosis of octogenarian patients with atrial fibrillation undergoing percutaneous coronary intervention and stenting. Age Ageing. 2013;42(1):70-5.

24. Sambola A, Mutuberria M, Garcia Del Blanco B, Alonso A, Barrabes JA, Alfonso F, Bueno H, Cequier A, Zueco J, Rodriguez-Leor O, Bosch E, Tornos P, Garcia-Dorado D. Effects of Triple Therapy in Patients With Non-Valvular Atrial Fibrillation Undergoing Percutaneous Coronary Intervention Regarding Thromboembolic Risk Stratification. Circ J. 2016;80(2):354-62.

25. Maegdefessel L, Schlitt A, Faerber J, Bond SP, Messow CM, Buerke M, Raaz U, Werdan K, Muenzel T, Weiss C. Anticoagulant and/or antiplatelet treatment in patients with atrial fibrillation after percutaneous coronary intervention. A single-center experience. Med Klin (Munich). 2008;103(9):628-32.

26. Sarafoff N, Ndrepepa G, Mehilli J, Dorrler K, Schulz S, Iijima R, Byrne R, Schomig A, Kastrati A. Aspirin and clopidogrel with or without phenprocoumon after drug eluting coronary stent placement in patients on chronic oral anticoagulation. J Intern Med. 2008;264(5):472-80.

27. Manzano-Fernandez S, Pastor FJ, Marin F, Cambronero F, Caro C, Pascual-Figal DA, Garrido IP, Pinar E, Valdes M, Lip GY. Increased major bleeding complications related to triple antithrombotic therapy usage in patients with atrial fibrillation undergoing percutaneous coronary artery stenting. Chest. 2008;134(3):559-67.

28. Ruiz-Nodar JM, Marin F, Hurtado JA, Valencia J, Pinar E, Pineda J, Gimeno JR, Sogorb F, Valdes M, Lip GY. Anticoagulant and antiplatelet therapy use in 426 patients with atrial fibrillation undergoing percutaneous coronary intervention and stent implantation implications for bleeding risk and prognosis. J Am Coll Cardiol. 2008;51(8):818-25.

29. Goto K, Nakai K, Shizuta S, Morimoto T, Shiomi H, Natsuaki M, Yahata M, Ota C, Ono K, Makiyama T, Nakagawa Y, Furukawa Y, Kadota K, Takatsu Y, Tamura T, Takizawa A, Inada T, Doi O, Nohara R, Matsuda M, Takeda T, Kato M, Shirotani M, Eizawa H, Ishii K, Lee JD, Takahashi M, Horie M, Takahashi M, Miki S, Aoyama T, Suwa S, Hamasaki S, Ogawa H, Mitsudo K, Nobuyoshi M, Kita T, Kimura T. Anticoagulant and antiplatelet therapy in patients with atrial fibrillation undergoing percutaneous coronary intervention. Am J Cardiol. 2014;114(1):70-8.

30. Jang SW, Rho TH, Kim DB, Cho EJ, Kwon BJ, Park HJ, Shin WS, Kim JH, Lee JM, Moon KW, Oh YS, Yoo KD, Youn HJ, Lee MY, Chung WS, Seung KB, Kim JH. Optimal antithrombotic strategy in patients with atrial fibrillation after coronary stent implantation. Korean Circ J. 2011;41(10):578-82.

31. Valencia J, Mainar V, Bordes P, Pineda J, Gomez S, Sogorb F. Observance of antiplatelet therapy after stent implantation in patients under chronic oral anticoagulant treatment. J Interv Cardiol. 2008;21(3):218-24.

32. Fiedler KA, Maeng M, Mehilli J, Schulz-Schupke S, Byrne RA, Sibbing D, Hoppmann P, Schneider S, Fusaro M, Ott I, Kristensen SD, Ibrahim T, Massberg S, Schunkert H, Laugwitz KL, Kastrati A, Sarafoff N. Duration of Triple Therapy in Patients Requiring Oral Anticoagulation After Drug-Eluting Stent Implantation: The ISAR-TRIPLE Trial. J Am Coll Cardiol. 2015;65(16):1619-29.

33. Choi HI, Ahn JM, Kang SH, Lee PH, Kang SJ, Lee SW, Kim YH, Lee CW, Park SW, Park DW, Park SJ. Prevalence, Management, and Long-Term (6-Year) Outcomes of Atrial Fibrillation Among Patients Receiving Drug-Eluting Coronary Stents. JACC Cardiovasc Interv. 2017;10(11):1075-85.
